# Polysaccharide dextran-based conjugate for selective co-delivery of two synergistic drugs docetaxel and docosahexaenoic acid to tumor cells

**DOI:** 10.1080/10717544.2022.2152133

**Published:** 2022-12-01

**Authors:** Peng Dong, Hongshuai Lv, Weiping Jia, Jiaojiao Liu, Si Wang, Xiaohai Li, Jinghua Hu, Ling Zhao, Yikang Shi

**Affiliations:** a National Glycoengineering Research Center, Shandong Key Laboratory of Carbohydrate Chemistry and Glycobiology, NMPA Key Laboratory for Quality Research and Evaluation of Carbohydrate Based Medicine, Shandong University, Qingdao, China; b Santolecan Pharmaceuticals LLC, Jupiter, FL, USA

**Keywords:** Docetaxel, dextran, docosahexaenoic acid, conjugate, nanomedicine, combined therapy

## Abstract

Most chemotherapeutic agents are nonspecific distribution and cause systemic toxicities. Polysaccharide-based conjugates are promising strategies to overcome these drawbacks. To this end, two synergistic drugs docetaxel (DTX) and docosahexaenoic acid (DHA) were independently covalently bonded through individual linkers to dextran (100 kDa) to produce a novel dual-drug conjugate dextran–DHA–DTX. The single-drug conjugates dextran–DHA and dextran–DTX were also prepared for comparison. Fluorescent dye Cy7.5-based conjugates dextran–Cy7.5 and dextran–DHA–Cy7.5 were synthesized for cellular uptake study. The dual-drug conjugate dextran–DHA–DTX self-assembled into nanoparticles with the diameter of 102.3 ± 8.3 nm and demonstrated enhanced water solubility and improved pharmacokinetic profiles. Cellular uptake results showed that the dual-drug conjugate entered cells more than the parent DTX by determining the intracellular DTX contents via HPLC/MS analysis and by determining the fluorescent intensity of dextran-Cy7.5 and dextran–DHA–Cy7.5. Importantly, the dual-drug conjugate dextran–DHA–DTX significantly accumulated in tumor tissues and dramatically reduced the DTX concentrations in normal tissues. The dual-drug conjugate completely eradicated all the MCF-7 xenograft tumors without obvious side effects and showed more superior antitumor activity than parent DTX and single-drug conjugate dextran–DTX and dextran–DHA. Both *in vitro* and *in vivo* studies showed that DHA enhanced the antitumor activity of dextran–DTX. The polysaccharide dextran-based dual-drug conjugates may represent an effective way to improve the chemotherapeutic agents.

## 1. Introduction

Docetaxel (DTX) is a semisynthetic chemotherapeutic agent commonly used against a wide range of cancers, including breast, lung, ovarian, prostate, gastric, head and neck cancer (Montero et al., 2005; Razak et al., 2021). DTX is an antimicrotubular agent that mainly exerts a cytotoxic effect by disrupting the microtubule network in cells, thus inhibiting proper cell division. However, the effectiveness of treatment is limited in clinic due to the drawbacks of DTX, such as its low water solubility, nonselective biodistribution, systemic toxicity, and severe allergic reactions (Engels et al., 2007; Picard & Castells, 2015). To overcome these limitations associated with DTX, various kinds of nanoparticles have been formulated for DTX delivery, such as liposomes, dendrimers, micelles, and solid lipid nanoparticles (Zhang et al., 2019; Razak et al., 2021). The nanoparticle delivery systems improve the aqueous solubility of DTX and increase its tissues selectivity through passive and active targeting, which subsequently enhances the antitumor effect and alleviates sides effects on normal tissues. Some DTX-based nanoparticles such as albumin-bound DTX (ABI-008), LE-DT, and BIND-014 failed in clinical trials (He et al., 2019; Zhang et al., 2019; van Eerden et al., 2020; Jurczyk et al., 2022). The formulation of LE-DT was prepared by electrostatic interaction, of which negatively charged synthetic phospholipids and cholesterol and drugs with opposite charge formed stable liposomes (Zhang et al., 2019; Jurczyk et al., 2022). BIND-014 is composed of DTX encapsulated in a polymer core made of a hydrophobic poly(lactide) surrounded by a hydrophilic poly(ethylene glycol) conjugated with a small molecule of prostate-specific membrane antigen (PSMA)-targeting ligands (Jurczyk et al., 2022). New DTX-based formulations currently in clinical trials around the world include DEP docetaxel, CPC634 (CriPec docetaxel), and CRXL301. DEP docetaxel is a PEG–polylysine dendrimer based DTX conjugate (Ekladious et al., 2019; Zhang et al., 2019). CPC634 is a DTX conjugate that consists of monomethoxy poly(ethylene glycol) (mPEG) and *N*-2-hydroxypropyl methacrylamide mono- of dilactate (HPMAmLacn) (Ekladious et al., 2019; Atrafi et al., 2020). CRXL301 is fabricated through encapsulating DTX into cyclodextrin–PEG polymer (Ekladious et al., 2019; Piha-Paul et al., 2021). Up to date, none of new DTX formulations have been approved into clinic application.

Dextran is a natural polysaccharide that is composed of α-1,6-glycosidic linkages in the main chain and α-1,3-glycosidic linkages in the branched chains and similar to muscle glycogen or liver glycogen in structure (K. Liu et al., 2016). Originally dextran was approved as a plasma expander, but its desirable physicochemical characteristics such as water solubility, biocompatibility, biodegradability, and non-immunogenicity, along with its low cost and a history of clinical use make it an attractive system for drug delivery. Plenty of hydroxyl groups and terminal aldehyde groups presented on the dextran backbone provide potential functional sites for drug conjugation through direct or indirect methods (Hong et al., 2018; G. Huang & Huang, 2018; Hu et al., 2021). As drug delivery carrier, some of dextran-based DTX encapsulated and chemically conjugated nanoparticles have showed excellent antitumor activity (Alibolandi et al., 2016; Han et al., 2016; Raza et al., 2016). Similarly, many articles showed that dextran-based paclitaxel nanoparticles also exhibited superior antitumor effect than parent paclitaxel (Sugahara et al., 2007; P. Liu et al., 2015; Zhou et al., 2017; Chang et al., 2020).

Docosahexaenoic acid (DHA) is a long-chain omega-3 polyunsaturated fatty acid that is a critical component of lipid of cell membrane. DHA plays many physiologic roles including regulation of membrane fluidity, neurotransmitter release, gene expression, myelination, and cell differentiation and growth (Weiser et al., 2016). Importantly, it is well known that DHA alone or in combination with chemotherapeutic agents induce cell cycle arrest, apoptosis, autophagy, and tumor growth inhibition by multiple mechanisms such as influencing cell membrane lipid composition, especially that of lipid raft, which leads to a significant modification of their physical–chemical properties altering the content and function of transmembrane proteins including receptors, growth factors, and ATP-binding cassette transporters (Siddiqui et al., 2011; Corsetto et al., 2017; Giordano et al., 2020; Chen et al., 2021; Y. Liu et al., 2021; Fodil et al., 2022; L. Huang et al., 2022). Many clinical trials revealed that supplementation with DHA/Eicosapentaenoic acid during cancer treatment could improve a variety of outcomes important to the patient and the disease process, including immune system modulation, improved weight maintenance, and increased disease-free or progression-free survival (Newell et al., 2021). DHA also increases the sensitivity of cancer cells to antitumor drugs such as DTX and paclitaxel (Chauvin et al., 2016; Song & Kim, 2016; Corsetto et al., 2017; Goupille et al., 2020; Newell et al., 2022). Therefore, a conjugate DHA–paclitaxel (Taxoprexin) has been investigated in clinical trials (Homsi et al., 2010; Bedikian et al., 2011). Some DTX-based DHA-containing nanoparticles exhibited excellent therapeutic efficacy against tumors (Jiang et al., 2018; Wang et al., 2021; Li et al., 2022).

We have previously produced a dual-drug conjugate dextran–DHA–DTX by first connecting DHA and DTX to form DHA–DTX, and then grafting DHA–DTX onto dextran (Dong et al., 2022). However, in this study, a dual-drug conjugate dextran–DHA–DTX was produced by separately connecting DHA and DTX with dextran through individual linkers, which was completely different in chemical structure from tandem type of dual-drug conjugate we reported previously. A single-drug conjugates dextran–DHA and dextran–DTX were also fabricated to compare the antitumor activities among the single- and dual-drug conjugates. The dual-drug conjugate exhibited improved pharmacokinetic profiles, selective tumor accumulation, and superior antitumor effect in both xenografted mice models. The strategy to construct dextran-based dual-drug conjugates is a promising approach to obtain a drug candidate of new DTX formulation.

## 2. Materials and methods

### 2.1. Chemical reagents

Dextran (*M*_r_ = 100 kDa) was purchased from Sigma-Aldrich Inc. DTX was purchased from Wuxi Zishan Pharmaceutical Co., Ltd. DHA was obtained from Aladdin Reagent (Shanghai) Co., Ltd. All other reagents were of analytical or chromatographic reagent quality and were purchased from commercial sources.

### 2.2. Cell lines and cell culture

The human breast cancer cell line MCF-7, and mouse triple-negative breast cancer cells 4T1, human lung cancer cells H460 were purchased from the Shanghai Institute of Biochemistry and Cell Biology, Chinese Academy of Sciences. All cell lines were cultured in standard medium according to the manufacturer’s instructions containing 10% FBS and antibiotics (penicillin and streptomycin), and maintained in a humidified atmosphere of 5% CO_2_ at 37 °C.

### 2.3. Sulforhodamine B assay

Cytotoxicity was assessed using sulforhodamine B (SRB) assay. In brief, cells were cultured in a 96-well plate for 24 h and then incubated with drugs for the desired periods. Cells were fixed with 10% trichloroacetic acid (wt/vol) for 1 h at 4 °C, washed with water, and air-dried. Briefly, 100 μL of SRB solution (0.4% [wt/vol] in 1% acetic acid) was added to stain the cells for 20 min at room temperature. After staining, unbound dye was removed by washing five times with 1% acetic acid and the plates were air dried. The absorbance of the protein-bound dye was measured at 515 nm on a Bio-Rad 550 ELISA microplate reader. Cell viability was normalized to the control group.

### 2.4. Determination of DTX contents in the conjugate

The dextran–DHA–DTX conjugate completely released the C-13 side chains of DTX under alkaline conditions and the C-13 side chains were used to measure DTX contents in the conjugate. Briefly, the conjugate was incubated with 0.5 mol/L of sodium hydroxide solution (methanol:water = 1:1) at room temperature with shaking at 200 rpm. After incubation for 3 h, the C-13 side chains were absolutely released from the conjugate. The solution was adjusted to pH 6.0 by adding glacial acetic acid and then applied for High Performance Liquid Chromatography/Mass Spectra (HPLC/MS) analysis. The peak area of the C-13 side chains was recorded. At the same time, parent DTX was hydrolyzed at the similar procedure to obtain the standard curve of DTX amount and the integrated peak area of the C-13 side chain. Samples were performed on Agilent 7890A HPLC and mass spectrometer SCIEX TripleQuad 5500 at 254 nm wavelength by using Agilent C18 column (3.5 µm, 2.1 × 100 mm).

### 2.5. In vitro drug release under physiological condition

Briefly, 1 mL of the conjugate or parent DTX were placed into a dialysis bag (MWCO: 5000 Da) and incubated in 19 mL of phosphate buffer (pH7.4, 10 mM, containing 1.0% Tween 80) at 37 °C with slight shaking. At predetermined time points, 1 mL of medium was taken and 1 mL of fresh phosphate buffer was replaced. The accumulative release percentage of DTX was quantified through HPLC analysis.

### 2.6. DTX release from the conjugate in plasma

To investigate the profile of DTX release from the conjugate, released DTX and total DTX concentrations were measured after the conjugate was incubated with rat plasma. Briefly, 900 μL of rat plasma was mixed with 100 μL of the conjugate (10 mg/mL in PBS) and incubated at 37 °C under shaking. The incubation was stopped by adding protein precipitation solution (acetonitrile:methanol = 1:1) into samples at different time points. The mixture was rigorously vortexed and put on ice for 5 min and then was centrifuged at 10,000 rpm for 15 min. After being filtered with 0.22-μm membrane filter, the supernatant was analyzed by RP-HPLC (Agilent 1220 Infinity II) with C18 column.

### 2.7. Determination of released DTX and total DTX contents in cancer cells

Cancer cells were cultured in 10-cm dishes for 24 h and then treated with parent DTX and the conjugate at the final concentration of 200 ng/mL equivalent to DTX. Cells were collected and washed with cold PBS after treatment for different times. (1) For released DTX analysis, 1 mL of mixed solution of acetonitrile:PBS (1:1) containing internal standard paclitaxel was added into the cells. After sonicated for 2 min, the samples were centrifuged and supernatant was filtered with a 0.22-μm membrane for HPLC/MS analysis to test the peak area of DTX. (2) For total DTX analysis, total DTX amount was calculated according on the C-13 side chain released from DTX under alkaline conditions. Briefly, 1 mL of mixed solution of acetonitrile:PBS (1:1) containing internal standard Boc-l-phenylalanine was added into cells. After sonicated for 2 min, the samples were centrifuged and supernatant was filtered with a 0.22-μm membrane and then was hydrolyzed through adding sodium hydroxide aqueous solution at the final concentration of 0.5 mol/L. The following procedures were the same as those in section 2.4.

### 2.8. Determination of released DTX and total DTX contents in plasma and tumor tissues

The Laboratory Animal Ethical and Welfare Committee of Shandong University has approved all experiments of the animals in this study. BALB/C mice bearing 4T1 cells were used to assess the pharmacokinetics and the biodistribution of the conjugate. When the tumor grew to 200 mm^3^, mice were randomly divided into several groups (*n* = 5 in each group) and were intravenously administered with both parent DTX and the conjugate at the dose 12 mg/kg equivalent to DTX. After treatment for different time, the mice were sacrificed, blood and tissues were obtained. Blood was collected in a heparinized tube and centrifuged at 3000 rpm for 10 min. The supernatant of blood and tissues were mixed with PBS (pH7.4) and homogenized on ice. The mixture was used for next quantitative analysis of released DTX and total DTX.

To measure the amount of released DTX, homogenized blood or tumor samples were added with three times volumes of methanol:acetonitrile (1:1) solution containing internal standard paclitaxel. The mixture was centrifuged at 12,000 rpm and the supernatant was filtered with 0.22-μm membrane filter for HPLC/MS analysis.

To measure the amount of total DTX, the C-13 side chain released from DTX was determined. Briefly, homogenized blood or tumor samples were added with three times volumes of methanol:acetonitrile (1:1) solution containing internal standard Boc-l-phenylalanine. The mixture was centrifuged at 12,000 rpm and the supernatant was added with sodium hydroxide aqueous solution at a final concentration of 0.5 mol/L. The following procedures were the same as those in section 2.4.

### 2.9. Cellular uptake of dextran–Cy7.5 and dextran–DHA–Cy7.5

After MCF-7 cells were cultured for 24 h, florescent dye 7.5, dextran–Cy7.5, and dextran–DHA–Cy7.5 were incubated for various time points at the final concentration of 5 µg/mL in culture medium. After washing three times with PBS, the florescent intensity of samples was detected through Flow cytometry (Becton-Dickinson Co., USA).

### 2.10. Inhibition of tumor growth in vivo

BALB/c nude mice bearing H460 and MCF-7 were selected as the animal models, respectively. Cancer cells H460 and MCF-7 (5.0 × 10^6^) in 100 μL of PBS were injected at right axillary subcutaneously. When the tumors grew to 100 or 190 mm^3^, the mice were divided randomly into different groups (*n* = 6 in each group). The control group received PBS only. The dose of parent DTX, dual-drug conjugate dextran–DHA–DTX, single-drug conjugate dextran–DTX groups were 6 mg/kg or 12 mg/kg equivalent to DTX. The drugs were injected via tail vein once a week for 4 weeks in H460 model and for 3 weeks in MCF-7 model. Tumor volume and body weight were recorded twice a week. The volume of tumor was calculated according to this equation: (length × width^2^)/2, that the longest diameter was described as length and the widest diameter was described as width, respectively.

### 2.11. Histological examination of tissues

Tissues were fixed in 4% formaldehyde and embedded in paraffin. Briefly, 5 μm sections of tissues were stained through hematoxylin and eosin (H&E) staining.

### 2.12. Statistical analysis

The statistical differences between different groups were calculated by the Student’s *t* test. *p* < .05 were considered statistically significant. All values were expressed as means ± SD (standard deviation).

## 3. Results and discussion

### 3.1. Chemical synthesis and characterization of the conjugate 18

**Scheme 1.**
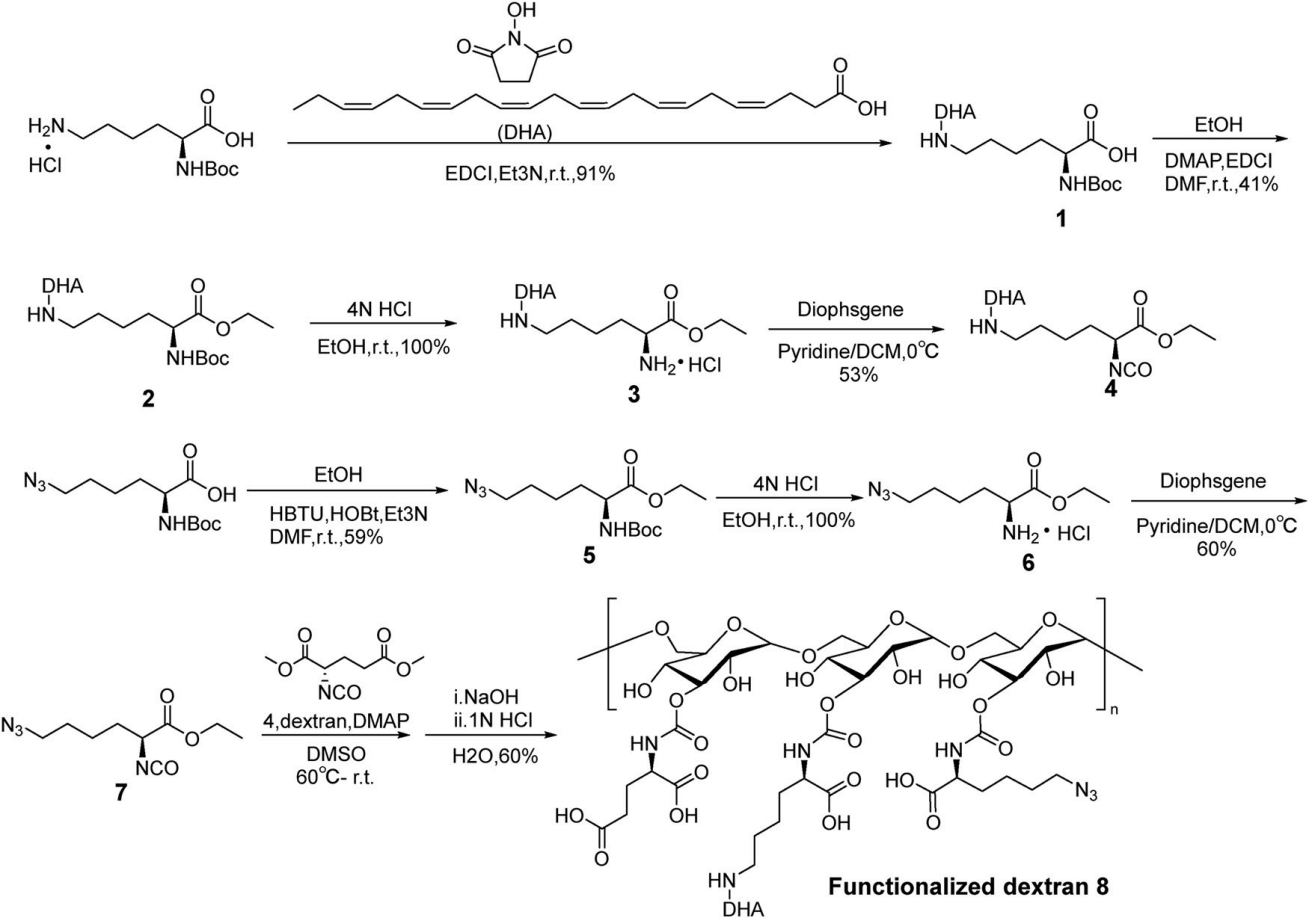
The synthetic route of functionalized dextran **8**.

**Scheme 2.**
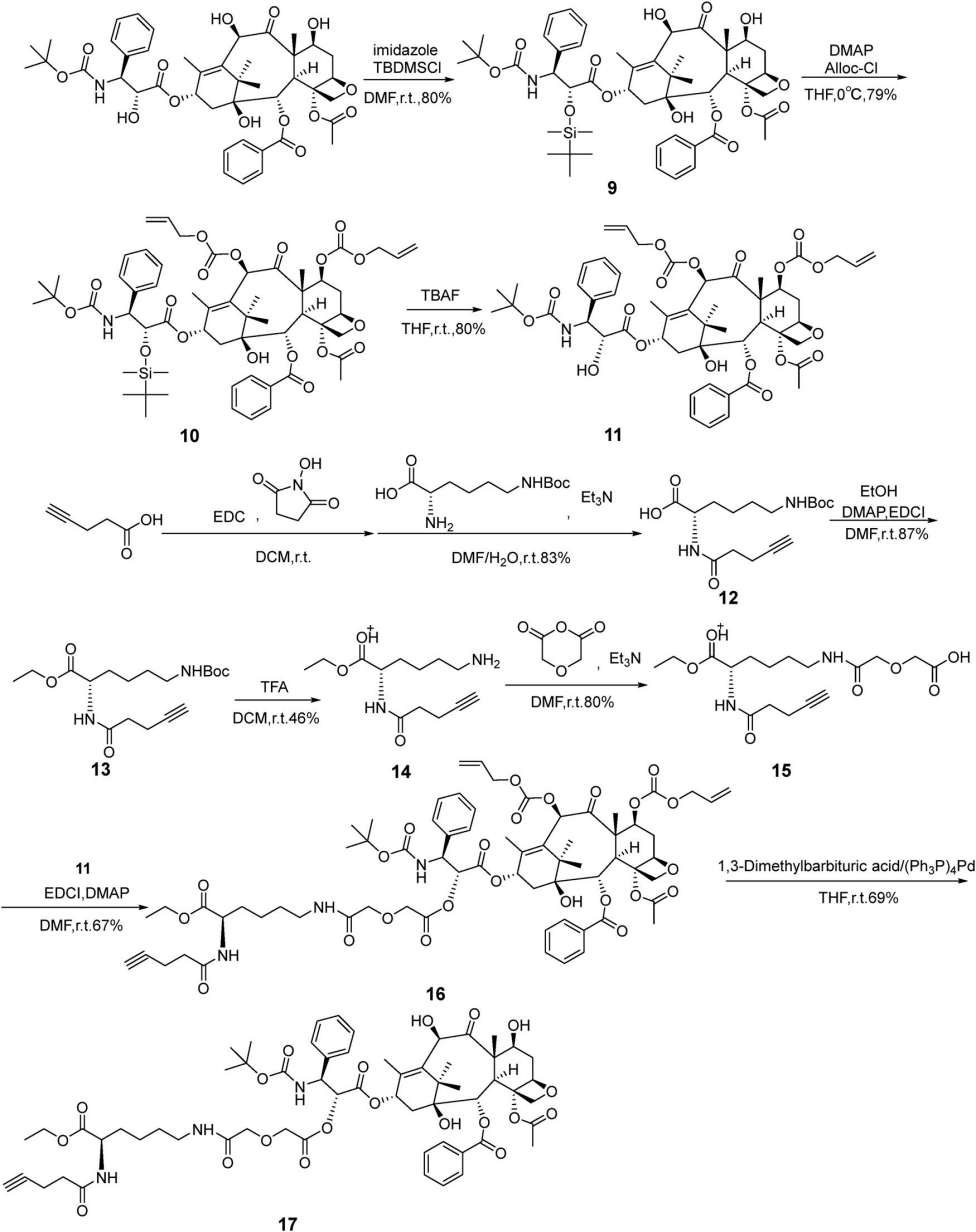
The synthetic route of DTX-linker **17**.

**Scheme 3.**
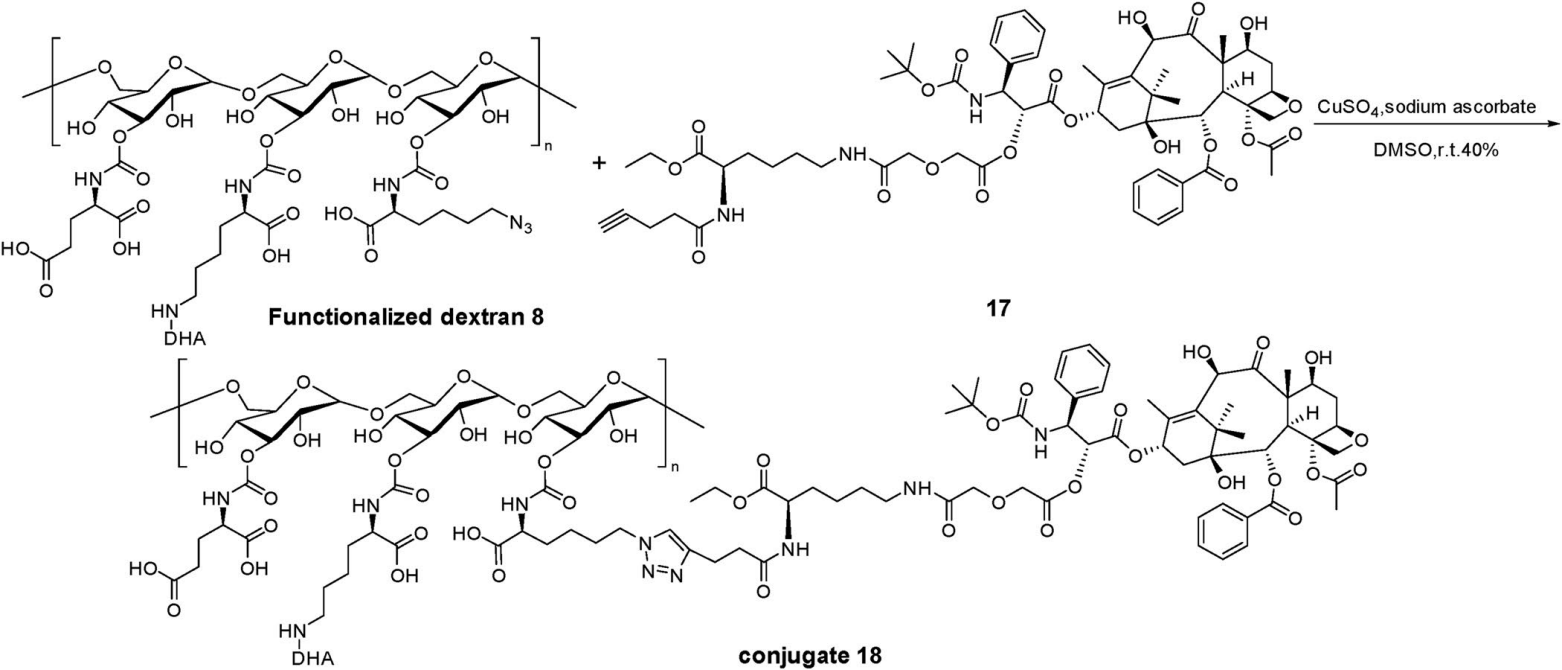
The synthetic route of dual-drug conjugate **18**.

The synthetic procedures in details were described in Supplementary Materials. First, lysine-modified DHA (compound **4**), functionalized groups (compound **7**) were synthesized, respectively. Dimethyl (*S*)-2-isocyanatopentanedioate, compound **4** and **7** were connected with dextran (100 kDa) to produce a functionalized dextran (compound **8**, Scheme 1). Glutamic acid with two carboxyl group endowed the dextran carrier with negative charges. Azide lysine would provide azide for the connection with DTX. Second, 7′-OH and 10′-OH of DTX were protected and 2′-OH of DTX specifically connected with a linker to form DTX-linker which provides alkyne group (compound **17**, Scheme 2). Third, DTX-linker was coupled the functionalized dextran to produce the final dual-drug conjugate dextran–DHA–DTX **18** (Scheme 3). The preparation and confirmation of dextran–DTX, dextran–DHA, dextran–DHA–Cy7.5, and dextran–Cy7.5 were also completed (Scheme S1–S3, Figure S1–S3, Supplementary data).

**Figure 1.**
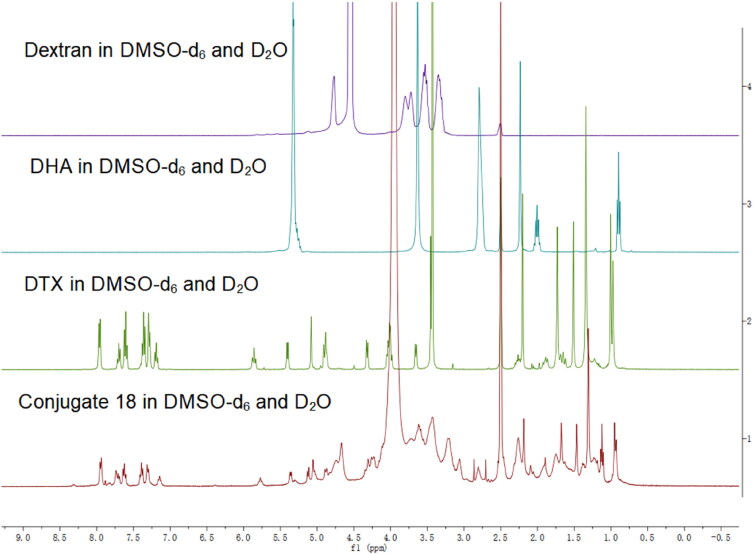
^1^H spectra of dextran, DHA, DTX, and conjugate dextran–DHA–DTX **18**.

**Figure 2.**
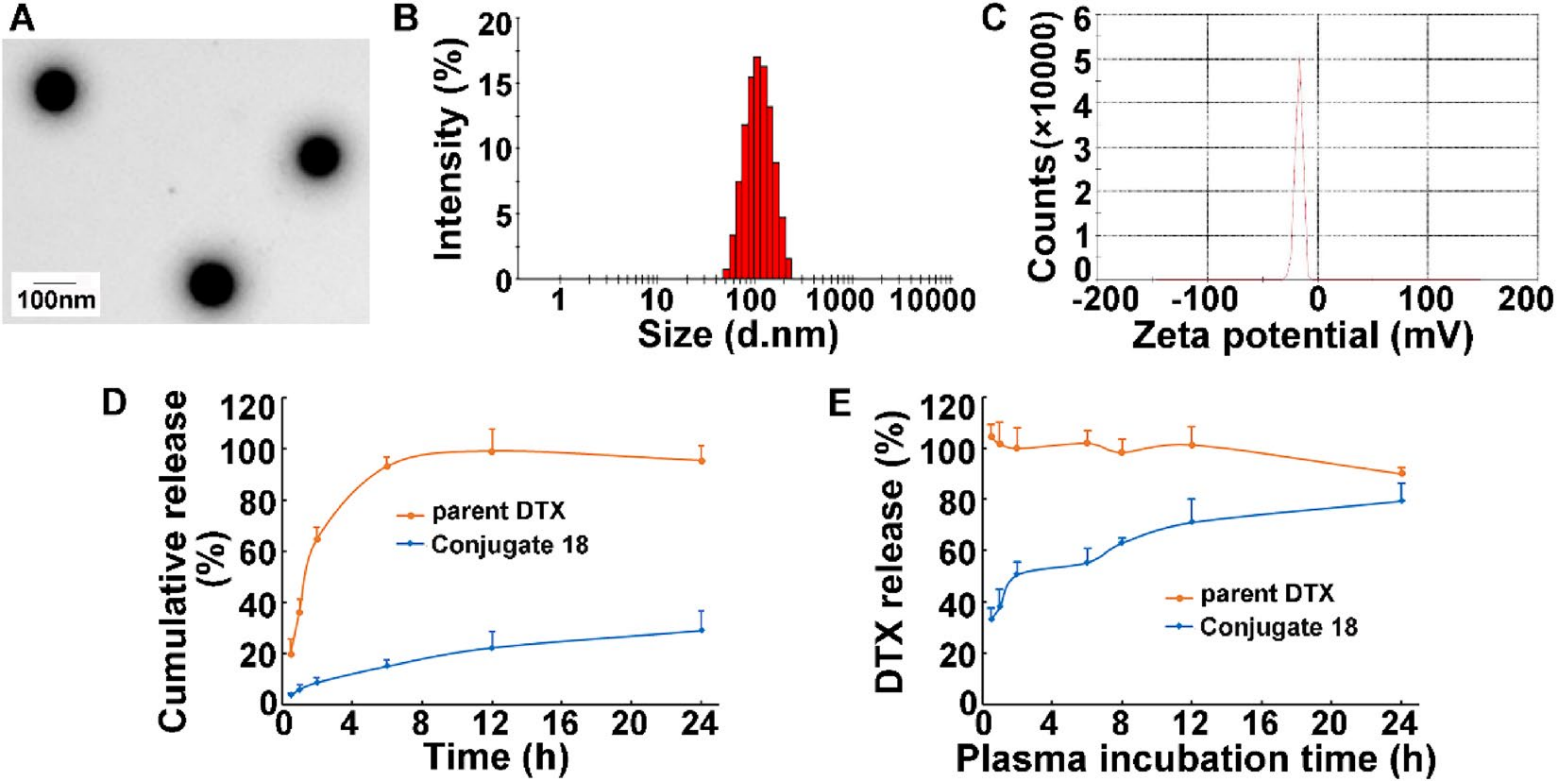
Characterization of the conjugate dextran–DHA–DTX **18**. (A) TEM images of the conjugate **18** (1 mg/mL). (B) Size distribution of the conjugate **18**. (C) Surface charge of the conjugate **18** (1 mg/mL). (D) DTX release from the conjugate **18** in PBS solution (pH7.4). (E) DTX release from the conjugate **18** in rat plasma. Data were presented as mean ± SD (*n* = 3).

The structure of the conjugate dextran–DHA–DTX **18** was confirmed by ^1^H NMR spectroscopy as compared with dextran, DTX, and DHA (Figure 1). The purity of the conjugate **18** was measured by Size Exclusion Chromatography-HPLC (SEC-HPLC) and found to be greater more than 98%. The DTX drug loading capacity of the conjugate **18** was 18.73 ± 2.43% (wt/wt) which was calculated through examining the released C13 side chain of DTX by alkyline hydrolysis of the conjugate. The DHA content was quantified as 13.22 ± 1.87% depending on ^1^H NMR of the conjugate **18**. The conjugate **18** can be easily dissolved in water while yielding a light-yellow solution. The water solubility of the conjugate was 37.6 ± 3.5 mg/mL (equivalent to DTX), which was much higher than that of parent DTX (3–7 μg/mL) (Du et al., 2007; Engels et al., 2007). Transmission electron microscope image displayed that the conjugate **18** had a spherical shape in aqueous solution with the diameter of 102.3 ± 8.3 nm (Figure 2(A,B)). The surface charge of the conjugate **18** was –17.5 ± 3.5 mV (Figure 2(C)).

The DTX release profile of the conjugate **18** was determined upon incubation with PBS and plasma, respectively (Figure 2(D,E)). The results of both DTX release showed that the conjugate **18** had the ability to release the DTX from conjugate **18** and the conjugate **18** released DTX faster in plasma than in PBS solution (pH7.4) owing to the fact that there were a variety of enzymes in the plasma to hydrolyze the ester bond and amide bond of the conjugate.

### 3.2. In vitro cytotoxicity of the conjugate 18

**Figure 3.**
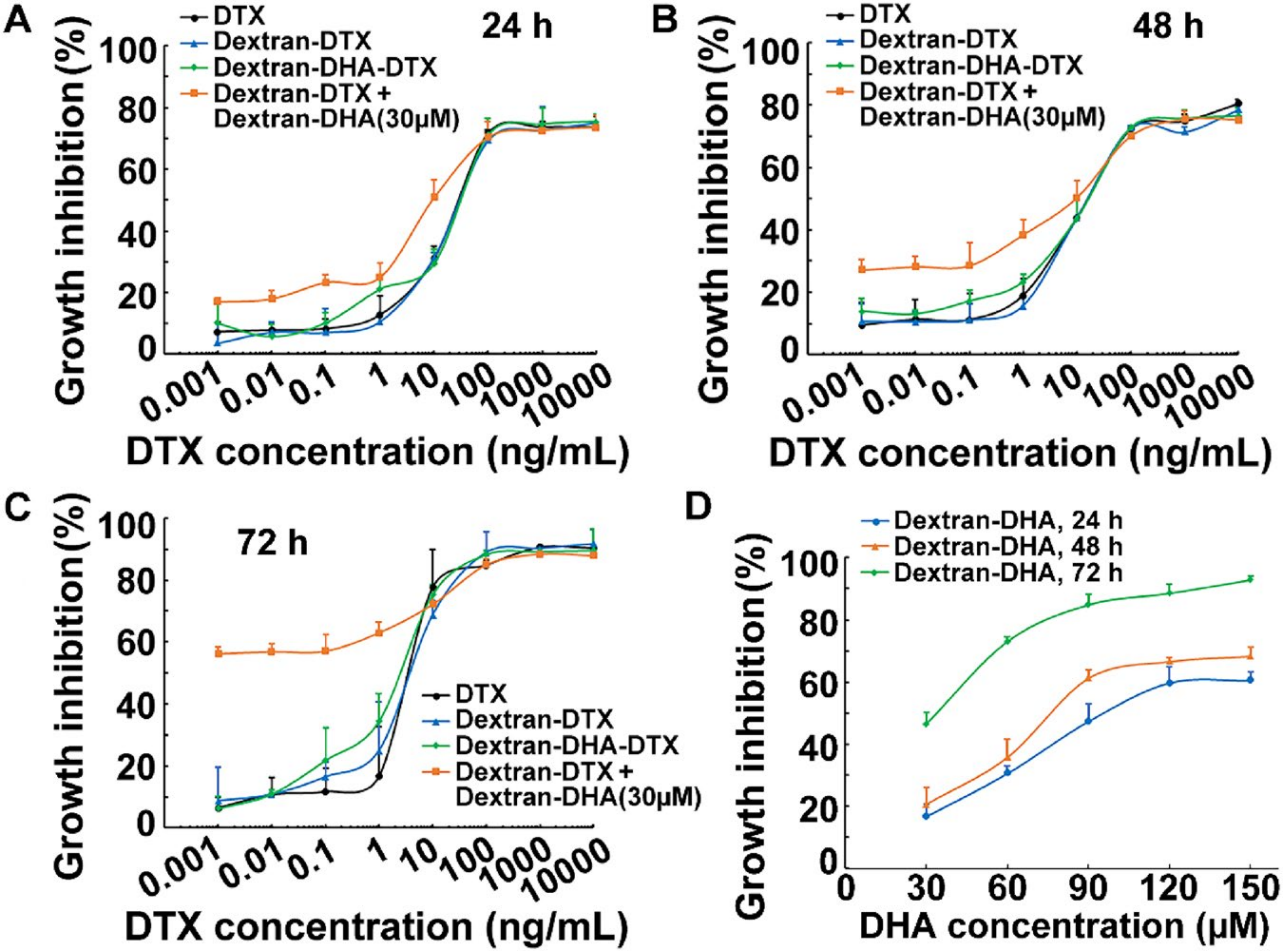
Cytotoxicity of the conjugate **18** in breast cancer cells MCF-7. (A–C) Conjugates inhibited MCF-7 cells growth for 24 h (A), 48 h (B), and 72 h (C), respectively. (D) The conjugate dextran–DHA inhibited MCF-7 cells growth. DTX concentrations of the conjugate dextran–DTX and dextran–DHA–DTX were equivalent to DTX. DHA concentrations of the conjugate dextran–DHA were equivalent to DHA calculated depending on the loading capacity. Data were presented as mean ± SD (*n* = 3).

Breast cancer cells MCF-7 viability was measured after exposure to parent DTX, single-drug conjugate dextran–DTX, and dual-drug conjugate dextran–DHA–DTX **18**, respectively. The results showed that parent DTX, single-drug, and dual-drug conjugates inhibited cell growth in both a dose- and time-dependent manner (Figure 3(A–C)). The dual-drug conjugate dextran–DHA–DTX **18** slightly inhibited cell growth more than DTX and dextran–DTX after cells were treated for 24 h, 48 h, and 72 h, respectively. For example, the IC_50_ dose of DTX in dextran–DHA–DTX was 1.93 ± 0.90 ng/mL which was not significantly different from 3.78 ± 1.05 ng/mL and 3.50 ± 0.69 ng/mL of IC_50_ doses for parent DTX and dextran–DTX after treatment for 72 h, suggesting that the conjugate **18** did not exhibit more cytotoxicity than the parent DTX *in vitro*.

The effects of dextran–DHA alone and in combination with dextran–DTX on cell viability were also evaluated. Cell growth was dose- and time-dependently inhibited after MCF-7 cells were exposed to dextran–DHA at the concentrations of 30, 60, 90,120, and 150 µM (equivalent to DHA, Figure 3(D)). Treatment of 30 µM dextran–DHA and 0.001 ng/mL dextran–DTX alone for 72 h inhibited cell proliferation by 46.5% and 8.7%, respectively; however, the combined treatment at the same concentration inhibited cell proliferation by 56.3%, which was greater than individual treatment. When the concentration of dextran–DTX was lower than 1 ng/mL (equivalent to DTX), combined treatment inhibited more cell growth than single treatment (Figure 3(C,D). Taken together, these above data suggested that dextran–DHA enhanced the cytotoxicity of dextran–DTX in lower concentrations of dextran–DTX.

### 3.3. Cellular uptake of the conjugate

**Figure 4.**
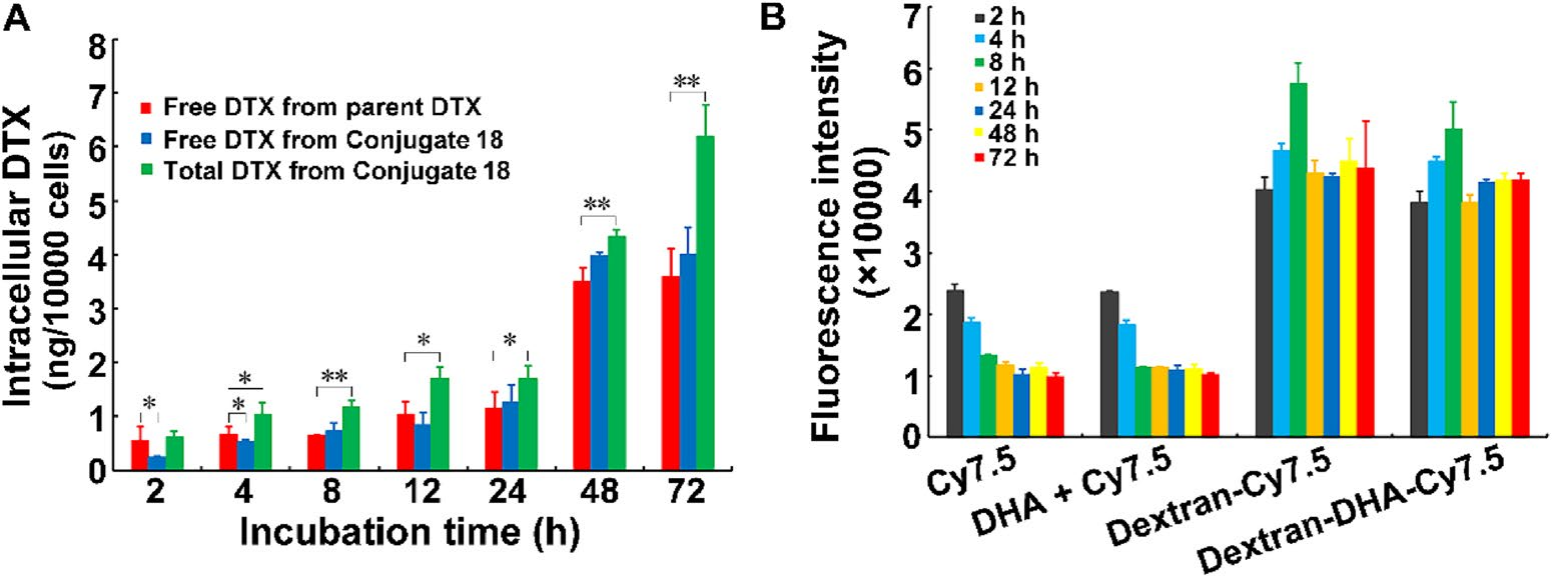
Cellular uptake of dextran-based conjugates. (A) Free DTX and total DTX content in MCF-7 cells after incubation with parent DTX and the conjugate dextran–DHA–DTX **18**, respectively. (B) Fluorescent intensity of Cy7.5 dye and its conjugates in MCF-7 cells. MCF-7 cells were incubated with 5 µg/mL Cy7.5 and its conjugates (equivalent to Cy7.5) for indicated time points, respectively. Data were presented as mean ± SD (*n* = 3). *p < .05, **p < .01.

Intracellular DTX concentrations were detected after MCF-7 cells were exposed to 200 ng/mL dual-drug conjugate **18** at different times. The intracellular total DTX contents in conjugate-treated cells were significantly higher than intracellular DTX contents in parent DTX-treated cells after 4 h of treatment, showing that more DTX penetrated into cells upon exposure to conjugate in comparison with exposure to parent DTX (Figure 4(A)). The intracellular total DTX (released DTX and still bounded DTX) contents were obviously higher than intracellular free DTX in the conjugate-treated cells throughout 72 h of treatment. Interestingly, the free DTX contents in conjugate-treated cells were significantly lower than the DTX contents in parent DTX-treated cells when cells were treated for 2 h and 4 h. Aforementioned results demonstrated that DTX was not rapidly released from the conjugate after the conjugate entered the cells.

To investigate if the dual-drug conjugate enters cells more than the parent DTX, the fluorescent dye Cy7.5 was covalently grafted to dextran to produce two conjugates termed as dextran-Cy7.5 and dextran-DHA-Cy7.5, respectively. Both conjugates and individual Cy7.5 dye were incubated with MCF-7 cells at indicated time points. The final concentration of Cy7.5 or equivalent Cy7.5 for conjugate in culture medium was 5 µg/mL. As compared with individual Cy7.5 treatment, both the dextran–Cy7.5 and dextran–DHA–Cy7.5 treatments dramatically resulted in higher fluorescent intensity throughout the period of treatment, suggesting that dextran-based conjugates penetrate into cells much more than the parent chemical agents (Figure 4(B)). No significant difference in fluorescent intensity was found between the treatment of dextran–Cy7.5 and dextran–DHA–Cy7.5 at the same time point. Combined treatment of Cy7.5 with 50 mmol/L DHA did not increase the fluorescent signal compared to the individual Cy7.5 treatment, indicating that 50 mmol/L DHA did not influence cellular uptake of Cy7.5.

### 3.4. Plasma pharmacokinetics and biodistribution of the conjugate

**Figure 5.**
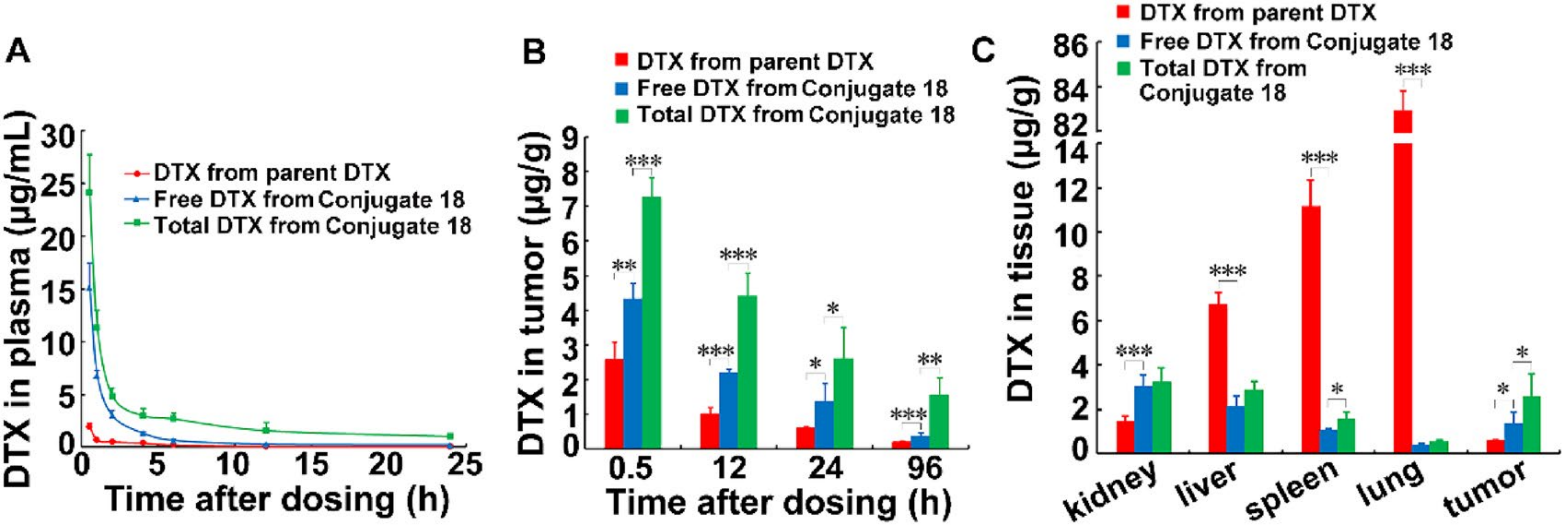
Pharmacokinetics and distribution of the conjugate **18** in xenograft mice bearing 4T1 cells. (A) Free DTX and total DTX contents in plasma. (B) Free DTX and total DTX contents in tumor after mice were injected with parent DTX and the conjugate **18**. (C) Free DTX and total DTX contents in tissues after mice were injected with the conjugate **18** for 24 h. *p < .05, ***p < .001. Data were presented as mean ± SD (*n* = 5).

For pharmacokinetics and biodistribution analysis, DTX and the dual-drug conjugate **18** at equivalent dose of 12 mg/kg DTX were intravenously injected into BALB/c mice bearing breast cancer cells 4T1. Blood samples and tissues were harvested at different time points, and were analyzed by HPLC/MS for both free DTX and total DTX. The plasma concentration–time profiles and pharmacokinetic parameters of the conjugate **18** were illustrated in Figure 5(A) and Table 1. The decline in plasma concentrations following intravenous administration of parent DTX and the conjugate **18** was described by two compartment open model. In term of both released DTX and total DTX in plasma, the conjugate **18** treatment significantly increased *T*_1/2 α_, and AUC (0-∞) as compared with the parent DTX treatment within 24 h. The total DTX concentration was higher than the released DTX in the conjugate **18** group, indicating that there was still DTX coupled with dextran in plasma. The clearance rate of the released DTX and total DTX in plasma of conjugate-treated mice was significantly declined compared to parent DTX-treated mice. All aforementioned results revealed that the conjugate possessed superior pharmacokinetic profiles than parent DTX.

**Table 1.** Pharmacokinetic parameters for the conjugate 18.

	DTX	Conjugate 18 (released DTX)	Conjugate 18 (total DTX)
*T*_1/2 α_ (h)	0.13 ± 0.01	0.37 ± 0.05**	0.41 ± 0.05***
*T*_1/2 β_ (h)	4.82 ± 0.64	2.44 ± 0.37**	10.19 ± 1.62**
CL(L/h/kg)	1.66 ± 0.16	0.27 ± 0.02***	0.12 ± 0.02***
AUC (0-∞) (μg/mL × h)	7.27 ± 0.71	44.00 ± 3.10***	103.86 ± 12.36***

AUC, area under the curve; *t*_1/2_, half-life time; CL, clearance.

**p < .01, ***p < .001 versus DTX group, *n* = 5.

Intratumoral released DTX in conjugate-treated mice significantly increased as compared to parent DTX-treated mice up to 96 h (Figure 5(B)). The total DTX contents in tumor were also dramatically higher than the free DTX released from the conjugate **18**. The total DTX contents in tumors were 2.84, 4.45, 4.40, and 8.27-fold higher with conjugate treatment compared to that with parent DTX treatment at 0.5, 12, 24 and 96 h, respectively. Notably, the free DTX released from the conjugate **18** was significantly lower than that from parent DTX in normal tissues such as liver, spleen, and lung (Figure 5(C)). These results demonstrated that the conjugate **18** selectively accumulated in tumors.

### 3.5. In vivo antitumor activity of the conjugate 18

**Figure 6.**
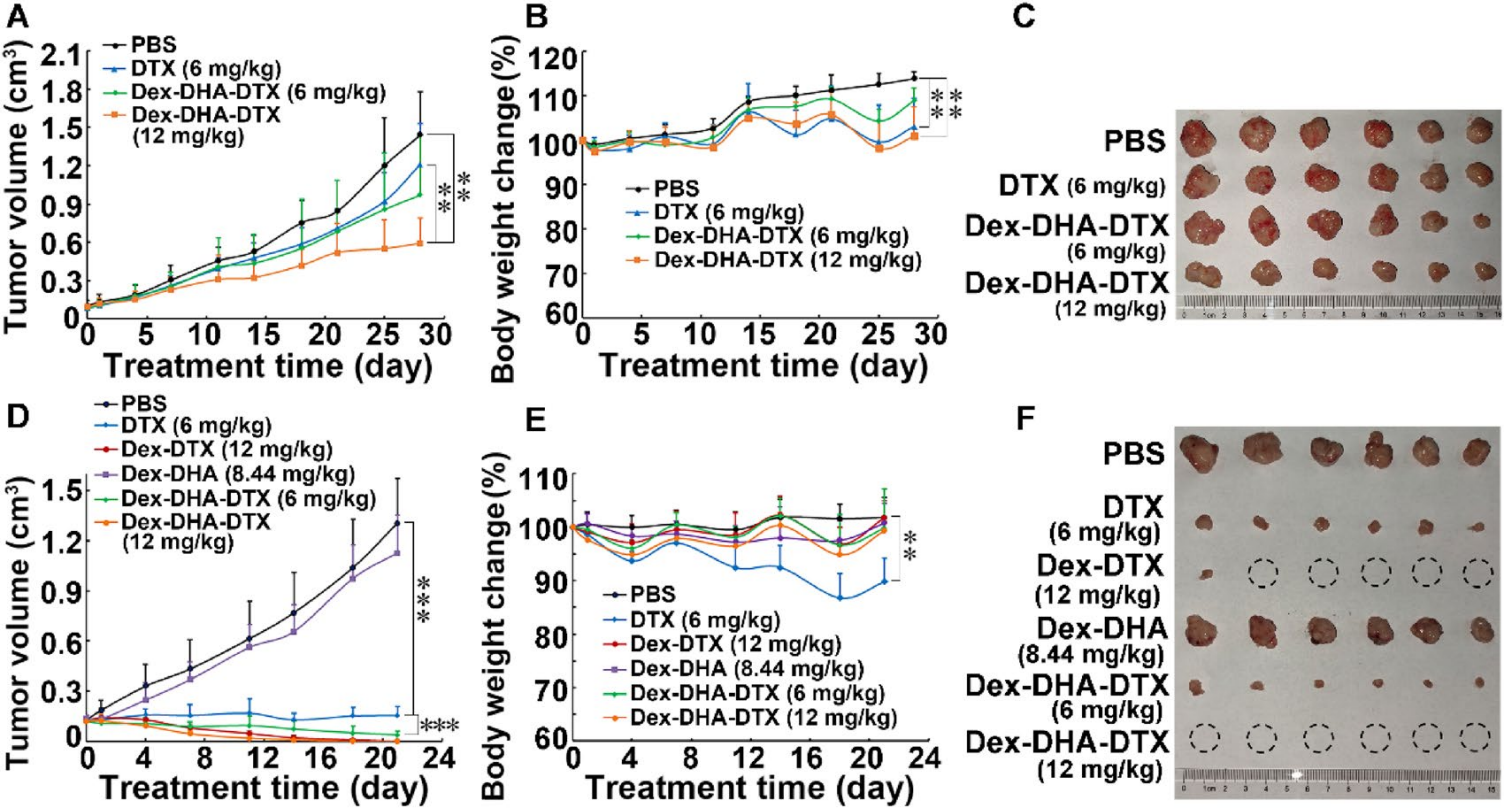
*In vivo* antitumor activities of the conjugate **18** (Dex–DHA–DTX) against tumor-bearing nude mice. (A and B) Mice tumor volume and mice body weight changes within 28 days in mice models bearing lung cancer cells H460. The mice were injected with the conjugate **18** via tail vein once a week for 4 weeks. (C) Tumor images of H460 xenograft at the end of the experiment. (D and E) Mice tumor volume and mice body weight changes within 21 days in MCF-7 xenograft models. The mice were injected with dextran-based conjugates once a week for 3 weeks. (F) Tumor images of MCF-7 xenograft at the end of the experiment. Data were presented as mean ± SD (*n* = 6). Dex means dextran. Red circle marked in tumor image represents complete eradication of xenograft tumor upon treatment. **P < 0.01, ***P < 0.001.

The *in vivo* antitumor efficacy of the conjugate **18** was evaluated first in mice bearing human lung cancer cells H460. Our preliminary experiment showed that twice intravenous injections of conventional DTX formulation at the dose of 12 mg/kg caused mice to lose more than 20% of their body weights. Thus, a dose of 6 mg/kg conventional DTX formulation was used in this study, while both doses of 6 mg/kg and 12 mg/kg conjugate **18** (equivalent to DTX) were intravenously injected into mice once a week for 4 weeks. The drug was given when the tumor sizes were reached around 100 mm^3^. At the end of the experiment, 6 mg/kg parent DTX treatment inhibited tumor growth by 16.2%, while the conjugate **18** treatment at the dose of 6 and 12 mg/kg inhibited tumor growth by 32.6% and 59.0%, respectively (Figure 6(A,C)). The tumor growth inhibitory efficacy of the conjugate **18** was similar to that of tandem type of dual-drug conjugate we reported previously (Dong et al., 2022). Our previous study showed that the treatment of 6 mg/kg of parent DTX, 6 mg/kg and 12 mg/kg of tandem type of dual-drug conjugate decreased the tumor volume by 19.6%, 36.8%, and 58.8% in H460 xenograft under the same schedule of administration as this study, respectively. Notably, 6 mg/kg of parent DTX resulted in more mice body weight loss compared to the 6 mg/kg conjugate **18** (Figure 6(B)). The side effects were further evaluated by histological examinations of the major tissues. H&E staining showed that all treatments had no obvious pathological abnormalities in kidney, spleen, lung, and liver (Figure S5, Supplementary data). All result showed that conjugate **18** possessed more antitumor activity and less toxicity than parent DTX in the lung cancer xenografted mice model.

The antitumor efficacy of the conjugate was also investigated in xenografted mice bearing MCF-7 cells. The mice were intravenously administrated with all the chemical agents once a week for 3 weeks. As compared with parent 6 mg/kg DTX treatment, 6 mg/kg dual-drug conjugate dextran–DHA–DTX **18** (equivalent to DTX) inhibited tumor growth greatly (*p* < .001, Figure 6(D)). Surprisingly, 12 mg/kg of conjugate **18** eradicated all the tumors on day 18 (Figure 6(F)). The antitumor effect of the conjugate **18** exceeded that of tandem type of dual-drug conjugate we reported previously, in which the treatment of tandem type of dual-drug conjugate with the dose of 12 mg/kg eliminated 4 out 6 xenograft tumors in MCF-7 bearing nude mice after four times of administration once a week (Dong et al., 2022). The single-drug conjugate dextran-DTX at the dose of 12 mg/kg eradicated 2 tumors on day 18 and 5 tumors on day 21 (Figure 6(F)). The dual-drug conjugate dextran–DHA–DTX **18** had obviously therapeutic efficacy than the single-drug conjugate dextran–DTX, illustrating that DHA enhanced the antitumor activity of DTX. The antitumor effect of another single-drug conjugate dextran-DHA was also evaluated. Dextran–DHA–DTX had the different loading capacity for DHA and DTX which were 19.21% and 13.51%, respectively. Dextran–DHA–DTX treatment at the dose of 12 mg/kg (equivalent to DTX) was theoretically equivalent to the combined treatment of 12 mg/kg DTX with 8.44 mg/kg DHA. Therefore, dextran–DHA at the dose of 8.44 mg/kg (equivalent to DHA) was chosen to evaluate the antitumor activity of DHA alone. The dextran–DHA at the dose of 8.44 mg/kg (equivalent to DHA) slightly inhibited tumor growth, however, the efficacy did not significantly differ from the parent DTX treatment (Figure 6(D,F)). Actually, optimization may be required to find a dose of DHA that inhibit tumor growth. Notably, 6 mg/kg parent DTX treatment led to 10.2% body weight reduction, as compared with the initial body weight on day 0. However, other dextran-based conjugates did not cause body weight loss and no significant difference in body weight was found compared to the control group (Figure 6(E)). All the results illustrated that conjugate **18** dramatically inhibited tumor growth without causing obvious side effects in mice model bearing MCF-7 cells.

## 4. Conclusion

In this study, we synthesized a dual-drug conjugate dextran–DHA–DTX **18** through independently covalently grafting DTX and DHA onto dextran with individual linkers. At the same time, glutamate was also separately attached to the dual-drug conjugate to provide negative charge for the conjugate. The conjugate **18** self-assembled into nanoparticles with the diameter of 102.3 ± 8.3 nm and demonstrated enhanced water solubility and improved pharmacokinetic parameters. More conjugate **18** entered cells than parent DTX when cells were incubated with conjugate and parent DTX at equivalent DTX concentrations. DHA has no influence on cellular uptake of parent DTX, while dextran–DHA enhanced the cytotoxicity of dextran–DTX *in vitro*. Notably, the conjugate **18** selectively accumulated in tumor tissues and dramatically reduced the DTX contents in normal tissues. The dual-drug conjugate **18** showed more superior antitumor activity than parent DTX and single-drug conjugates dextran-DTX and dextran-DHA. This strategy of producing polysaccharide dextran-based dual-drug conjugate is a very promising approach to obtain a new potent DTX formulation for clinical application.

## Supplementary Material

Supplemental Material
