# Supplementary material for: Polysaccharide dextran-based conjugate for selective co-delivery of two synergistic drugs docetaxel and docosahexaenoic acid to tumor cells
Source: Drug Deliv. 2022 Dec 1;30(1):40–50. doi: 10.1080/10717544.2022.2152133 (PMC9721411; doi:10.1080/10717544.2022.2152133)

**Supplementary data**

Polysaccharide dextran-based [conjugate for selective co-delivery of two synergistic drugs docetaxel and docosahexaenoic acid](https://pubmed.ncbi.nlm.nih.gov/34633394/) to tumor cells

Peng Dong^a^, Hongshuai Lv^a^, Weiping Jia^a^, Jiaojiao Liu^a^, Si Wang^b^, Xiaohai Li^b^, Jinghua Hu^b^, Ling Zhao^b^, Yikang Shi^a^

^a^National Glycoengineering Research Center, Shandong Key Laboratory of Carbohydrate Chemistry and Glycobiology, NMPA Key Laboratory for Quality Research and Evaluation of Carbohydrate Based Medicine, Shandong University, Qingdao 266237, China; ^b^Santolecan Pharmaceuticals LLC,1261 Islamorada Drive, Jupiter 33458, USA

**CONTACT** Yikang Shi, Eamil: [shiyikang@sdu.edu.cn](mailto:shiyikang@sdu.edu.cn) Address: National Glycoengineering Research Center, Shandong University, Qingdao, Shandong 266237, China

**Table of Contents**

1. Figures.

2. Synthetic routes and procedures for conjugates dextran-DHA-DTX, dextran-DTX, dextran-DHA, dextran-DHA-Cy7.5 and dextran-Cy7.5.

3. ^1^H NMR, ^13^C NMR and MS spectra of compounds 1 to 18.

1. **Figures**


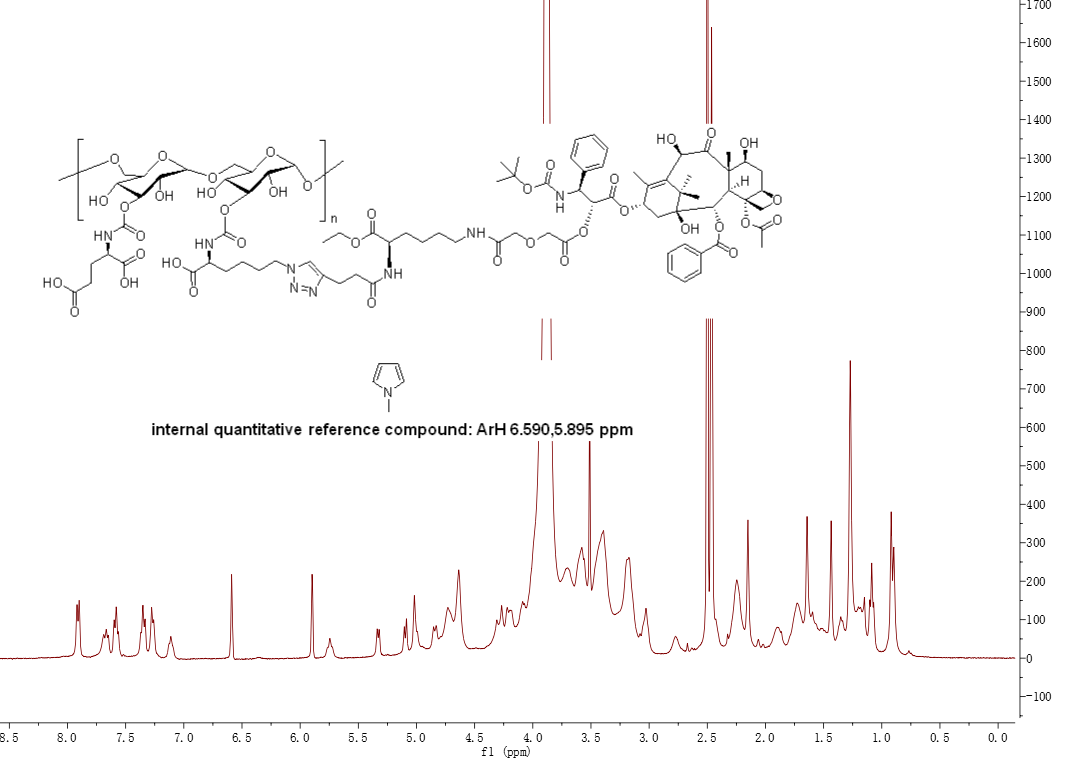


**Figure S1.** ^1^H NMR spectrum of compound **19** (Dextran-DTX).


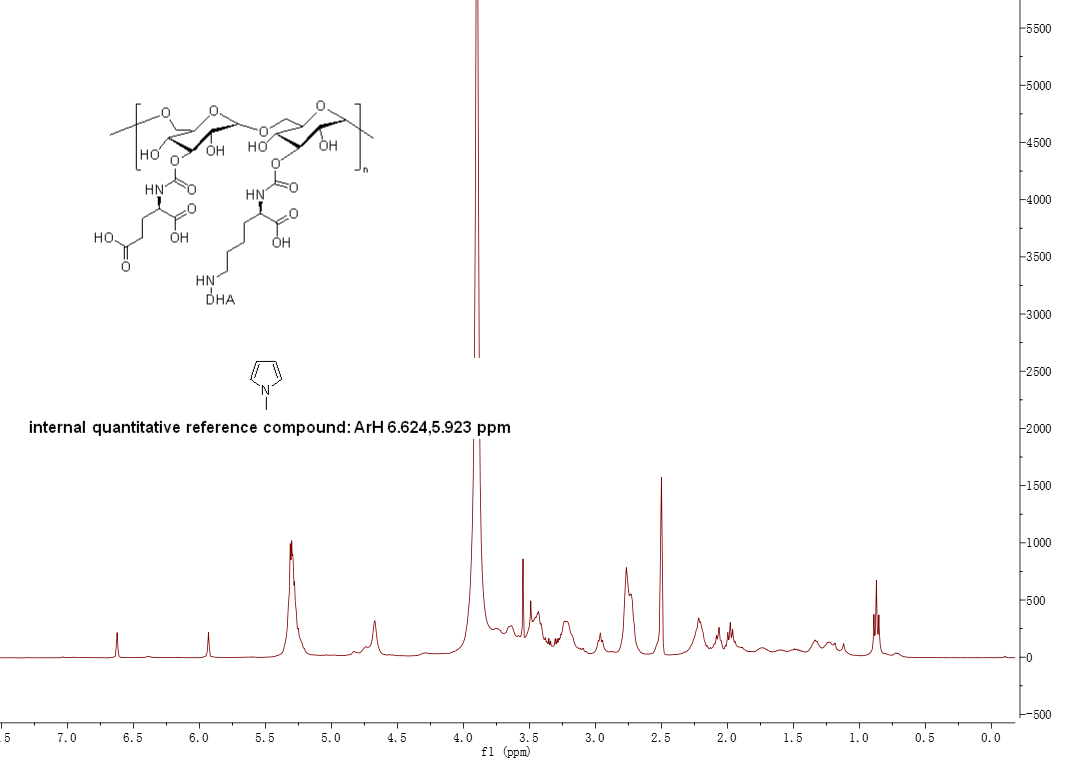
**Figure S2.** ^1^H NMR spectrum of compound **20** (Dextran-DHA).


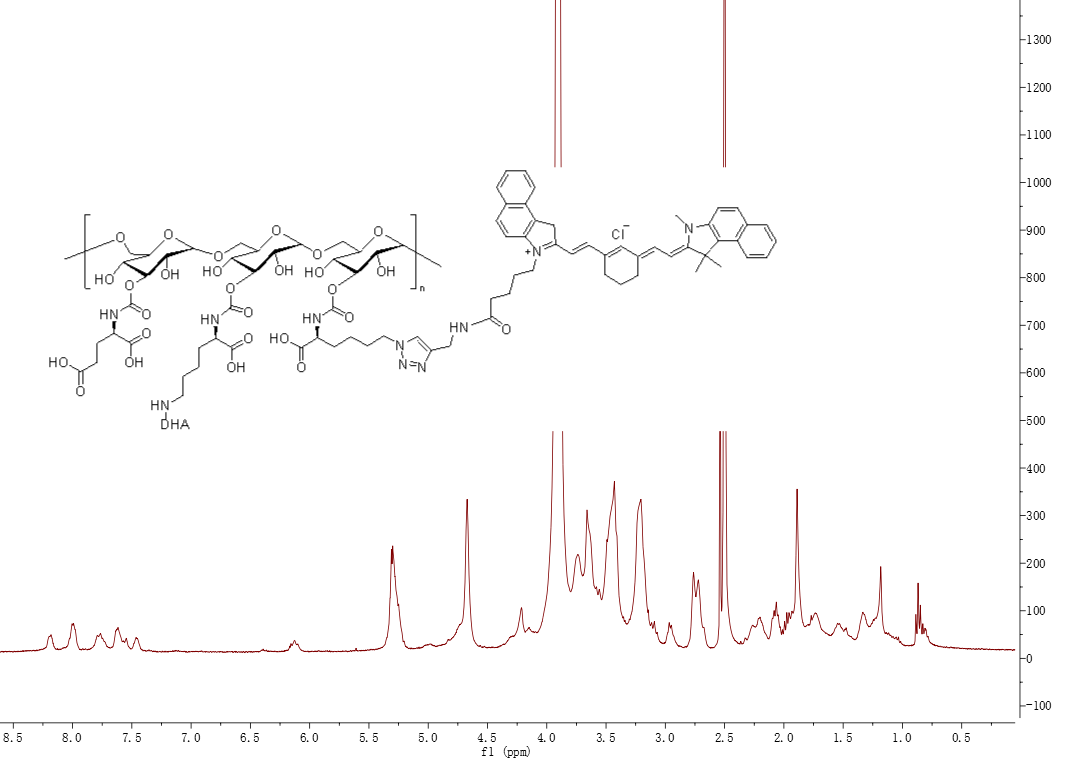


**Figure S3.** ^1^H NMR spectrum of compound **21** (Dextran-DHA-Cy7.5).


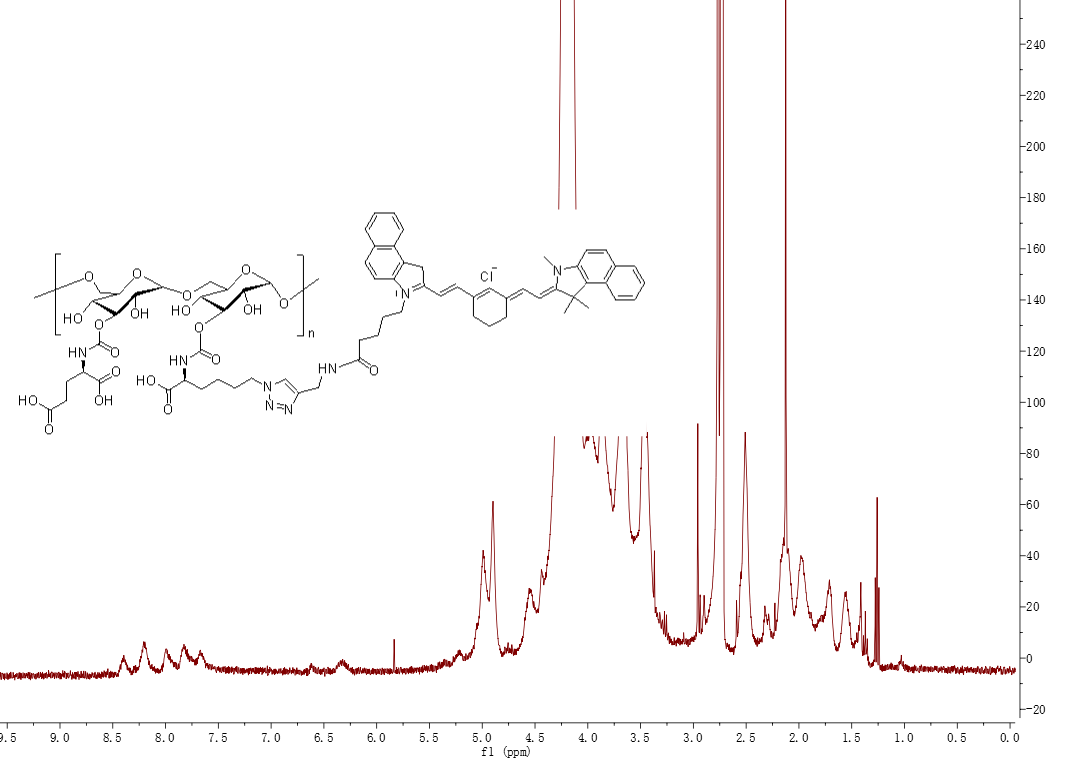


**Figure S4.** ^1^H NMR spectrum of compound **22** (Dextran-Cy7.5).


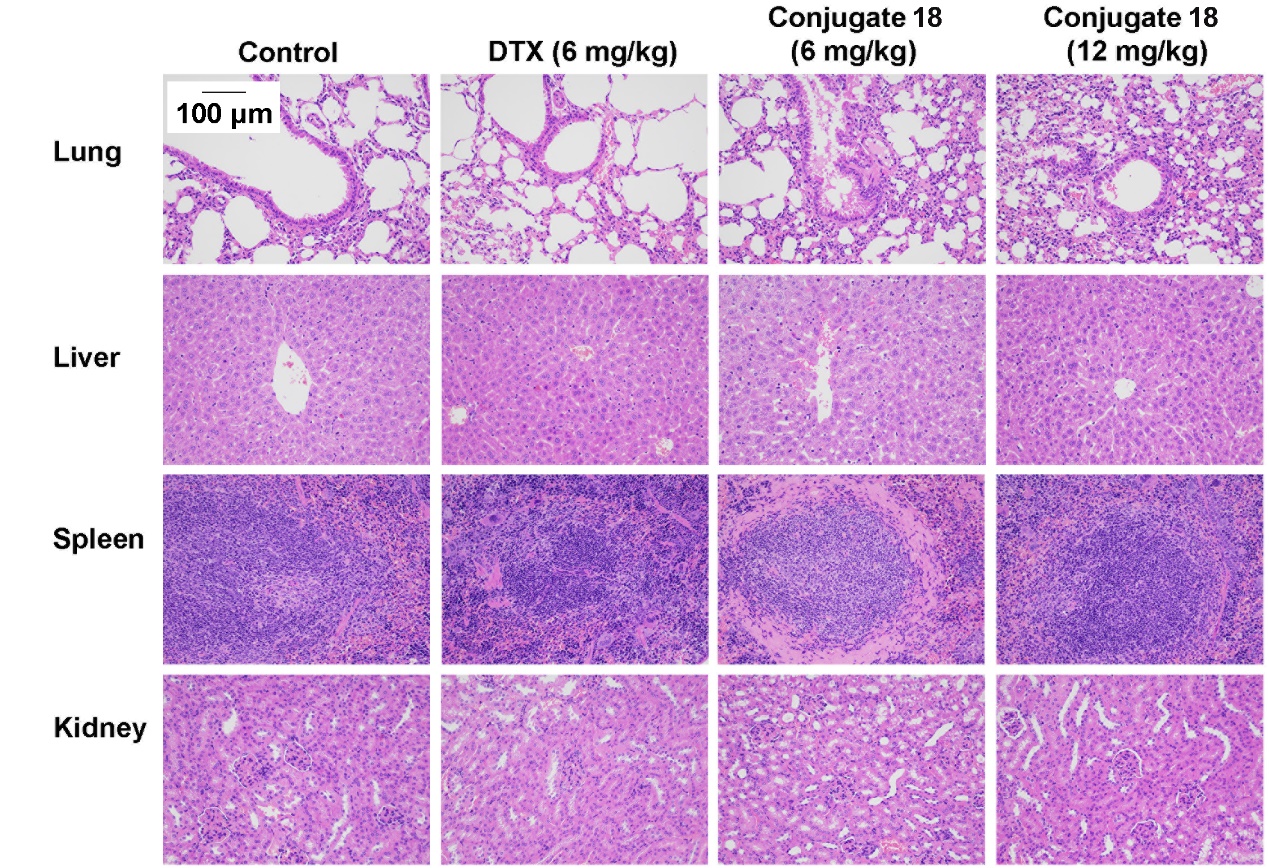


**Figure S5.** H&E staining of tumor and major organs of mice bearing 460 cells. Xenograft-bearing BALB/C nude mice were intravenously injected with conjugate 18 once a week for four weeks, and tissues were obtained after mice were sacrificed on day 28.

**2. Synthetic Routes and Procedures for Conjugates.**

**2.1. Preparation of functionalized dextran 8**

The synthetic route of functionalized dextran **8** was described in Scheme 1.

**2.1.1. Preparation of compound 1**

In a 250 mL round-bottom flask charged with DHA (1.0 g, 3.04 mmol), NHS (701 mg, 6.08 mmol) and EDCI (1.17 g, 6.08 mmol), 10 mL of anhydrous DCM was added and stirred at room temperature for 3 h. Upon completion of the reaction, the reaction mixture was partitioned between dichloromethane (100 mL) and brine (100 mL), and the organic phase washed with brine (100 mL×2), dried over anhydrous Na_2_SO_4_, filtered and evaporated to dryness. Next, 1.21 g (4.57 mmol) Boc-L-lysine was dissolved in distilled water in a 250 mL round-bottom flask, followed by addition of 1.29 g (3.04 mmol) of the above freshly prepared DHA NHS ester in dry THF and stirred at room temperature for 3 h. Upon completion of the reaction, the reaction mixture was partitioned between dichloromethane (100 mL) and brine (100 mL), and the organic phase was further washed with brine twice (50 mL×2), dried over anhydrous Na_2_SO_4_, filtered, concentrated and purified on a silica gel column eluted with methanol in chloroform (0 - 10%) to provide 1.62 g of compound **1**. Yield: 91%.

^1^H NMR (400 MHz, CDCl_3_): δ 5.84 (s, 1H), 5.37 (m, 12H), 4.28 (s, 1H), 3.25 (d, *J* = 4.0 Hz, 2H), 2.83 (m, 10H), 2.41 (m, 2H), 2.25 (t, *J* = 7.2 Hz, 2H), 2.07 (m, 2H), 1.72 (m, 1H), 1.53 (m, 1H), 1.44 (s, 9H), 1.32 – 1.23 (m, 3H), 0.97 (t, *J* = 7.6 Hz, 3H), 0.86 (m, 1H).

^13^C NMR (100 MHz, CDCl_3_): δ 174.37, 172.21, 154.83, 131.02, 128.38, 127.56, 127.29, 127.27, 127.25, 127.10, 127.08, 127.05, 127.02, 126.85, 125.99, 79.04, 52.11, 38.24, 35.37, 31.08, 30.90, 28.68, 27.92, 27.33, 24.62, 24.61, 24.59, 24.53, 22.41, 21.39, 19.54, 13.26.

MS (ESI, *m/z*): calcd for C_33_H_51_N_2_O_5_ [M-H]^-^:555.3803; found: 555.3809.

**2.1.2. Preparation of compound 2**

In a 250 mL round-bottom flask charged with compound **1** (2.74 g, 3.70 mmol), DMAP (1.54 g,12.6 mmol) and EDCI (2.42 g,12.6 mmol),10 mL anhydrous DMF and 30 mL anhydrous ethyl alcohol were added and stirred at room temperature for 3 h. Upon completion of the reaction, the reaction mixture was partitioned between ethyl acetate (100 mL) and brine (100 mL), and the organic phase washed with brine (100 mL×2), dried over anhydrous Na_2_SO_4_, filtered, concentrated and purified on a silica gel column eluted with ethyl acetate in petroleum (5 - 60%) to provide 1.41 g of compound **2**. Yield:41%.

^1^H NMR (400 MHz, CDCl_3_): δ 5.59 (s, 1H), 5.37 (m, 12H), 5.08 (d, *J* = 7.6 Hz, 1H), 4.20 (m, 3H), 3.23 (q, *J* = 6.8 Hz, 2H), 2.82 (m, 10H), 2.40 (q, *J* = 7.2 Hz, 2H), 2.20 (t, *J* = 7.2 Hz, 2H), 2.07 (m, 2H), 1.76 (s, 1H), 1.63 (m, 1H), 1.52 (m, 2H), 1.43 (s, 9H), 1.36 (m, 2H), 1.27 (t, *J* = 7.2 Hz, 3H), 0.96 (t, *J* = 7.6 Hz, 3H).

^13^C NMR (100 MHz, CDCl_3_): δ 172.71, 172.32, 132.04, 129.25, 128.57, 128.31, 128.28, 128.25, 128.11, 128.08, 127.87, 127.01, 79.85, 61.33, 53.21, 39.14, 36.48, 32.54, 29.05, 28.33, 25.65, 25.63, 25.55, 23.44, 22.61, 20.56, 14.27, 14.20.

MS (ESI, *m/z*): calcd for C_35_H_56_N_2_NaO_5_ [M+Na]^+^:607.4081; found: 607.4071.

**2.1.3. Preparation of compound 3**

In a 250 mL round-bottom flask, 0.66 g of compound **2** (1.13 mmol) was dissolved in 10.0 mL of hydrochloride ethanol solution (4.0N), and stirred at room temperature for 1 h. The reaction mixture was concentrated and purified on on a silica gel column eluted with methanol in chloroform (0 - 8%) to provide 0.98 g of compound **2**. Yield: 100%.

^1^H NMR (400 MHz, CDCl_3_): δ 5.61 (s, 1H), 5.36 (m, 12H), 4.16 (q, *J* = 6.8 Hz, 2H), 3.24 (q, *J* = 6.8 Hz, 2H), 2.82 (m, 10H), 2.40 (q, *J* = 7.2 Hz, 2H), 2.20 (t, *J* = 7.6 Hz, 2H), 2.06 (m, 2H), 1.85 - 1.68 (m, 4H), 1.52 (m, 3H), 1.41 (m, 2H), 1.26 (t, J = 7.2 Hz, 3H), 0.96 (t, J = 7.6 Hz, 3H).

^13^C NMR (100 MHz, CDCl_3_): δ 174.85, 171.25, 131.02, 128.24, 127.56, 127.30, 127.26, 127.24, 127.09, 127.06, 126.85, 125.99, 59.91, 53.25, 38.23, 35.49, 33.24, 28.29, 24.63, 24.61, 24.53, 22.44, 21.96, 19.55, 13.26, 13.24.

MS (ESI, *m/z*): calcd for C_30_H_49_N_2_O_3_ [M+H]^+^:485.3738; found: 485.3752.

**2.1.4. Preparation of compound 4**

In a 250 mL round bottom flask charged with compound **3** (0.98 g, 2.03 mmol), 50 mL of anhydrous DCM and 0.82 mL of pyridine (0.80 g, 26.9 mmol) were added under nitrogen protection and cooled down to 0^o^C, followed by slow addition of diphosgene (1.63 g, 2.44 mmol) in anhydrous DCM (30 mL) and stirred for 3h. The reaction mixture was diluted with DCM (100 mL) and washed with 1.0 N HCl solution three times (50 mL×3), dried over anhydrous Na_2_SO_4_, filtered, and evaporated to dryness to offer compound **4** (0.55 g)**.** Yield: 53%. This compound was directly used in next step without purification.

^1^H NMR (400 MHz, CDCl_3_): δ 5.36 (m, 12H), 4.40 - 4.00 (m, 4H), 3.67 (s, 1H), 3.30 - 3.20 (m, 2H), 2.83 (m, 10H), 2.41 (m, 3H), 2.22 (t, *J* = 7.2 Hz, 1H), 2.07 (m, 3H), 1.73 - 1.57 (m, 2H), 1.37 - 1.26 (m, 1H), 1.32 - 1.24 (m, 4H), 0.96 (t, *J* = 7.6 Hz, 3H).

^13^C NMR (100 MHz, CDCl_3_): δ 171.50, 168.48, 131.02, 128.32, 128.28, 127.56, 127.29, 127.27, 127.24, 127.09, 127.05, 127.02, 126.85, 125.99, 76.32, 76.01, 75.69, 61.71, 60.48, 52.54, 52.01, 51.33, 38.07, 35.42, 34.63, 31.36, 28.55, 27.91, 27.12, 24.63, 24.61, 24.53, 22.68, 22.43, 21.39, 20.93, 19.55, 13.53, 13.26, 13.17, 13.07.

MS (ESI, *m/z*): calcd for C_31_H_46_N_2_O_4_ [M+H]^+^:511.35; found: 511.74.

**2.1.5. Preparation of compound 5**

In a 250 mL round-bottom flask charged with Boc-Lys(N_3_)-OH (1.5 g, 5.50 mmol), HOBt (1.27 g, 8.26 mmol) and HBTU (3.13 g, 8.26 mmol), 10 mL of anhydrous DMF was added and stirred at room temperature for 30 min; and then 1.53 mL of triethylamine (1.11 g, 11.02 mmol) was dropwise added. After stirred for 30 minutes, 20.0 mL of anhydrous ethanol was added. The reaction was continuously stirred at room temperature overnight. Upon completion of the reaction, the reaction mixture was partitioned between ethyl acetate (100 mL) and brine (100 mL), and the organic phase washed with brine (100 mL×2), dried over anhydrous Na_2_SO_4_, filtered, concentrated and purified on a silica gel column eluted with ethyl acetate in petroleum (5 - 60%) to provide 1.13 g of compound **5**. Yield: 59%.

^1^H NMR (600 MHz, CDCl_3_): δ 5.03 (d, *J* = 6.0 Hz, 1H), 4.31 (m,1H), 4.20 (m,2H), 3.29 (t, *J* = 6.8 Hz, 2H), 1.83 (m,1H), 1.72 - 1.56 (m, 4H), 1.47 (s, 10H), 1.28 (t, *J* = 6.6 Hz, 3H).

^13^C NMR (150 MHz, CDCl_3_): δ 173.62, 156.34, 80.88, 78.20, 78.15, 62.35, 54.23, 52.15, 33.39, 29.43, 29.30, 23.48, 15.17.

ESI-MS (*m/z*): calcd for C_13_H_25_N_4_O_4_ [M+H]^+^: 301.1870; found: 301.1879.

**2.1.6. Preparation of compound 6**

In a 250 mL round-bottom flask, 1.13 g of compound **1** (3.76 mmol) was dissolved in 10.0 mL of hydrochloride ethanol solution (4.0N), and stirred at room temperature for 1 h. The reaction mixture was concentrated and purified on on a silica gel column eluted with methanol in chloroform (0 - 8%) to provide 1.16 g of compound **2**. Yield: 100%.

^1^H NMR (600 MHz, CDCl_3_): δ 4.20 (m, 2H), 3.50(t, *J* = 6.0 Hz, 1H), 3.29 (t, *J* = 7.2 Hz, 2H), 1.78 (m, 1H), 1.67 - 1.58 (m, 3H), 1.51 - 1.45 (m, 2H), 1.27 (t, *J* = 7.2 Hz, 3H).

^13^CNMR (150 MHz, CDCl_3_): δ 175.12, 61.30, 53.08, 54.22, 51.30, 33.89, 28.72, 22.95, 14.36.

ESI-MS (*m/z*): calcd for C_8_H_17_N_4_O_2_ [M+H]^+^: 201.1346; found: 201.1349.

**2.1.7. Preparation of compound 7**

In a 250 mL round bottom flask, compound **6** (1.16 g, 5.80 mmol) was dissolved in 50 mL of anhydrous DCM and 2.33 mL of pyridine (2.29 g, 29.0 mmol) under nitrogen protection and cooled down to 0^o^C, followed by slow addition of diphosgene (1.38 g, 6.96 mmol) in anhydrous DCM (30 mL) and stirred for 3h. The reaction mixture was diluted with DCM (100 mL) and washed with 1.0 N HCl solution three times (50 mL×3), dried over anhydrous Na_2_SO_4_, filtered, and evaporated to dryness to offer compound **7** (0.78 g)**.** Yield: 60%. This compound was directly used in next step without purification.

^1^H NMR (400 MHz, CDCl_3_) 4.26 (q, *J* = 7.2 Hz, 2H), 4.03 (dd, *J* = 4.4 Hz, 1H), 3.31 (t, *J* = 6.8 Hz, 2H), 1.92 - 1.69 (m, 2H), 1.64 - 1.57 (m, 2H), 1.53 - 1.44 (m, 2H), 1.31 (t, *J* = 6.8 Hz, 3H), 1.24 (s, 1H).

^13^C NMR (100 MHz, CDCl_3_): δ 172.11, 127.89, 63.54, 58.23, 52.04, 34.26, 30.66, 29.20, 23.72, 15.11.

MS (ESI, *m/z*): calcd for C_9_H_18_N_5_O_3_ [M+NH_4_]^+^:244.14; found: 244.69.

**2.1.8. Preparation of compound 8**

2.0 g of dextran with average molecular weight of 100k daltons was totally dried in oil bath at 60^o^C under high vacuum for 10 h, and then dissolved in 10 mL of anhydrous DMSO at 60^o^C. After cooled down to room temperature, the oil bath was removed. To the above solution, dimethyl (S)-2-isocyanatopentanedioate (3.81 g, 15.2 mmol) , compound **4** (0.55 g, 1.07 mmol), compound **7** (0.79 g, 3.52 mmol) and DMAP (6.55 g, 53.6 mmol) were slowly added and stirred at room temperature overnight. After the completion of the reaction, the reaction mixture was directly dialyzed against distilled water for 24 h, then concentrated and hydrolyzed with NaOH (3.16 g, 79.2 mmol) for 5 h. The resulting solution was adjusted to pH 3.0 - 5.0 with 4 N HCl solution, and continued to dialyzed against distilled water three times, concentrated, and lyophilized to provide functionalized dextran **8** (3.08 g). Yield: 60%.

^1^H NMR (selected characteristic signals, 400 MHz, DMSO-d_6_, ppm): major signals: 4.51 - 5.26 (m, CHOH), 3.26 - 4.24 (m, CHOH, CH_2_OH); minior signals: 1.51 -2.02 (m, CH_2_).

**2.2. Synthetic scheme 2: Preparation of DTX-Linker 17**

The synthetic route of compound **17** was described in Scheme 2.

**2.2.1. Preparation of compound 9**

To a 250 mL round-bottom flask charged with 5.0 g (6.1 mmol) of docetaxel and 2.5 g (37.1 mmol) of imidazole in 15.0 mL of anhydrous DMF,2.76 g (18.3 mmol) of TBDMSCl was added. The reaction mixture was stirred at room temperature for 12h. Upon completion of the reaction, the reaction mixture was partitioned between ethyl acetate (200 mL) and brine (200 mL), and the organic phase was further washed with brine (100 mL×2), dried over anhydrous Na_2_SO_4_, filtered, concentrated and purified on a silica gel column eluted with ethyl acetate in petroleum (10 - 40%) to provide 4.50 g of compound **9**. Yield: 80%.

^1^H NMR (400 MHz, CDCl_3_): δ 8.12 (d, *J* = 7.2 Hz, 2H), 7.59 (t, *J* = 7.6 Hz, 1H), 7.49 (t, *J* = 8.0 Hz, 2H), 7.37 (t, *J* = 7.6 Hz, 2H), 7.32 - 7.24 (m, 3H), 6.34 (t, *J* = 9.6 Hz, 1H), 5.70 (d, *J* = 7.2 Hz, 1H), 5.43 (d, *J* = 9.6 Hz, 1H), 5.31 (d, *J* = 8.0 Hz, 1H), 4.98 (d, *J* = 8.4 Hz, 1H), 4.52 (s, 1H), 4.35 - 4.2 (m, 3H), 4.12 (q, *J* = 7.2 Hz, 1H), 3.96 (d, *J* = 7.2 Hz, 1H), 2.70 - 2.49 (m, 4H), 2.37 (dd, *J* = 16.8, 9.6 Hz, 1H), 2.15 (dd, *J* = 13.6, 4.8 Hz, 1H), 1.96 - 1.81 (m, 4H), 1.79 - 1.59 (m, 5H), 1.34 - 1.20 (m, 13H), 1.12(s, 1H),0.74 (s, 9H), -0.10 (s, 3H), -0.30 (s, 3H).

^13^C NMR (100 MHz, CDCl_3_): δ 211.59, 171.33, 170.15, 167.10, 155.17, 139.12, 135.51, 133.61, 130.21, 129.24, 128.73, 128.57, 127.68, 126.45, 84.19, 81.05, 79.92, 79.10, 75.71, 75.08, 74.46, 71.95, 71.25, 60.39, 57.56, 46.37, 43.16, 36.97, 35.88, 28.16, 26.38, 25.47, 22.94, 21.16, 21.02, 18.18, 14.28, 14.19, 9.98, -5.37, -5.93.

ESI-MS (*m/z*): calcd for C_49_H_68_NO_14_Si [M+H]^+^: 922.44; found: 922.03.

**2.2.2. Preparation of compound 10**

To a 250 mL round-bottom flask charged with 4.50 g (4.88 mmol) of compound **9** and 3.48 g (28.5 mmol) of DMAP, 15 mL of absolute THF was added under nitrogen protection and cooled down to 0^o^C, followed by addition of 3.0 mL (28.2 mmol)of AllocCl. After stirred for another 10 minutes, the cooled batch was removed and the reaction mixture allowed to warm up to room temperature and stirred for another 12h. Upon completion of the reaction, the reaction mixture was partitioned between dichloromethane (200mL) and saturated sodium citrate solution (200 mL), and the

organic phase was further washed with saturated sodium citrate solution (100 mL×2), dried over anhydrous Na_2_SO_4_, filtered, concentrated and purified on a silica gel column eluted with ethyl acetate in petroleum (10 - 50%) to provide 4.27 g of compound **10**. Yield: 79%.

^1^H NMR (400 MHz, CDCl_3_): δ 8.12 (d, *J* = 7.2 Hz, 2H), 7.59 (t, *J* = 7.6 Hz, 1H), 7.49 (t, *J* = 8.0 Hz, 2H), 7.37 (t, *J* = 7.6 Hz, 2H), 7.32 - 7.24 (m, 3H), 6.35 - 6.20 (m, 2H), 6.05 - 5.90 (m,2H), 5.71 (d, *J* = 8.4 Hz, 1H), 5.52 (dd, *J* = 10.8, 7.2 Hz, 1H), 5.45 - 5.20 (m, 6H), 4.98 (d, *J* = 7.6 Hz, 1H), 4.65 (m, 4H), 4.47 (d, *J* = 2.5 Hz, 1H), 4.20 (d, *J* = 8.0 Hz, 1H), 4.12 (q, *J* = 7.2 Hz, 1H), 3.97 (d, *J* = 7.6 Hz, 1H), 2.68 - 2.53 (m,4H), 2.39 (dd, *J* = 15.2, 9.6 Hz, 1H), 2.21 (dd, *J* = 16.0, 8.8 Hz, 1H), 2.08 - 1.95 (m, 5H), 1.84 (s, 2H), 1.64(s, 2H), 1.37 - 1.23(m, 13H) 1.19 (s, 3H), 0.74 (s, 9H), 0.07 (s, 3H), -0.31 (s, 3H).

^13^C NMR (100 MHz, CDCl_3_): δ 201.63, 171.45, 171.10, 169.96, 166.97, 155.23, 154.00, 153.89, 142.36, 133.67, 132.18, 131.89, 131.51, 130.19, 129.12, 128.72, 128.58, 127.70, 126.43, 119.13, 118.64, 83.93, 80.86, 79.98, 78.92, 78.22, 76.38, 75.58, 75.25, 74.55, 71.22, 69.07, 68.88, 60.37, 56.09, 46.87, 43.24, 35.32, 33.39, 28.18, 26.25, 25.47, 22.91, 21.44, 21.03, 18.16, 14.65, 14.20, 10.73, -5.34, -5.92.

ESI-MS (*m/z*): calcd for C_57_H_79_N_2_O_18_Si [M+NH_4_]^+^: 1107.51; found: 1107.91.

**2.2.3. Preparation of compound 11**

In a 250 mL round-bottom flask, 4.27 g (3.85 mmol) of compound **10** was dissolved in 15.0 mL of THF, and 2.5 mL of tetrabutylammonium fluoride solution (TBAF, 1.0 M) was added and stirred for 30 minutes at room temperature. Upon completion of the reaction, the reaction mixture was concentrated and purified on a silica gel column eluted with ethyl acetate in petroleum (10 - 40%) to provide 3.06 g of compound **11**. Yield: 80%.

^1^H NMR (400 MHz, CDCl_3_): δ 8.1 (d, *J* = 8.0 Hz, 2H), 7.61 (t, *J* = 6.8 Hz, 1H), 7.49 (t, *J* = 7.6 Hz, 2H), 7.43 - 7.28 (m,5H), 6.25 - 6.14 (m, 2H), 6.04 - 5.91 (m,2H), 5.53 - 5.35(m,3H), 5.34 - 5.22 (m, 3H), 4.72 - 4.60 (m, 4H), 4.32 (d, *J* = 8.4 Hz, 1H), 4.22 - 4.07 (m, 3H), 3.92 (d, *J* = 6.8 Hz, 1H), 2.62 (m,1H), 2.45 - 2.25 (m, 4H), 2.10 - 2.02(m,3H) 1.99 - 1.92 (m, 3H), 1.83 (s, 3H), 1.36 (s, 9H), 1.30 - 1.23 (m, 6H), 1.20(s, 3H).

^13^C NMR (100 MHz, CDCl_3_): δ 201.56, 171.12, 170.21, 166.93, 153.98, 153.85, 141.69, 138.46, 133.75, 132.62, 131.86, 131.49, 130.17, 129.09, 128.85, 128.71, 128.08, 126.80, 119.20, 118.67, 83.84, 80.92, 80.23, 78.70, 78.25, 76.42, 75.28, 74.34, 73.59, 72.32, 69.09, 68.92, 60.38, 56.23, 46.92, 43.17, 35.34, 33.42, 28.21, 26.37, 22.52, 21.02, 14.67, 14.19, 10.65.

ESI-MS (*m/z*): calcd for C_51_H_65_N_2_O_18_ [M+NH_4_]^+^: 993.42; found: 993.11.

**2.2.4. Preparation of compound 12**

To a 250 ml round-bottom flask charged with 4-pentynoic acid (1.0 g ,10.2 mmol), N-hydroxysuccinimide (1.76 g, 15.3 mmol) and EDCI (2.93 g, 15.3 mmol) were dissolved in 100 mL of anhydrous DCM and stirred for 3 h at room temperature. Upon completion of the reaction, the reaction mixture was washed with brine (100 mL×2), dried over anhydrous Na2SO4, filtered and evaporated to dryness. Next, 3.77 g (15.3 mmol) of NƐ-Boc-L-lysine and 2.84 mL of Et3N (20.4 mmol) were combined in 100 mL of acetonitril/water (4:1), followed by addition of 1.98 g (10.2 mmol) of the above freshly prepared 4-pentynoic acid NHS ester in dry THF and stirred at room temperature overnight. Upon completion of the reaction, the reaction mixture was evaporeated and the residue was partitioned between dichloromethane (200 mL) and brine (200 mL), and the organic phase was further washed with brine twice (100 mL×2), dried over anhydrous Na2SO4, filtered, concentrated and purified on a silica gel column eluted with methanol in chloroform (0 - 10%) to provide 2.75 g of compound 12. Yield:83%.

^1^H NMR (400 MHz, CDCl_3_) δ 9.68 (s, 1H), 6.92 (d, *J* = 6.4 Hz, 1H), 4.62 (m, 1H), 3.08 (m, 2H), 2.50 (m, 4H), 2.02 (m, 1H), 1.89 (m, 1H), 1.77 (m, 1H), 1.49 (m, 3H), 1.44 (s, 9H), 1.40 (s, 2H).

^13^C NMR (100 MHz, CDCl3) δ 173.88, 170.74, 81.75, 68.54, 51.24, 39.17, 34.04, 30.45, 28.41, 27.41, 21.17, 13.85.

ESI-MS (*m/z*): calcd for C_16_H_27_N_2_O_5_ [M+H]^+^: 327.1914; found: 327.1918.

**2.2.5. Preparation of compound 13**

In a 250 mL round-bottom flask, compound **12** (2.75 g, 8.43 mmol), DMAP (1.54 g, 12.6 mmol) and EDCI (2.42 g, 12.6 mmol) were combined in 50 mL of anhydrous DMF, and 30 mL anhydrous ethanol was added and stirred overnight. After removal of volatiles, the residue was partitioned between ethyl acetate (100 mL) and brine (100 mL), and the organic phase was further washed with brine twice (100 mL×2), dried over anhydrous Na_2_SO_4_, filtered, concentrated and purified on a silica gel column eluted with ethyl acetate in petroleum (10 - 30%) to provide 2.60 g of compound **13**. Yield: 87%.

^1^H NMR (400 MHz, CDCl_3_) δ 6.35 (d, *J* = 7.6 Hz, 1H), 4.58 (m, 1H), 4.13 (q, *J* = 5.2 Hz, 2H), 3.07 (t, *J* = 6.4 Hz, 2H), 2.48 (m, 4H), 2.02 (m, 1H), 1.85 (m, 1H), 1.47 (m, 2H), 1.42 (s, 9H), 1.33 - 1,23 (m, 5H).

^13^C NMR (100 MHz, CDCl_3_) δ 171.38, 169.76, 155.11, 81.81, 78.25, 68.41, 60.48, 51.00, 39.19, 34.17, 31.06, 28.52, 27.40, 21.26, 13.80, 13.16.

ESI-MS (m/z): calcd for C18H31N2O5 [M+H]+: 355.2227; found: 355.2239.

**2.2.6. Preparation of compound 14**

To a 250ml round-bottom flask charged with compound **13** (1.41 g ,3.98 mmol) 50 mL of anhydrous DCM and 3 mL of THF was added and stirred for 1 h at room temperature. Upon completion of the reaction, the reaction mixture was concentrated and purified on a reversed phase column eluted with water in methanol (5 - 95%) to provide 0.47 g of compound **18**. Yield: 46%.

^1^H NMR (400 MHz, DMSO-*d*_6_) δ 8.31 (d, *J* = 7.6 Hz, 1H), 4.19 (m, 1H), 4.07 (m, 1H), 3.08 (q, *J* = 7.2 Hz, 2H), 2.80 - 2.70 (m, 2H), 2.38 - 2.28 (m, 4H), 1.75 - 1.44 (m, 4H), 1.40 - 1.27 (m, 2H), 1.17 (m, 5H).

^13^C NMR (100 MHz, DMSO-*d*_6_) δ 172.49, 171.14, 84.06, 71.75, 60.94, 52.22, 46.14, 34.28, 30.81, 26.93, 22.71, 14.60, 14.50, 8.99.

ESI-MS (*m/z*): calcd for C_13_H_23_N_2_O_3_ [M+H]^+^: 255.1703; found: 255.1705.

**2.2.7. Preparation of compound 15**

To a 250 mL round-bottom flask charged with 0.47 g (1.84 mmol) of compound **18** in 20 mL of anhydrous DMF, 1.28 mL (9.19 mmol) of triethylamine was added and stirred at room temperature overnight. Upon completion of the reaction, the reaction mixture was concentrated and purified on a reversed phase column eluted with water in methanol (0- 100%) to provide 0.54 g of compound **19**. Yield: 80%.

^1^H NMR (400 MHz, CD_3_OD) δ 4.37 (m, 1H), 4.22 (m, 3H), 4.14 - 4.13 (m, 2H), 3.73 (m, 1H), 3.26 (t, *J* = 7.2 Hz, 2H), 2.46 (m, 4H), 2.23 (s, 1H), 1.34 (m, 1H), 1.71 (m, 1H), 1.62 - 1.50 (m, 2H), 1.49 - 1.38 (m, 2H), 1.26 (t, *J* = 7.2 Hz, 3H), 1.15 (m, 1H).

^13^C NMR (100 MHz, DMSO-*d_6_*) δ 172.20, 171.52, 170.74, 168.85, 83.69, 71.25, 70.24, 68.01, 60.48, 52.05, 37.95, 33.93, 30.68, 28.68, 22.73, 14.24, 14.11.

ESI-MS (*m/z*): calcd for C_17_H_27_N_2_O_7_ [M+H]^+^: 371.1813; found: 371.1812.

**2.2.8. Preparation of compound 16**

In a 250 mL round-bottom flask, compound **15** (1.18 g，3.19 mmol), DMAP (0.39 g，3.19 mmol) and EDCI (0.61 g，3.19 mmol) were combined in 10.0 mL of anhydrous THF, followed by addition of compound **11** (1.55 g, 1.59 mmol) and stirred at room temperature for 3 h. The reaction mixture was concentrated and purified on a silica gel column eluted with methanol in methylene chloride (1.0 - 5.0 %) to provide 1.41 g of compound **16**. Yield: 67%.

^1^H NMR (400 MHz,CDCl_3_) δ 8.1 (d, *J* = 7.6 Hz, 2H), 7.61 (t, *J* = 7.2 Hz, 1H), 7.50 (t, *J* = 7.6 Hz, 2H), 7.41 (t, *J* = 7.6 Hz, 2H), 7.36 - 7.26 (m, 3H), 6.75 (m, 1H), 6.23 (s, 1H), 6.02 - 5.89 (m, 2H), 5.69 (d, *J* = 6.8 Hz, 1H), 5.49 (m, 4H), 5.43 - 5.19 (m, 6H), 4.97 (m, 1H), 4.72 - 4.60 (m, 4H), 4.59 - 4.53 (m, 1H), 4.36 - 4.23 (m, 2H), 4.24 - 4.09 (m, 4H), 4.00 - 3.90 (m, 3H), 3.34 - 3.24 (m, 2H), 2.62 (m, 1H), 2.56 - 2.48 (m, 2H), 2.47 - 2.40 (m, 5H), 2.05 - 1.98 (m, 5H), 1.85 (m, 3H), 1.76 (m.3H), 1.55 (m, 2H), 1.34 (m, 9H), 1.31 - 1.21 (m, 6H), 1.18 (s, 3H).

^13^C NMR (100 MHz, CDCl_3_) δ 200.59, 171.35, 169.90, 169.81, 168.66, 167.84, 167.60, 166.69, 165.93, 154.11, 152.95, 152.82, 141.07, 135.87, 132.69, 131.43, 130.89, 130.51, 129.17, 128.17, 128.05, 128.02, 127.69, 127.45, 125.25, 118.12, 117.62, 82.87, 81.95, 79.87, 79.61, 77.81, 74.26, 73.89, 73.51, 71.08, 70.13, 68.48, 68.41, 68.08, 67.88, 67.06, 60.43, 55.10, 51.07, 45.92, 42.20, 37.34, 34.14, 32.35, 30.94, 30.82, 28.02, 27.16, 25.28, 21.62, 21.33, 20.25, 13.84, 13.80, 13.56, 13.16, 9.70.

ESI-MS (*m/z*): calcd for C_68_H_85_KN_3_O_24_ [M+K]^+^: 1366.5155; found: 1366.5166.

**2.2.9. Preparation of compound 17**

Compound **16** (141 mg，1.06 mmol), N, N-dimethylbarbituric acid (0.36 g，2.33 mmol) and tetra(triphenylphosphine) palladium (0.12 g，0.11 mmol) were dissolved in 10.0 mL of THF and stirred at room temperature for 1 h. Upon completion of the reaction, the reaction mixture was concentrated and purified on a silica gel column eluted with methanol in methylene chloride (1.0 - 6.0%) to provide 0.85 g of compound **17**. Yield: 69%.

^1^H NMR (400 MHz, CDCl_3_) δ 8.1 (d, *J* = 7.6 Hz, 2H), 7.61 (t, *J* = 7.2 Hz, 1H), 7.50 (t, *J* = 7.6 Hz, 2H), 7.41 - 7.30 (m, 5H), 6.21 (t, *J* = 8.8 Hz, 1H),5.73 - 5.60 (m, 1H), 5.42 (m, 1H), 5.32 - 5.18 (m, 2H), 4.95 (m, 1H), 4.62 (s, 1H), 4.55 (s, 1H), 4.31 (m, 2H), 4.28 - 4.07 (m, 5H), 4.01 (s, 1H), 3.91 (m, 1H), 3.27 (s, 1H), 2.58 (m, 2H), 2.45 (s, 1H), 2.38 (m, 3H), 2.27 (d, *J* = 8.0 Hz, 2H), 2.04 (m, 2H), 1.95 (d, J = 8.8 Hz 1H), 1.85 (m, 5H), 1.75 (m, 4H), 1.34 (s, 9H), 1.35 - 1.21 (m, 8H), 1.16 (s, 3H), 1.06 (m, 1H), 0.99 (m, 1H), 0.56 (m, 2H).

^13^C NMR (100 MHz, CDCl_3_) δ 210.30, 170.23, 167.72, 166.69, 166.04, 135.02, 132.63, 129.17, 128.28, 128.03, 127.68, 127.44, 125.27, 83.32, 80.22, 77.86, 74.05, 73.47, 71.43, 70.67, 70.50, 68.61, 67.48, 60.61, 56.75, 51.25, 45.50, 42.18, 37.49, 34.16, 30.84, 28.02, 27.15, 25.36, 21.70, 21.28, 19.81, 13.84, 13.15, 8.98.

ESI-MS (*m/z*): calcd for C_60_H_77_KN_3_O_20_ [M+K]^+^: 1198.4732; found: 1198.4741.

**2.3. Preparation of final conjugate 18**

The synthetic route of compound **18** was described in Scheme 3.

The functionalized dextran **8** (329 mg) and compound **17** (182 mg, 0.16 mmol) were combined in 5.0 mL of DMSO, followed by addition of the mixture of CuSO_4_ (160 μL, 0.16 mmol) and sodium ascorbate (320 μL, 0.32 mmol) under nitrogen protection and stirred at room temperature overnight. Upon completion of the reaction, the reaction mixture was diluted with distilled water (50 mL) and washed with methylene chloride (50 mL). The aqueous phase was dialyzed against distilled water three times, concentrated, lyophilized to provide dual conjugate **17** (204mg). Yield: 40%.

^1^H NMR (selected characteristic signals, 400 MHz, DMSO-*d*_6_+D_2_O, ppm): major signals: 4.50 - 5.00 (m, CHOH), 3.30 - 4.20 (m, CHOH, CH_2_OH); minior signals: 7.10 – 8.50 (m, CONH, ArH), 5.30 (m, =CH), 1.23 (m, CH_3_), 0.95 - 1.10 (m, CH_3_).

**2.4. Preparation of compound Dextran-DTX 19**

The synthetic route of Dextran-DTX was described as scheme S1.

**Scheme S1.** The synthetic route of Dextran-DTX.

The di-functionalized dextran and compound **19** were prepared using the methods similar to those described for functionalized compound **8** and compound **18**, respectively.

^1^H NMR (selected characteristic signals, 400 MHz, DMSO-*d*_6_+D_2_O, ppm): major signals: 4.50 - 5.00 (m, CHOH), 3.30 - 4.20 (m, CHOH, CH_2_OH); minior signals: 7.20 – 8.00 (m, CONH, ArH), 0.80 - 1.20 (m, CH_3_).

**2.5. Preparation of compound Dextran-DHA 20**

The synthetic route of Dextran-DHA was described as scheme S2.

^^

**Scheme S2.** The synthetic route of Dextran-DHA.

2.0 g of dextran with average molecular weight of 100k daltons was totally dried in oil bath at 60^o^C under high vacuum for 10 h, and then dissolved in 10 mL of anhydrous DMSO at 60^o^C. After cooled down to room temperature, the oil bath was removed. To the above solution, dimethyl (S)-2-isocyanatopentanedioate (3.98 g, 19.8 mmol) , compound **4** (0.55 g, 1.07 mmol) and DMAP (7.65 g, 62.6 mmol) were slowly added and stirred at room temperature overnight. After the completion of the reaction, the reaction mixture was directly dialyzed against distilled water for 24 h, then concentrated and hydrolyzed with NaOH (3.34 g, 83.5 mmol) for 5 h. The resulting solution was adjusted to pH 3.0 - 5.0 with 1 N HCl solution, and continued to dialyzed against distilled water three times, concentrated, and lyophilized to provide compound Dextran-DHA 19 (1.24 g). Yield: 29%.

^1^H NMR (selected characteristic signals, 400 MHz, DMSO-d_6_, ppm): .major signals: 4.50 - 5.00 (m, CHOH), 3.30 - 4.20 (m, CHOH, CH_2_OH); minior signals: 5.25 - 5.45 (m, =CH), 2.85 - 2.95 (m, CH_2_), 1.10 - 0.95 (m, CH_3_), 0.87 (t, *J* = 7.6 Hz, 3H)

**2.6. Preparation of compound Dextran-DHA-Cy7.5.**

The synthetic route of Dextran-DHA-Cy7.5 was described as scheme S3.

^^

**Scheme S3.** The synthetic route of Dextran-DHA-Cy7.5.

The functionalized dextran **8** (90 mg) and Cy7.5 (10 mg, 0.014 mmol) were combined in 5.0 mL of DMSO, followed by addition of the mixture of CuSO_4_ (14 μL, 0.014 mmol) and sodium ascorbate (28 μL, 0.028 mmol) under nitrogen protection and stirred at room temperature overnight. Upon completion of the reaction, the reaction mixture was diluted with distilled water (50 mL) and washed with methylene chloride (50 mL). The aqueous phase was dialyzed against distilled water three times, concentrated, lyophilized to provide dual conjugate **21** (33 mg). Yield: 33%.

^1^H NMR (selected characteristic signals, 400 MHz, DMSO-d_6_+D_2_O): major signals: δ δ 4.50 - 5.00 (m, CHO), 4.00 - 3.50 (m, CHOH, CH_2_OH); minior signals: δ 8.50 - 7.25 (m, ArH), 5.30 (m, =CH), 2.00 - 1.80 (m, CH_3_).

**2.7. Preparation of compound Dextran-Cy7.5**

The synthetic route of Dextran-Cy7.5 was described as scheme S4.

^^

**Scheme S4.** The synthetic route of Dextran-Cy7.5.

The title conjugate was prepared using a method similar to that described for compound 21.

^1^H NMR (selected characteristic signals, 400 MHz, DMSO-d_6_+D_2_O): major signals: δ δ 4.80 - 5.20 (m, CHO), 4.20 - 3.70 (m, CHOH, CH_2_OH); minior signals: δ 8.50 - 7.25 (m, ArH), 2.02 - 1.80 (m, CH_3_).

**3. ^1^H NMR, ^13^C NMR and MS spectra of Compounds 1 to 18**

**3.1. ^1^H NMR,****^13^C NMR and MS spectra of compound 1**


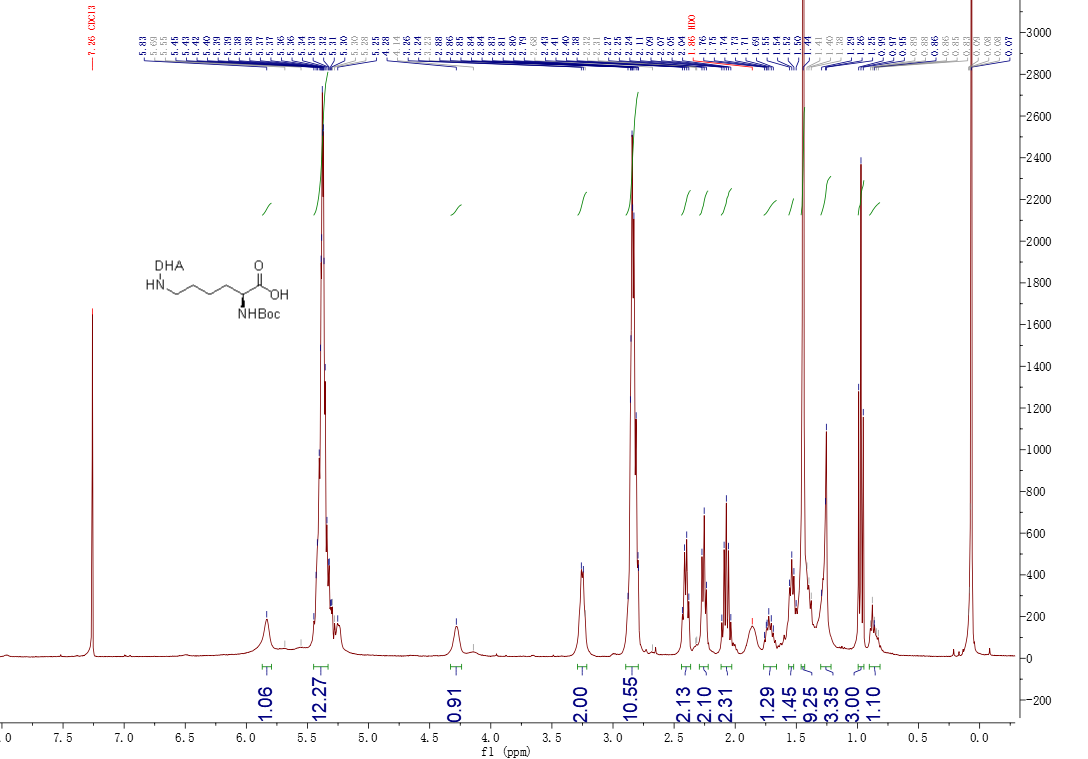


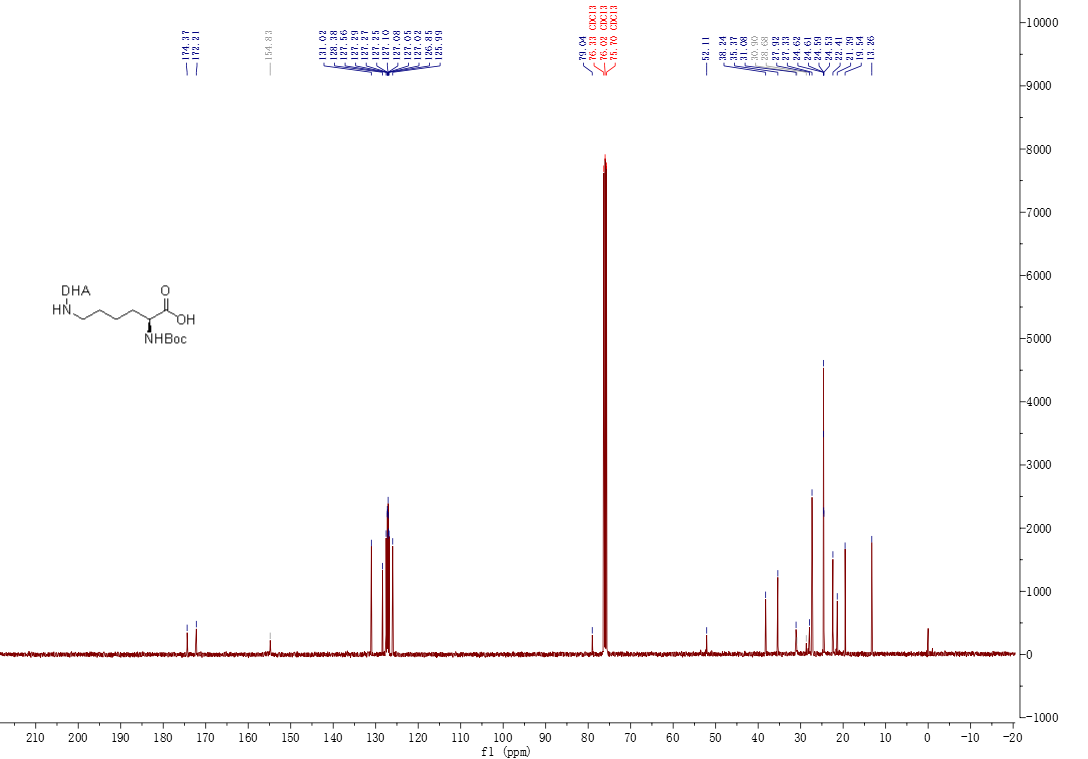


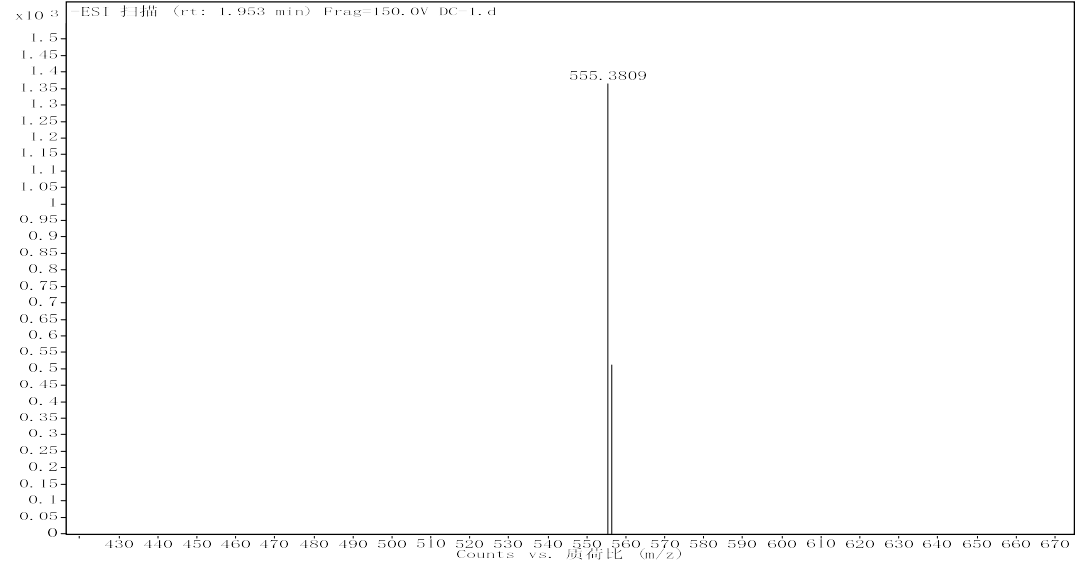


**3.2. ^1^H NMR,^13^C NMR and MS spectra of compound 2**


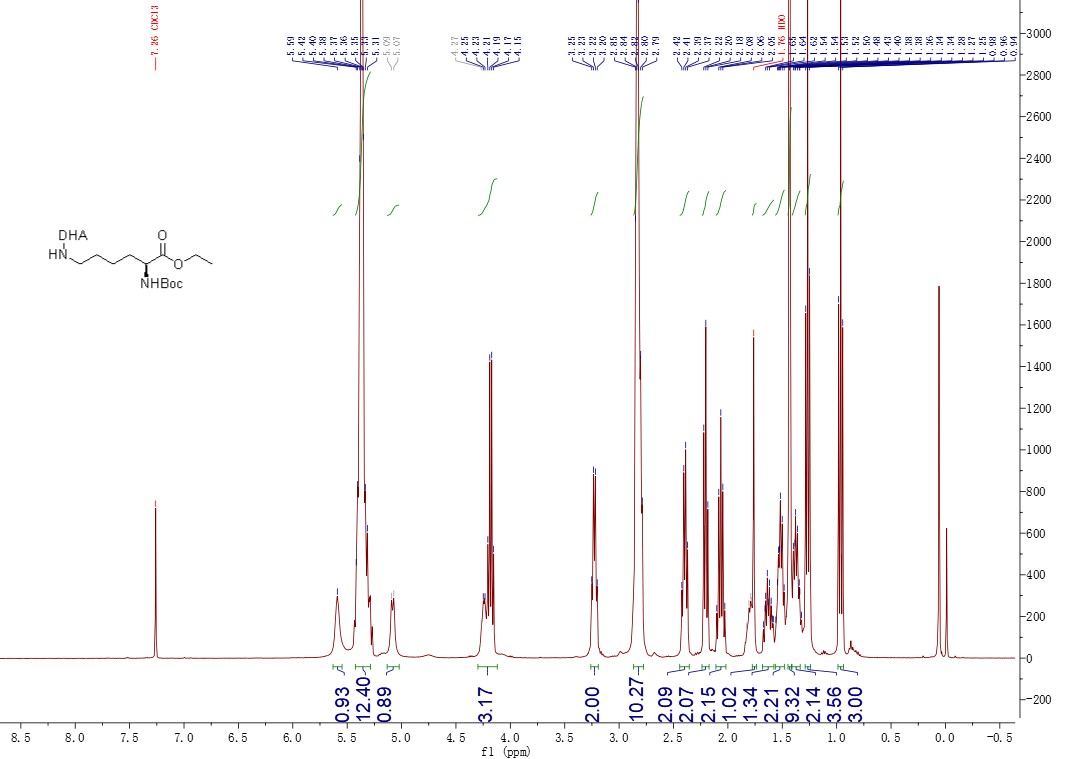


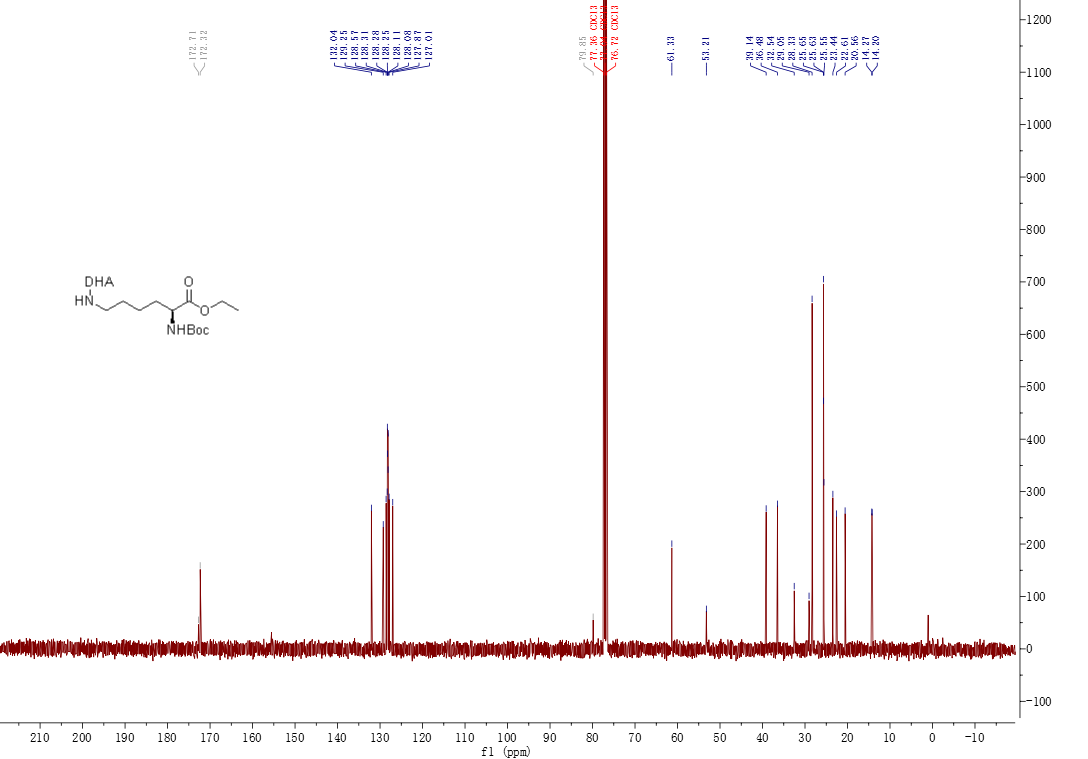


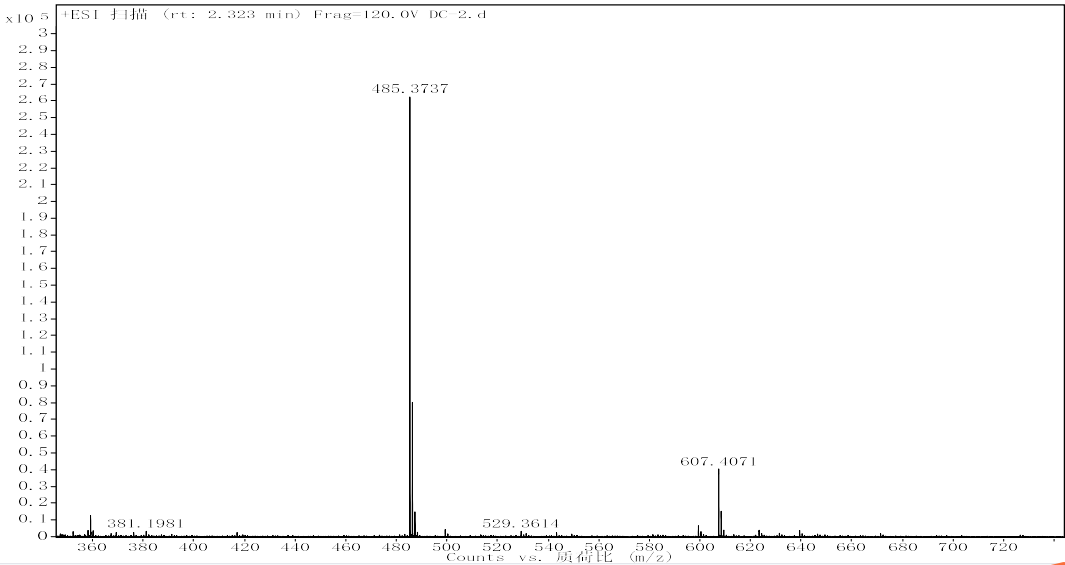


**3.3. ^1^H NMR,^13^C NMR and MS spectra of compound 3**


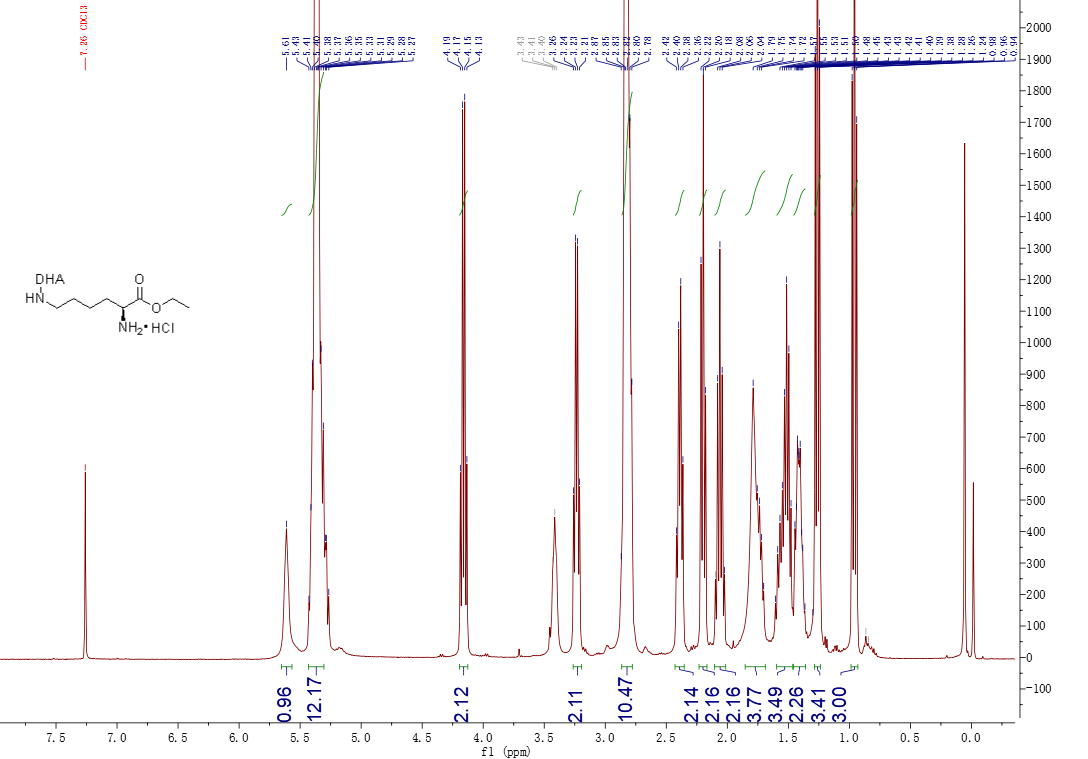


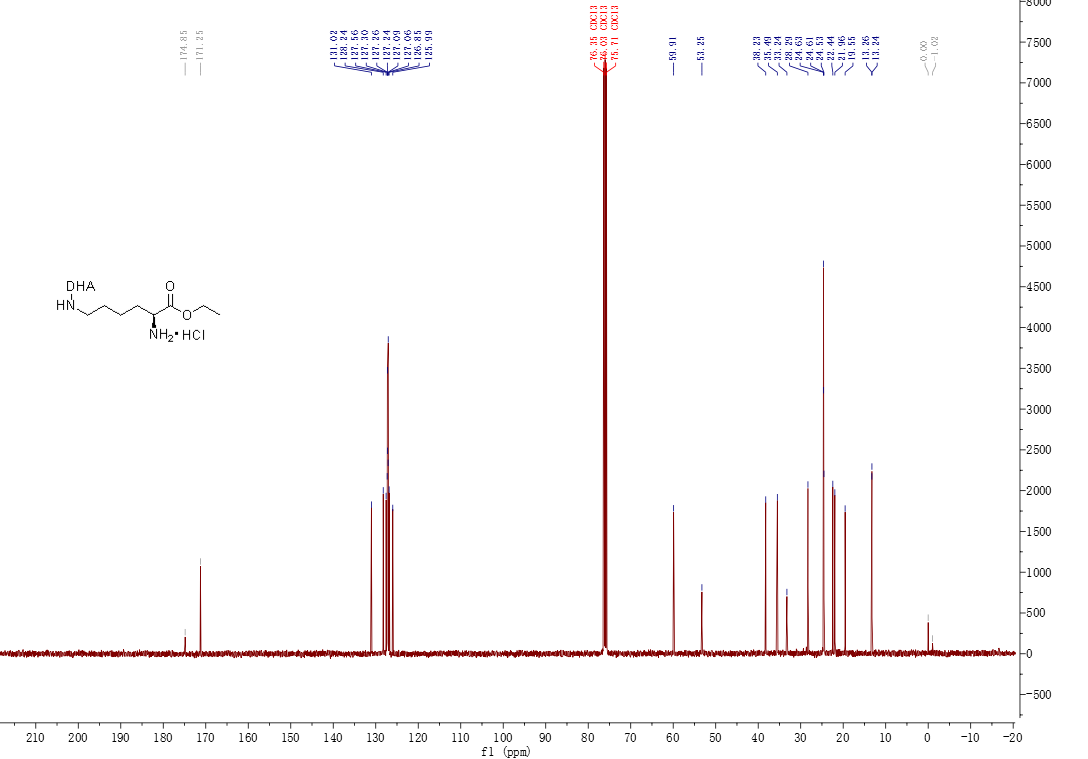


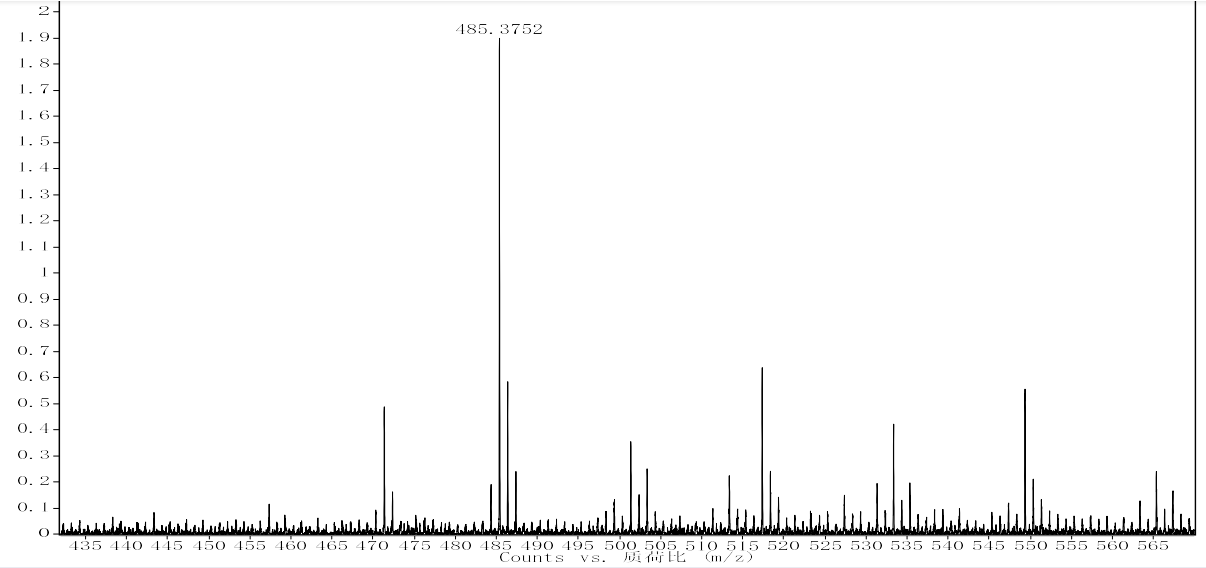


**3.4. ^1^H NMR,^13^C NMR and MS spectra of compound 4**


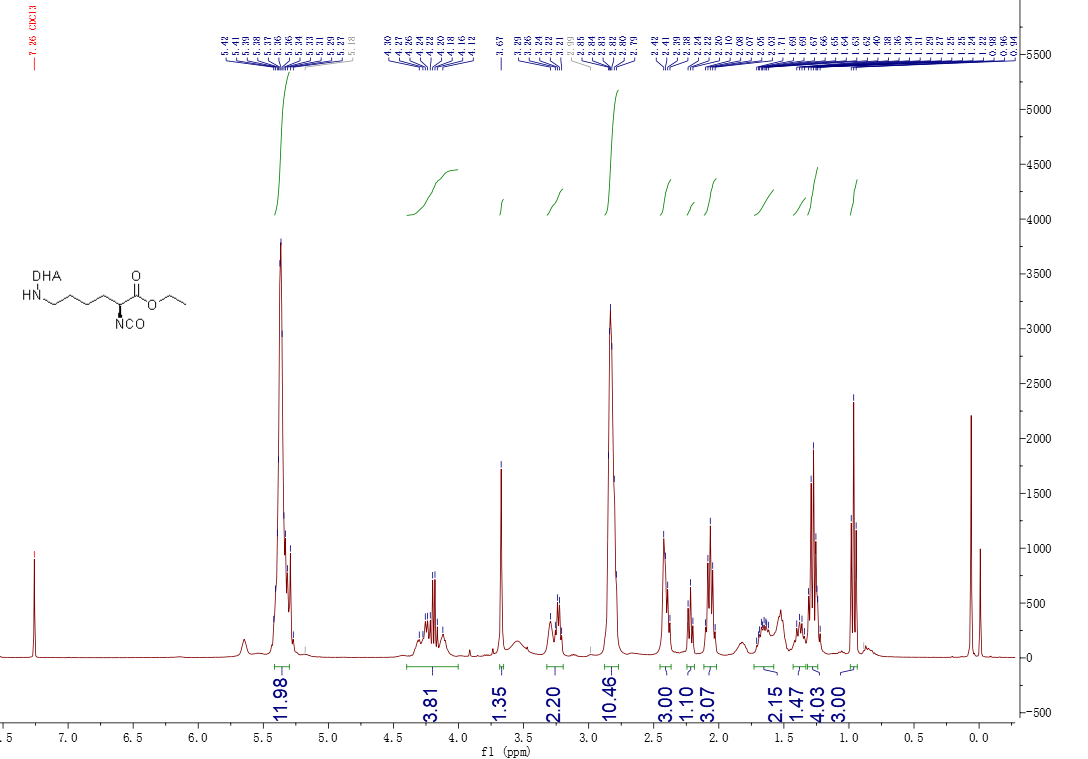


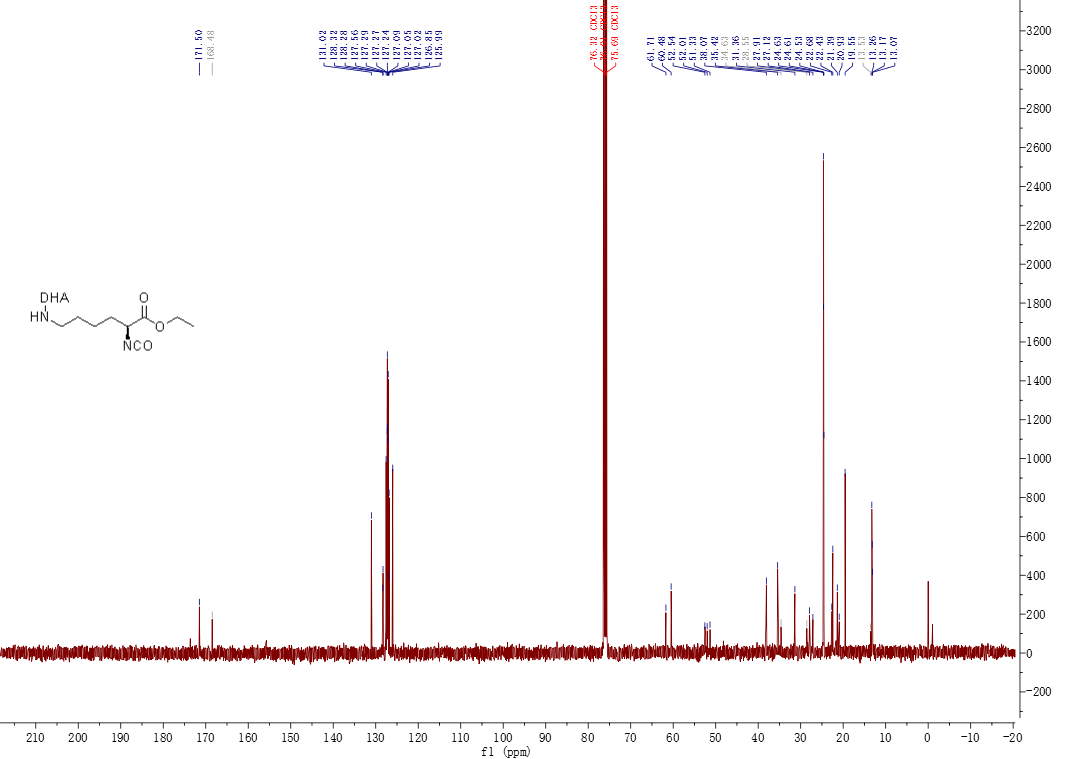

**3.5. ^1^H NMR,^13^C NMR and MS spectra of compound 5**


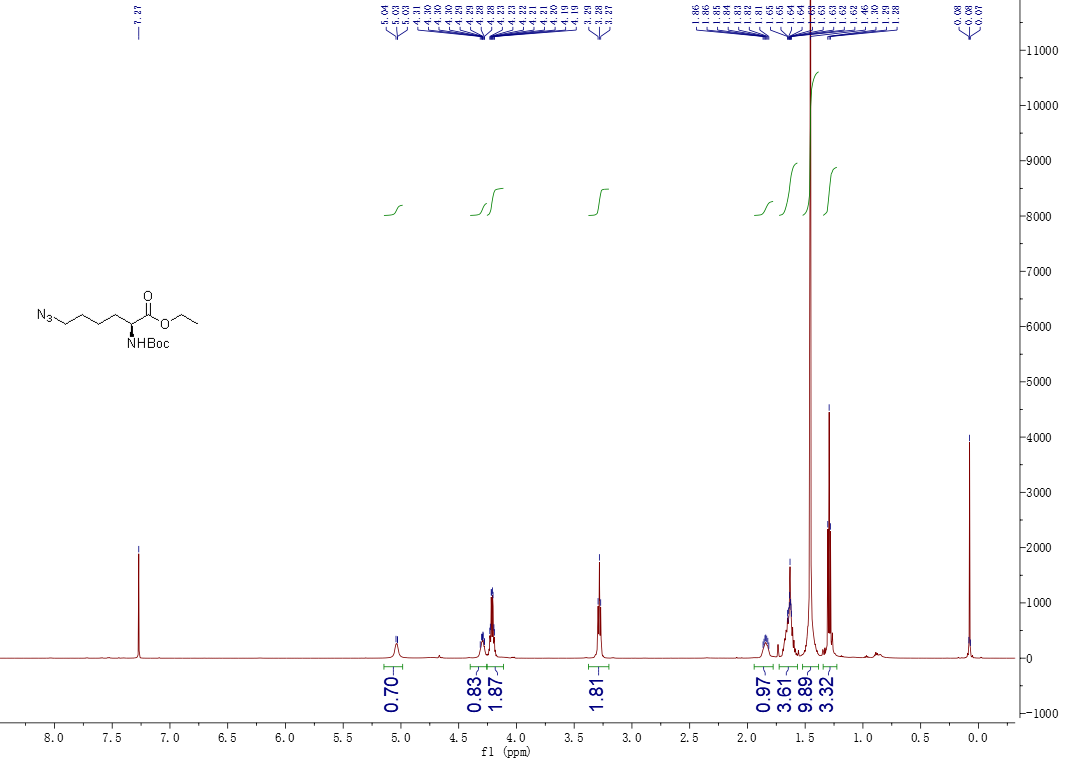


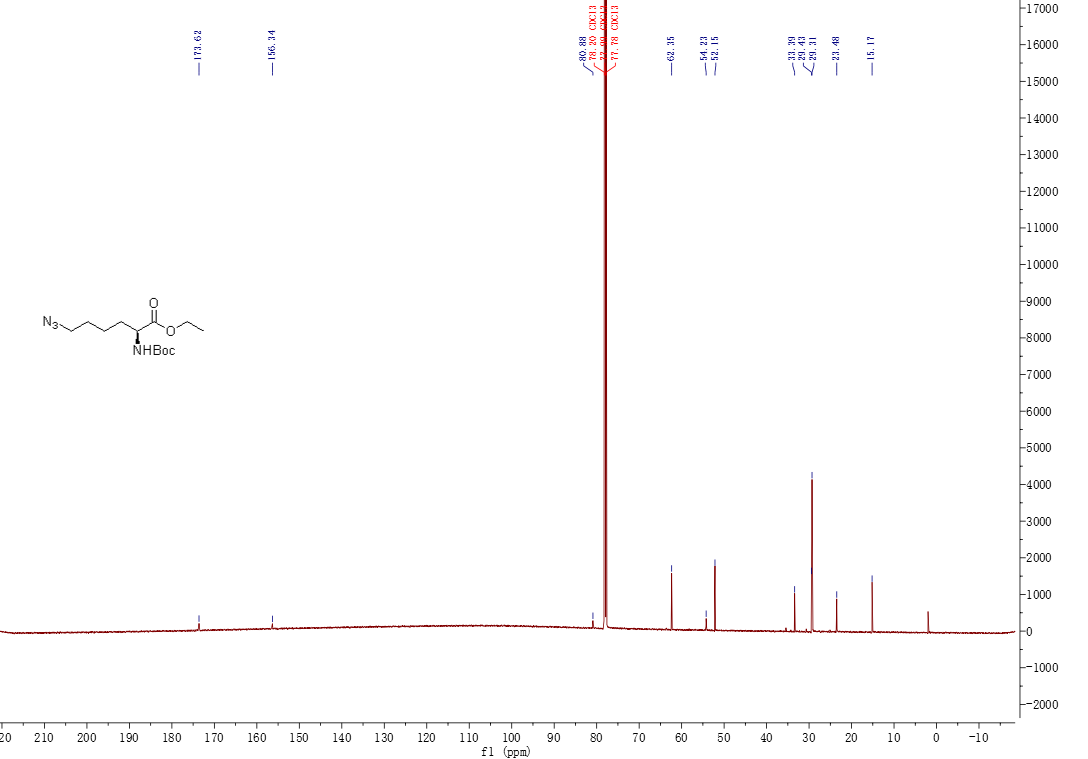


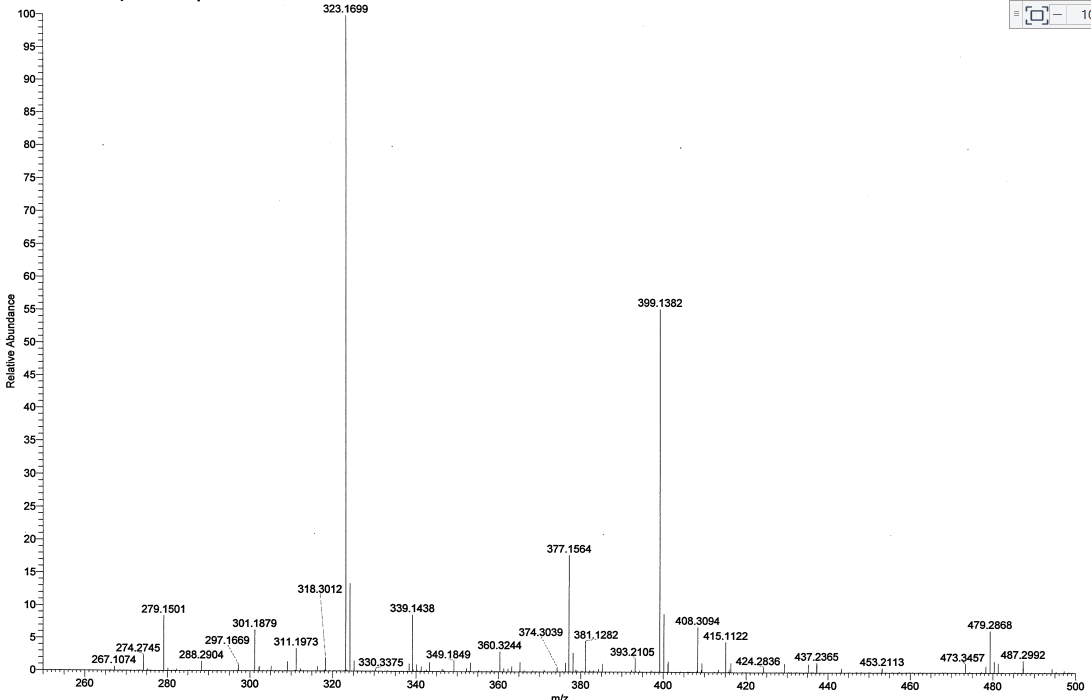


**3.6. ^1^H NMR,^13^C NMR and MS spectra of compound 6**


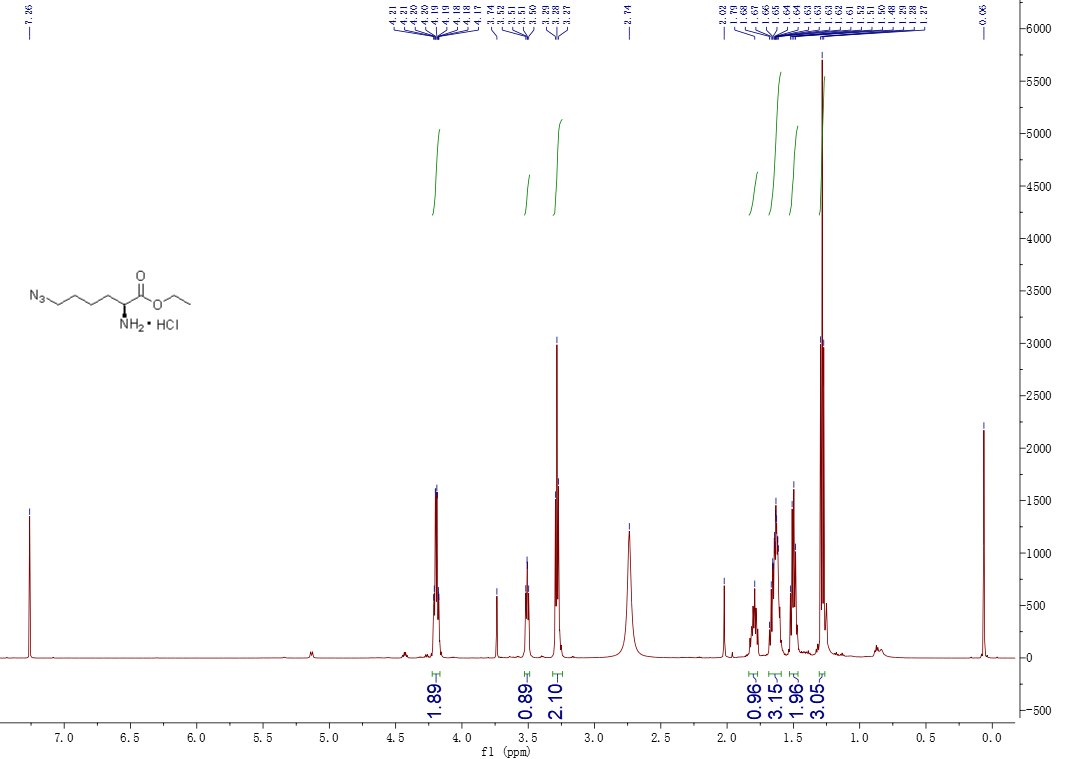


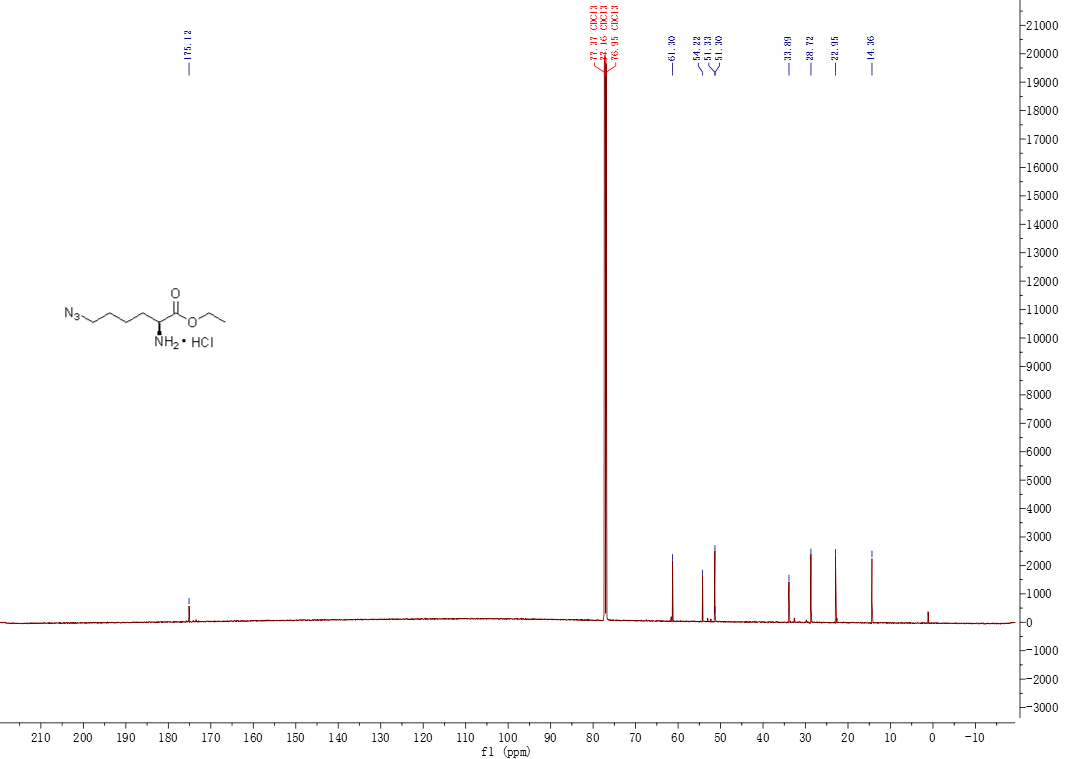


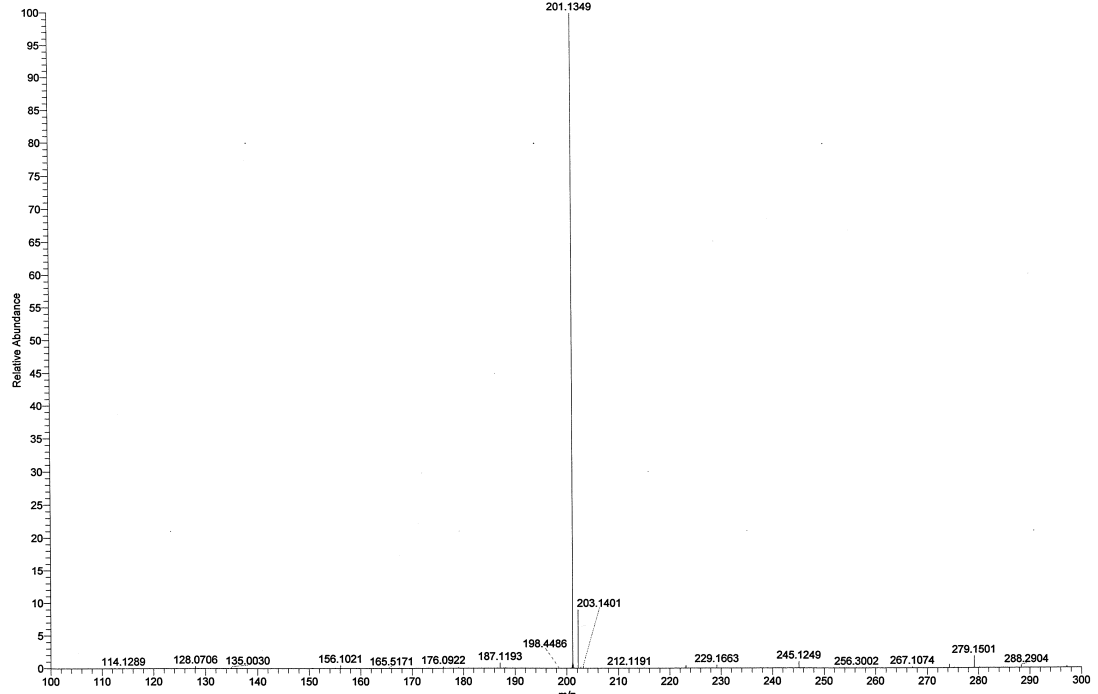


**3.7. ^1^H NMR,^13^C NMR and MS spectra of compound 7**


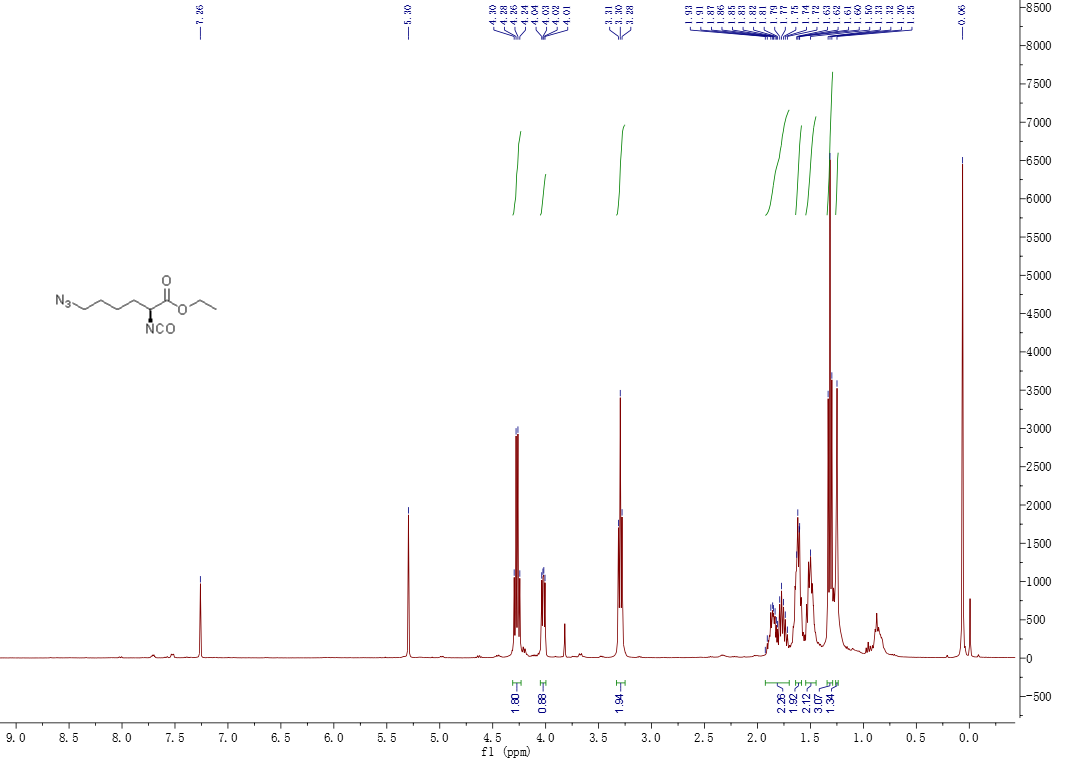


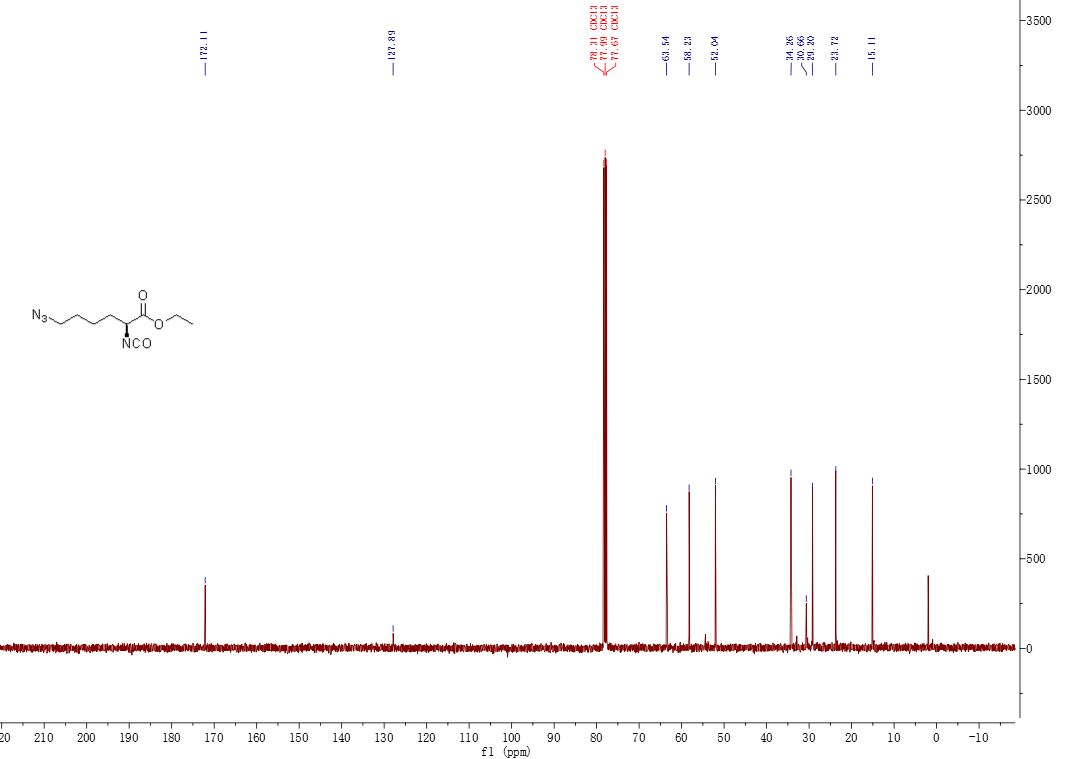

**3.8. ^1^H NMR spectrum of compound 8**


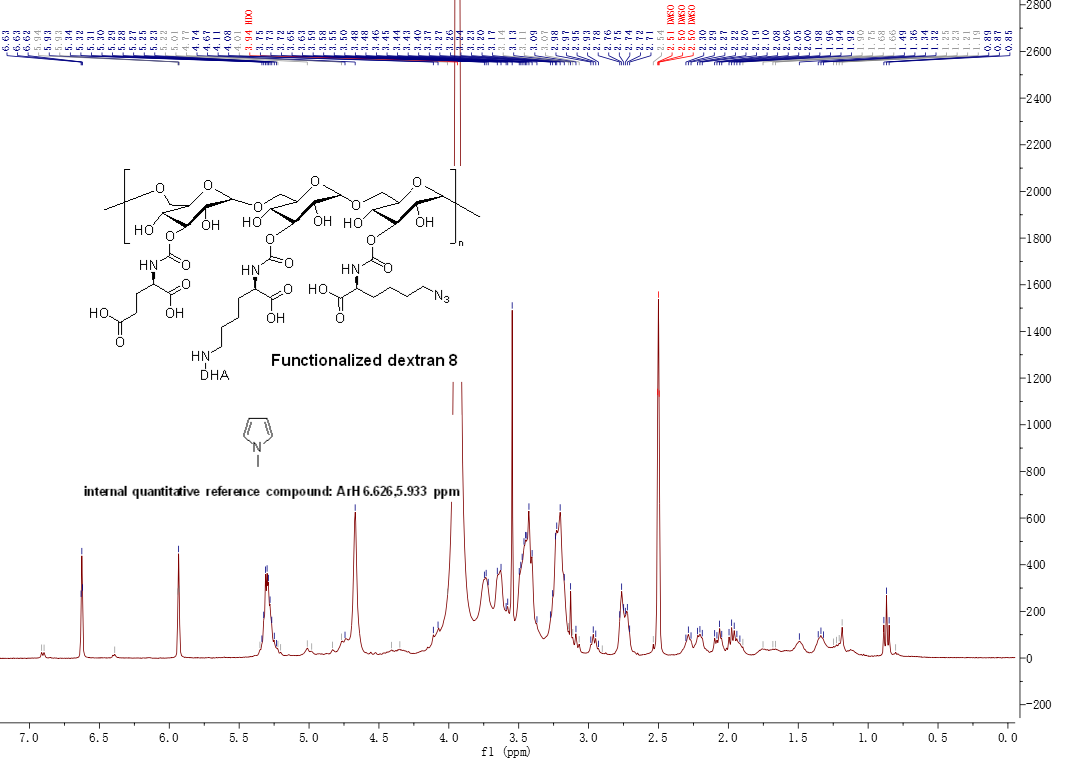


**3.9. ^1^H NMR,^13^C NMR and MS spectra of compound 9**


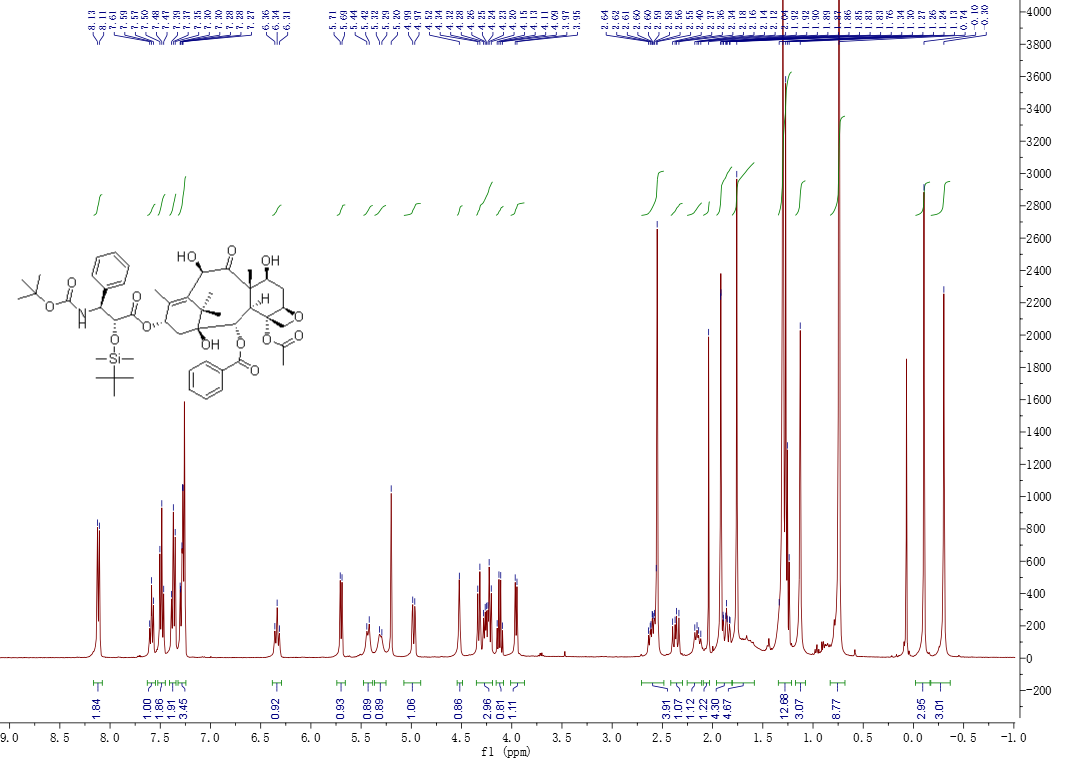


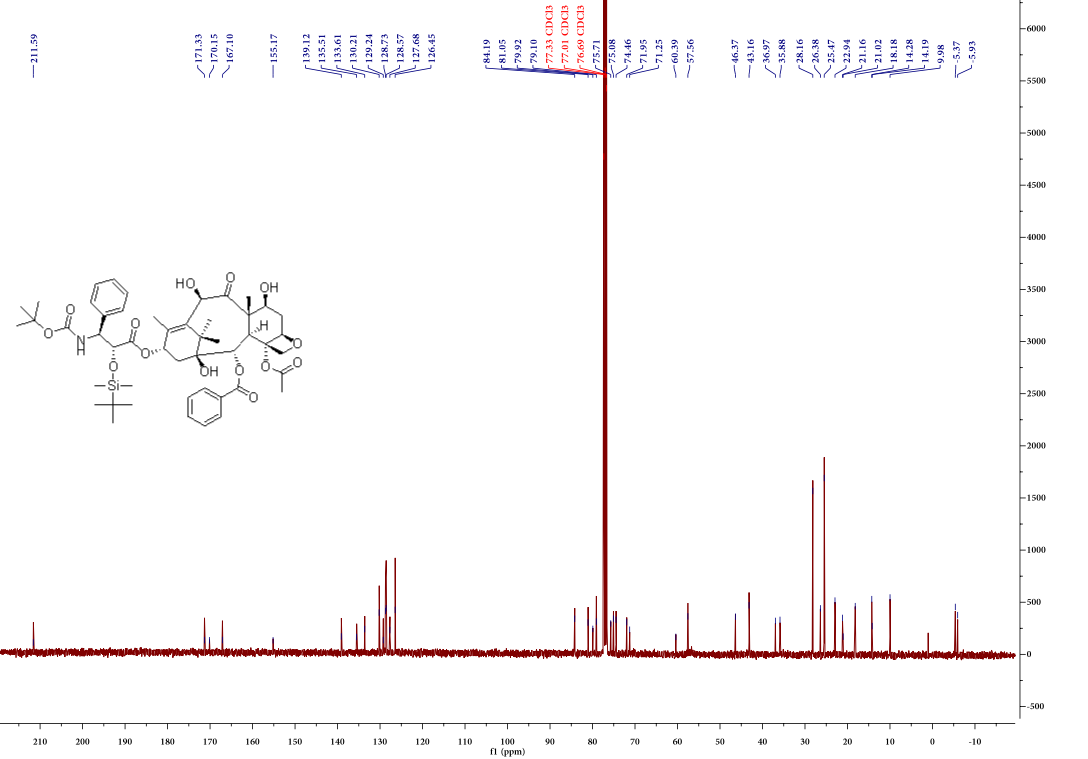

**3.10. ^1^H NMR,^13^C NMR and MS spectra of compound 10**


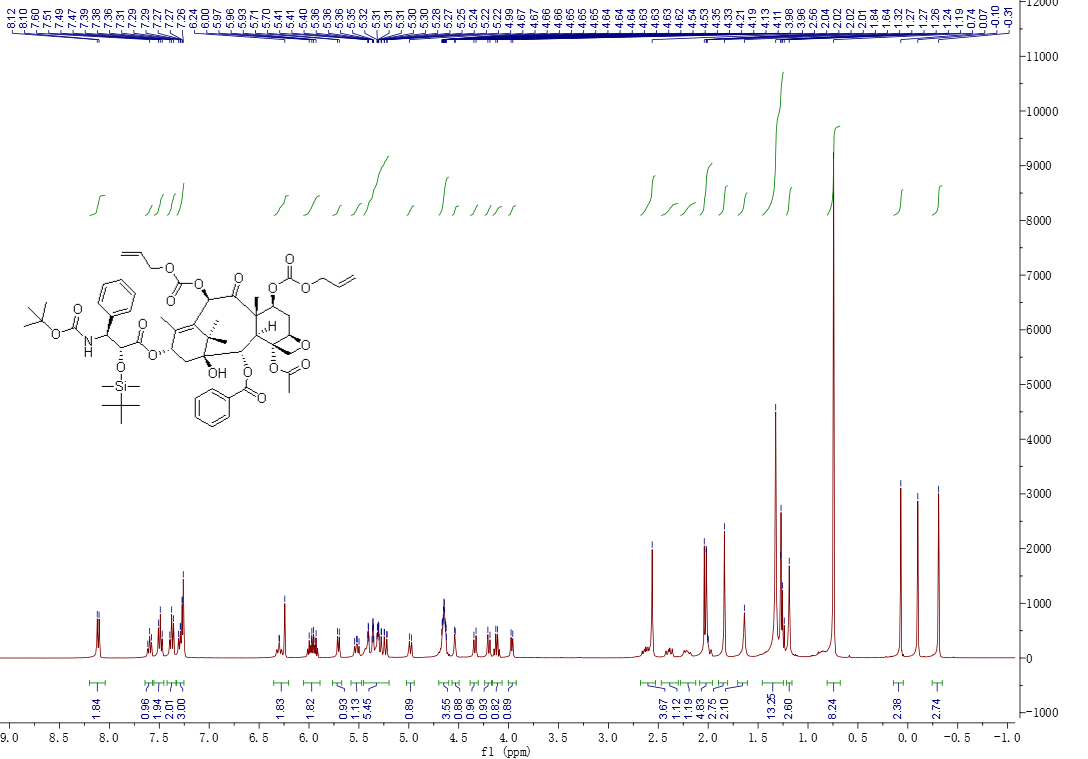


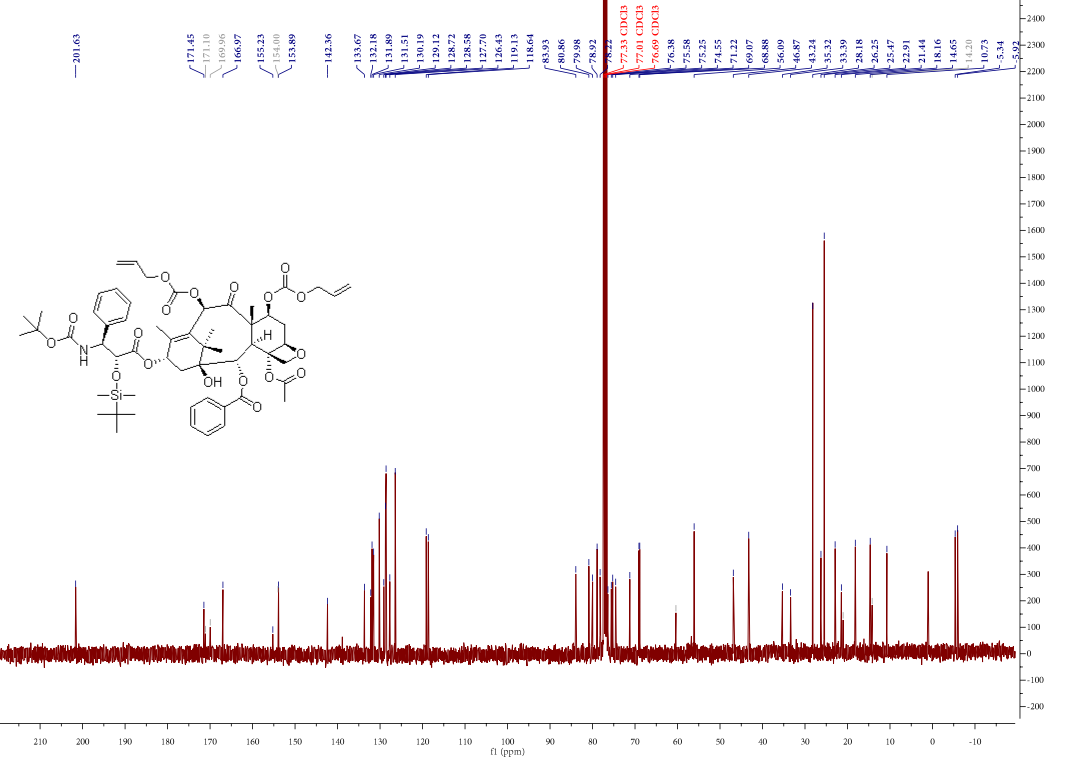

**3.11. ^1^H NMR,^13^C NMR and MS spectra of compound 11**


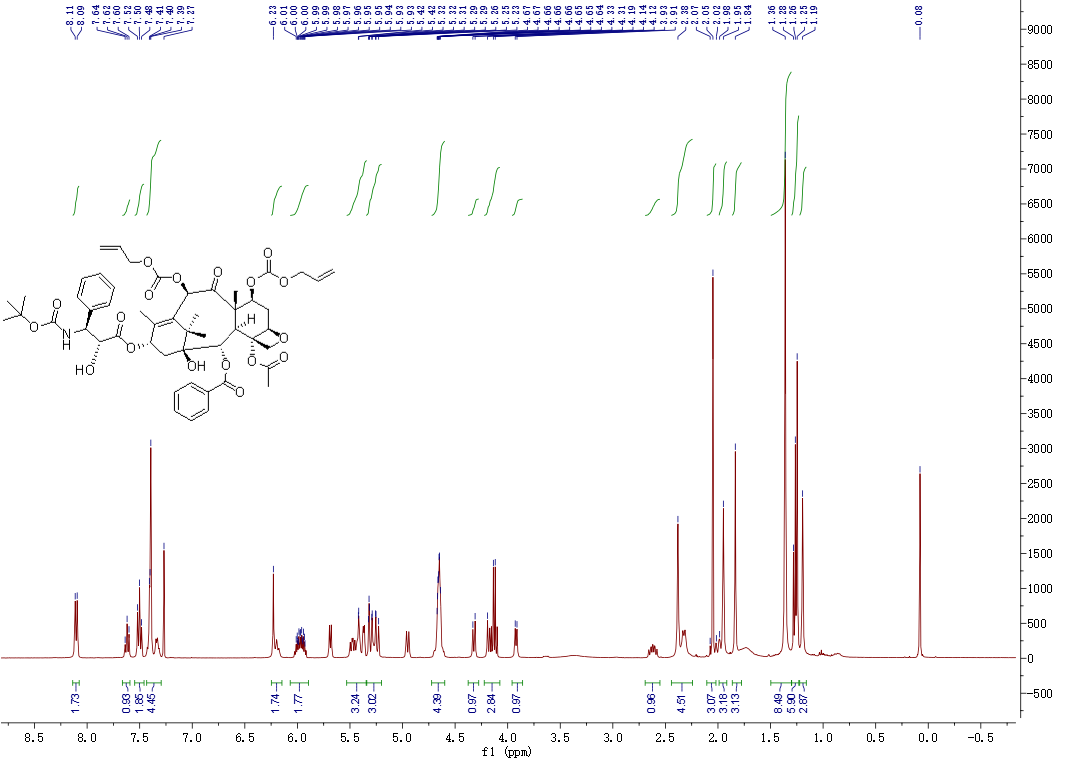


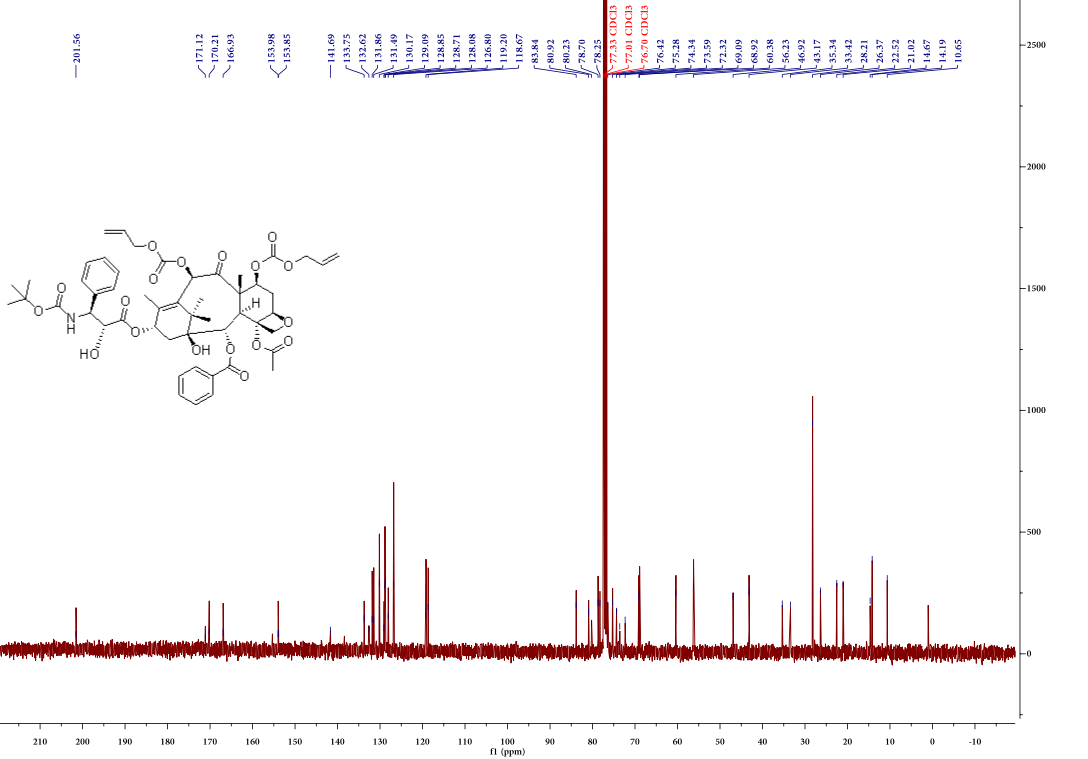

**3.12. ^1^H NMR,^13^C NMR and MS spectra of compound 12**


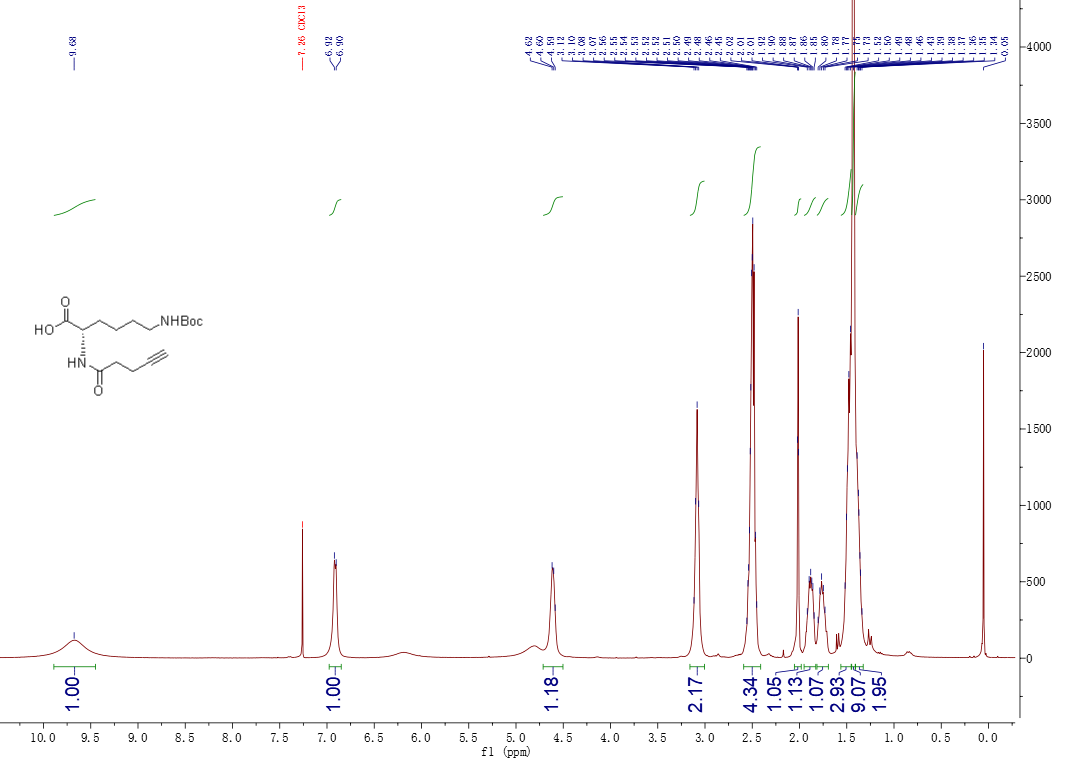

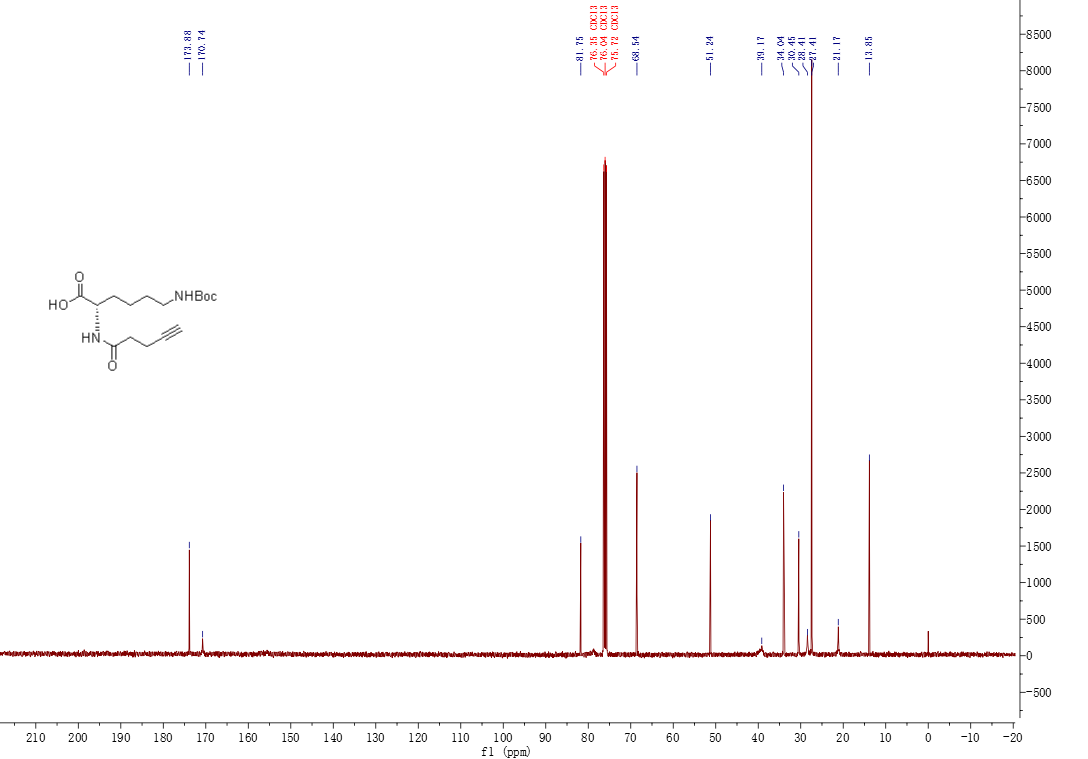


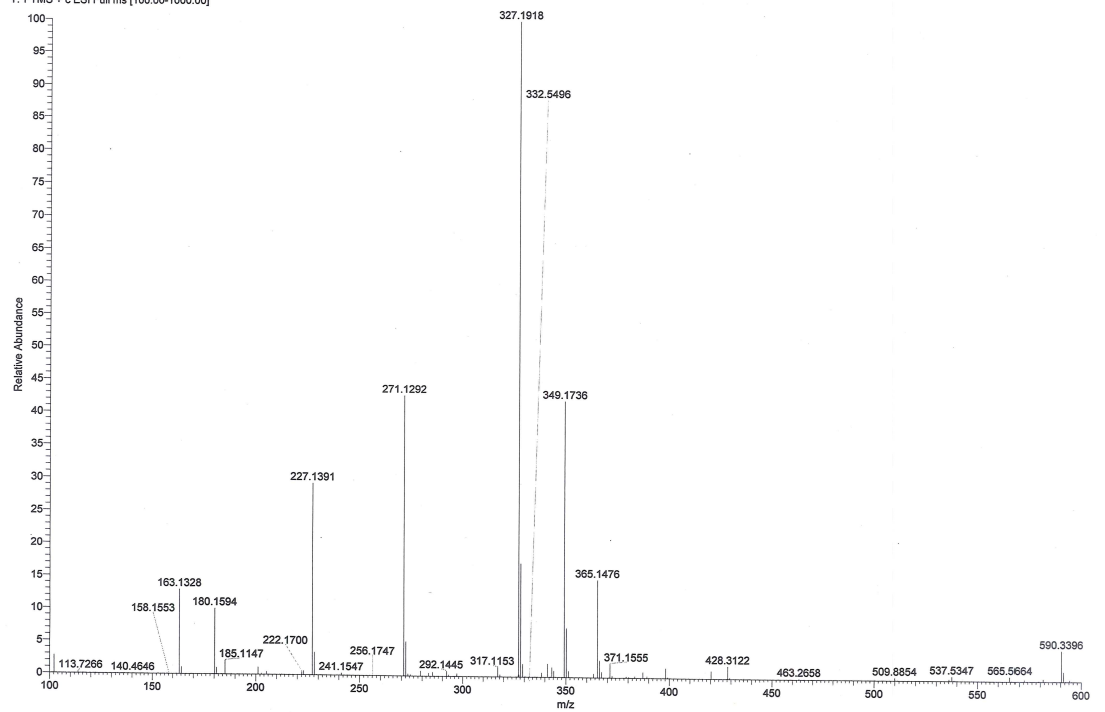


**3.13. ^1^H NMR,^13^C NMR and MS spectra of compound 13**


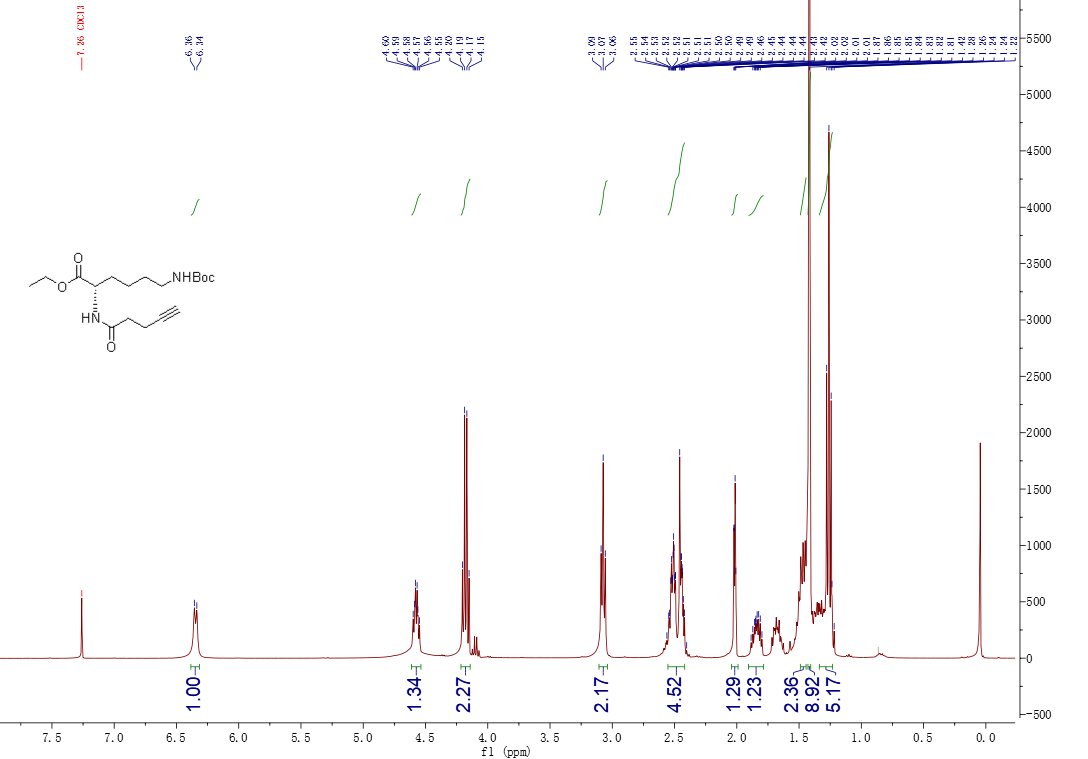


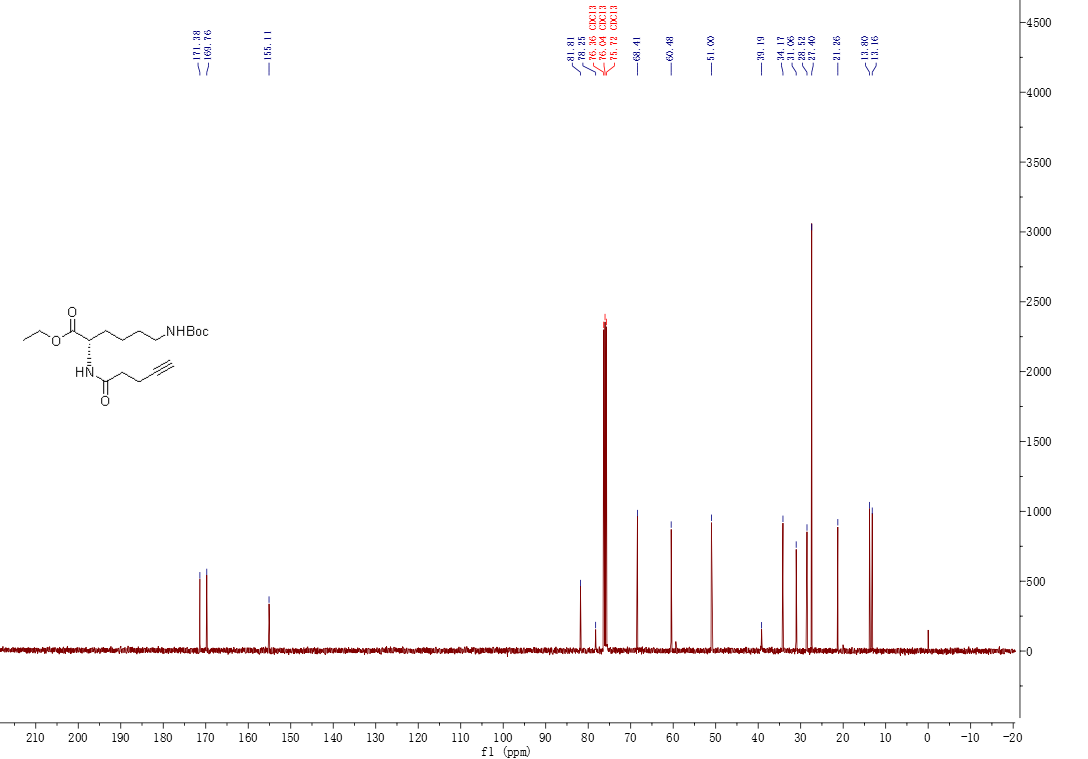

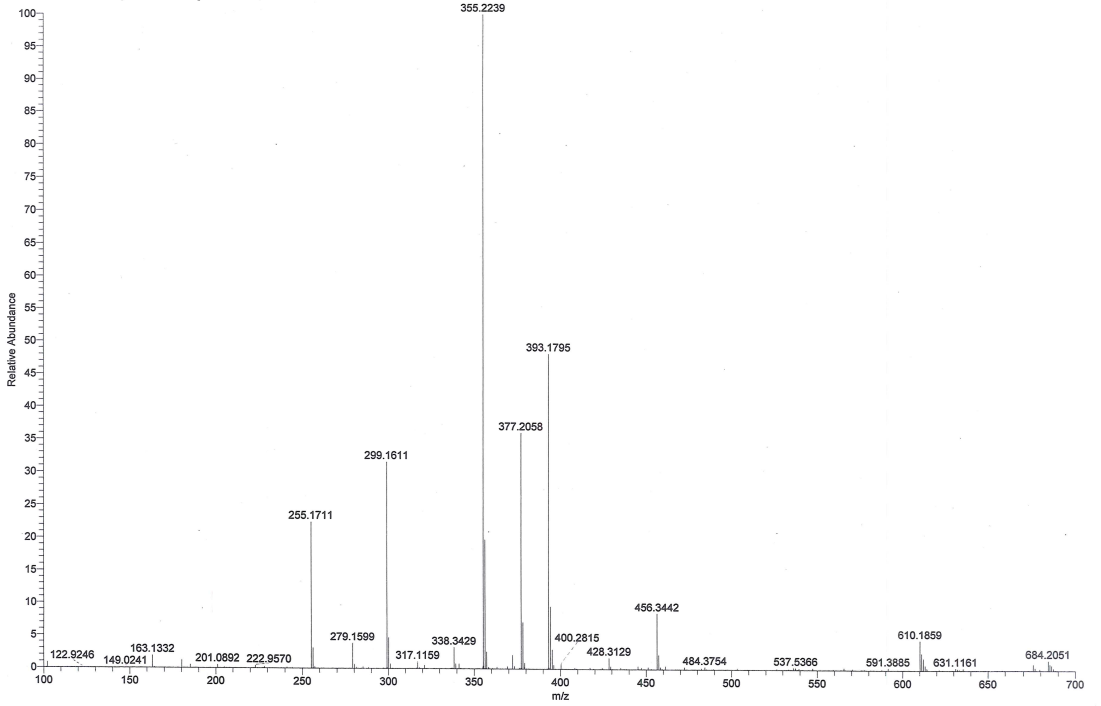
**3.14. ^1^H NMR,^13^C NMR and MS spectra of compound 14**


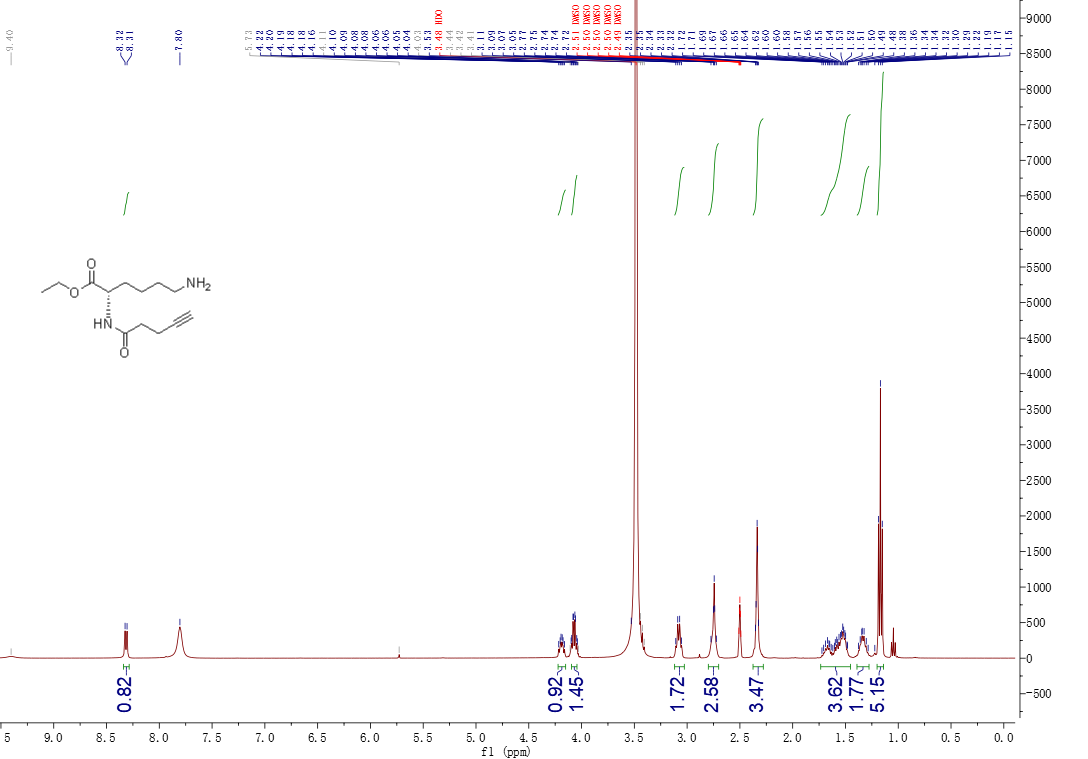

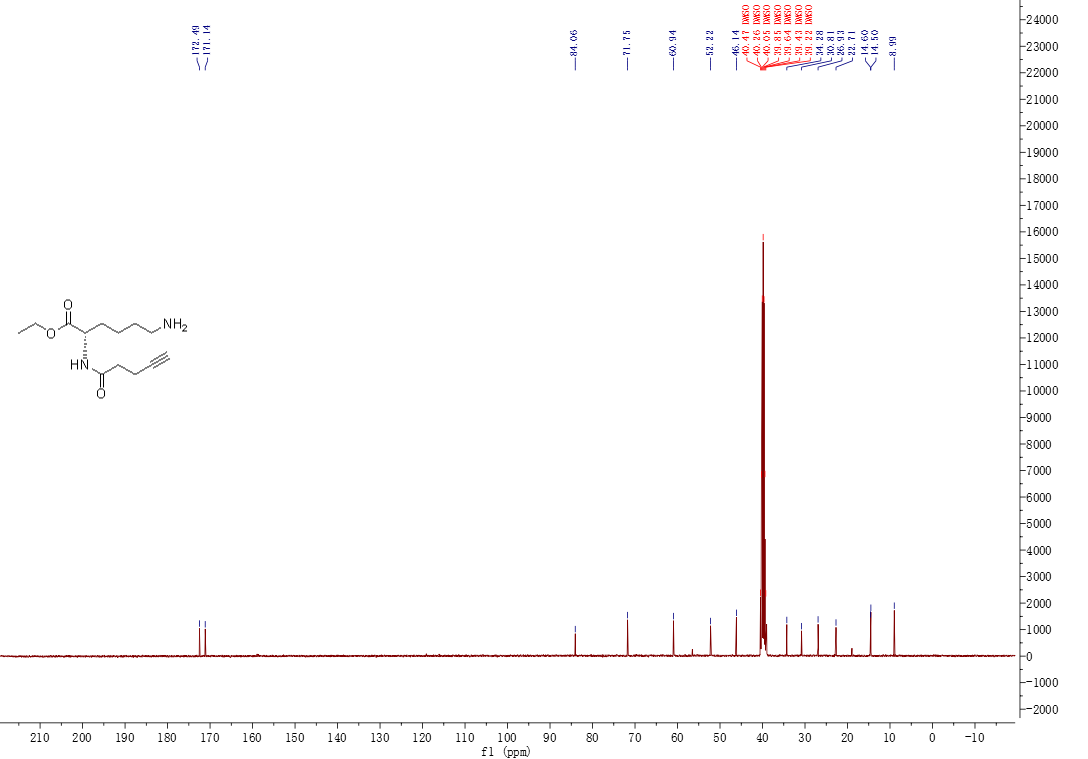


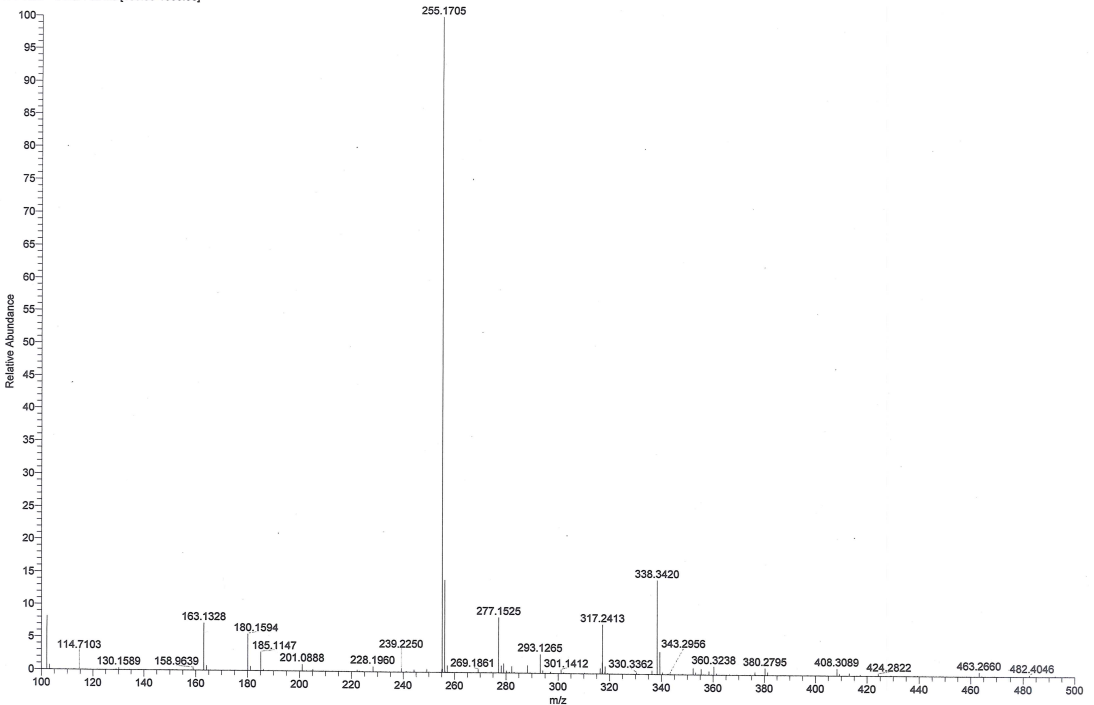


**3.15. ^1^H NMR,^13^C NMR and MS spectra of compound 15**


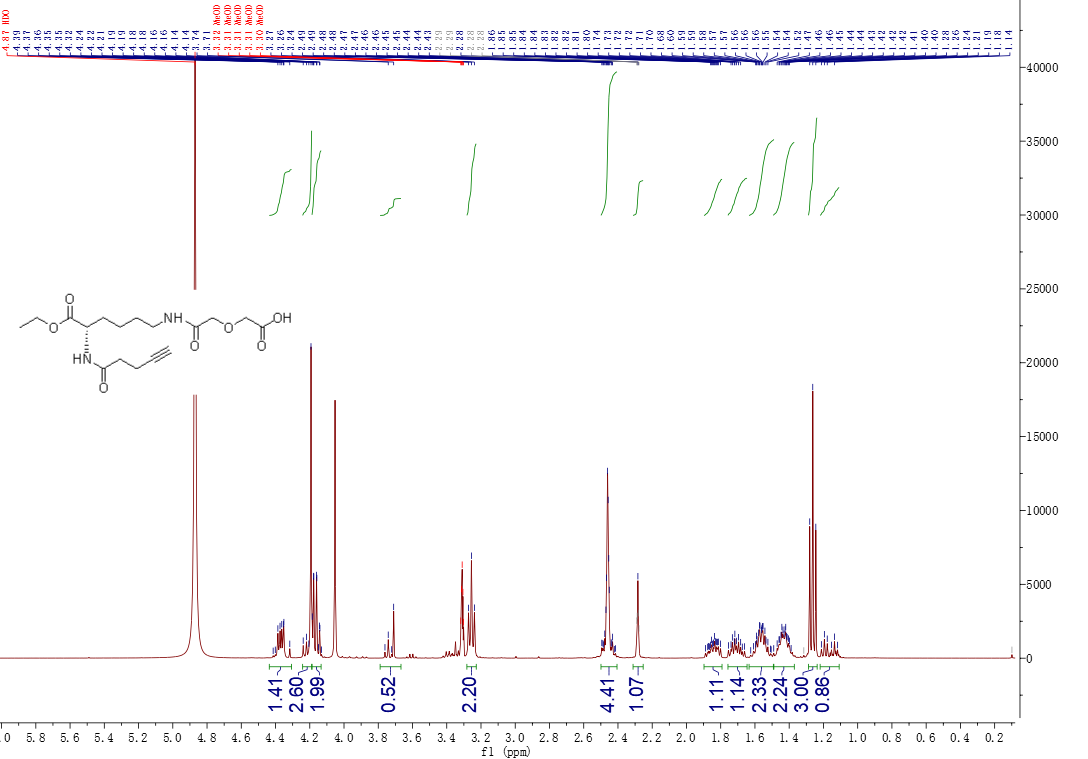

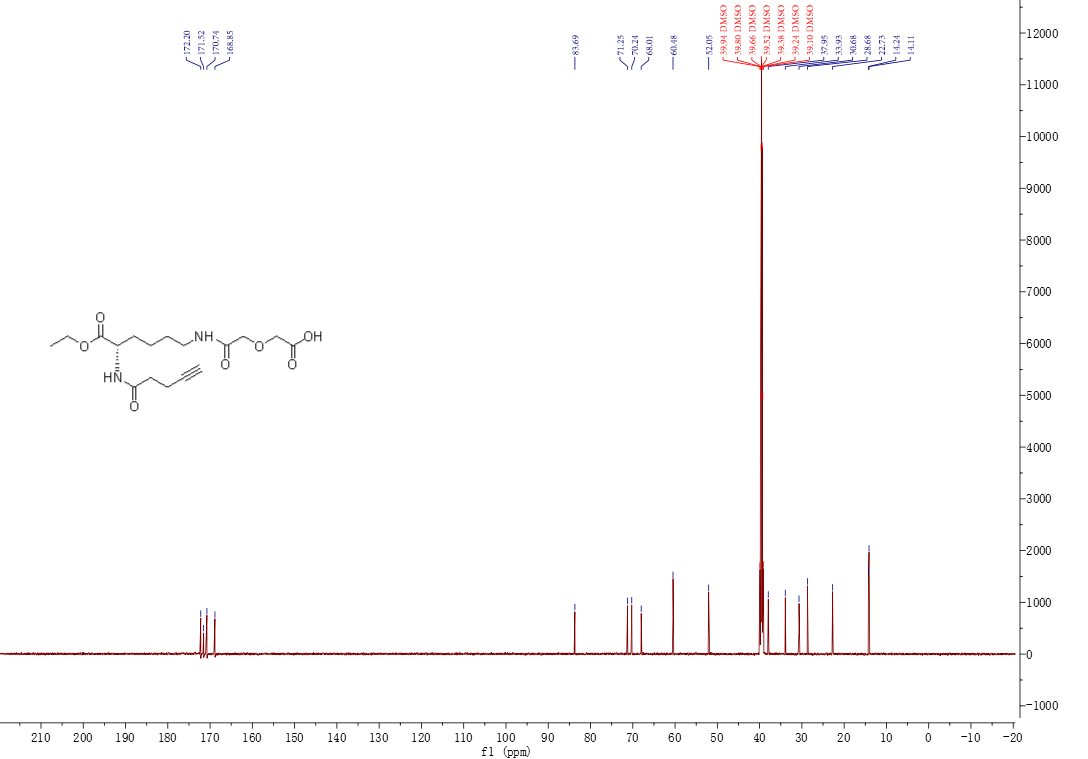


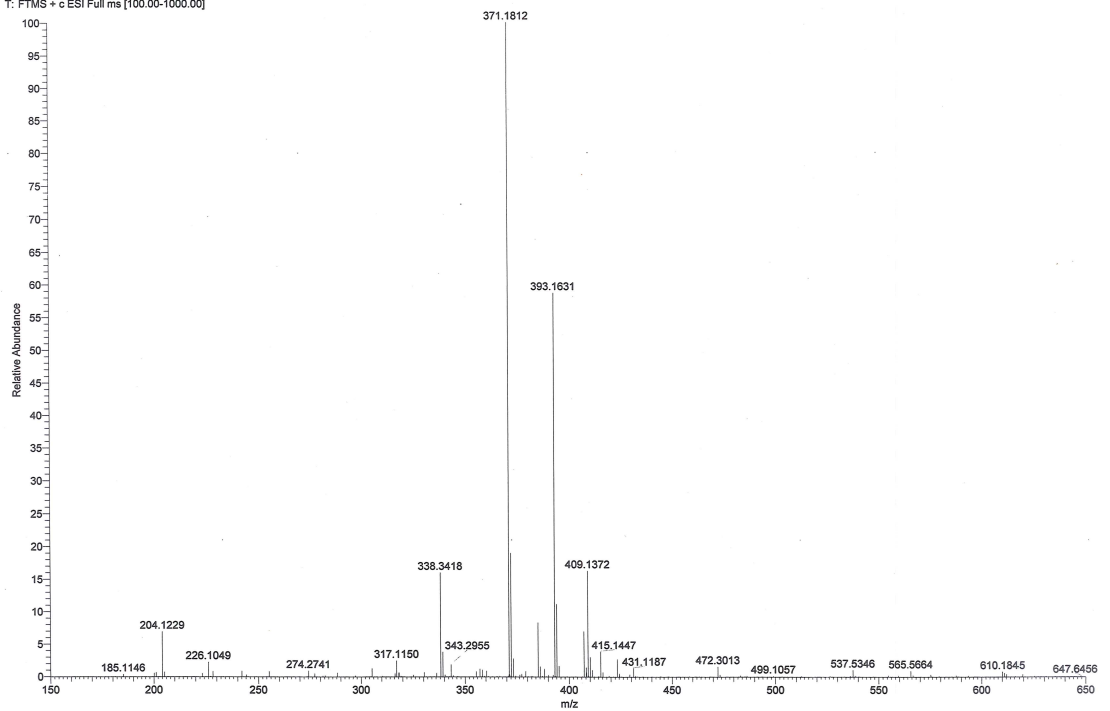


**3.16. ^1^H NMR,^13^C NMR and MS spectra of compound 16**


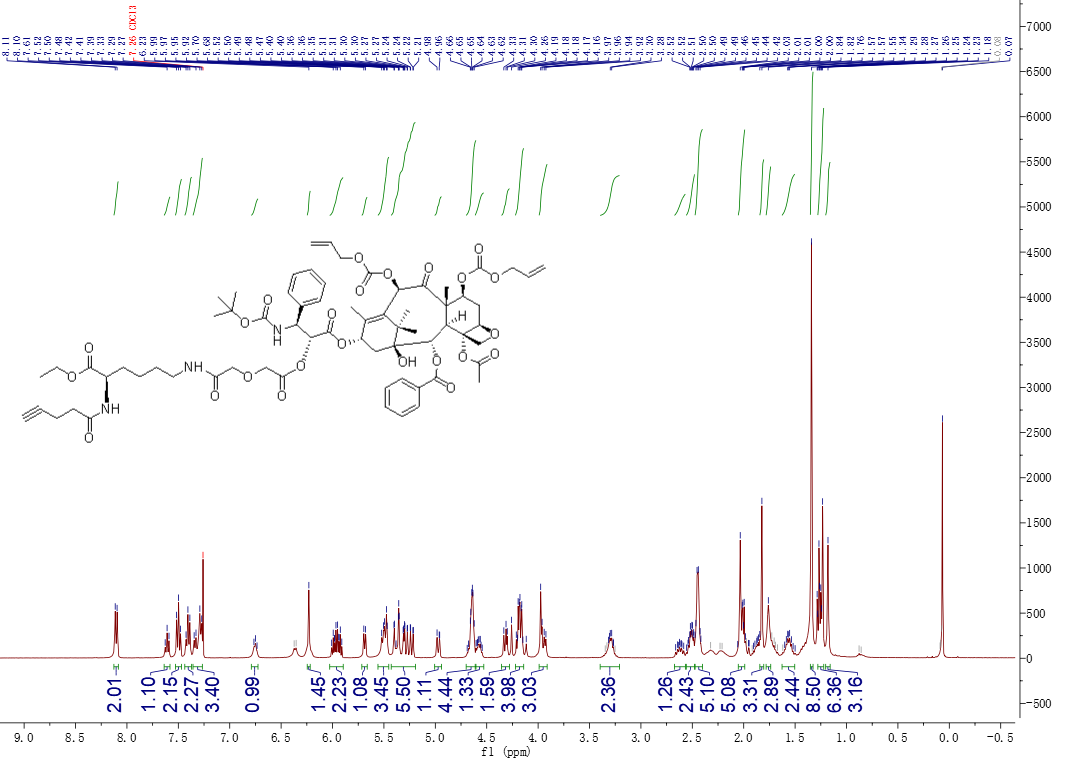


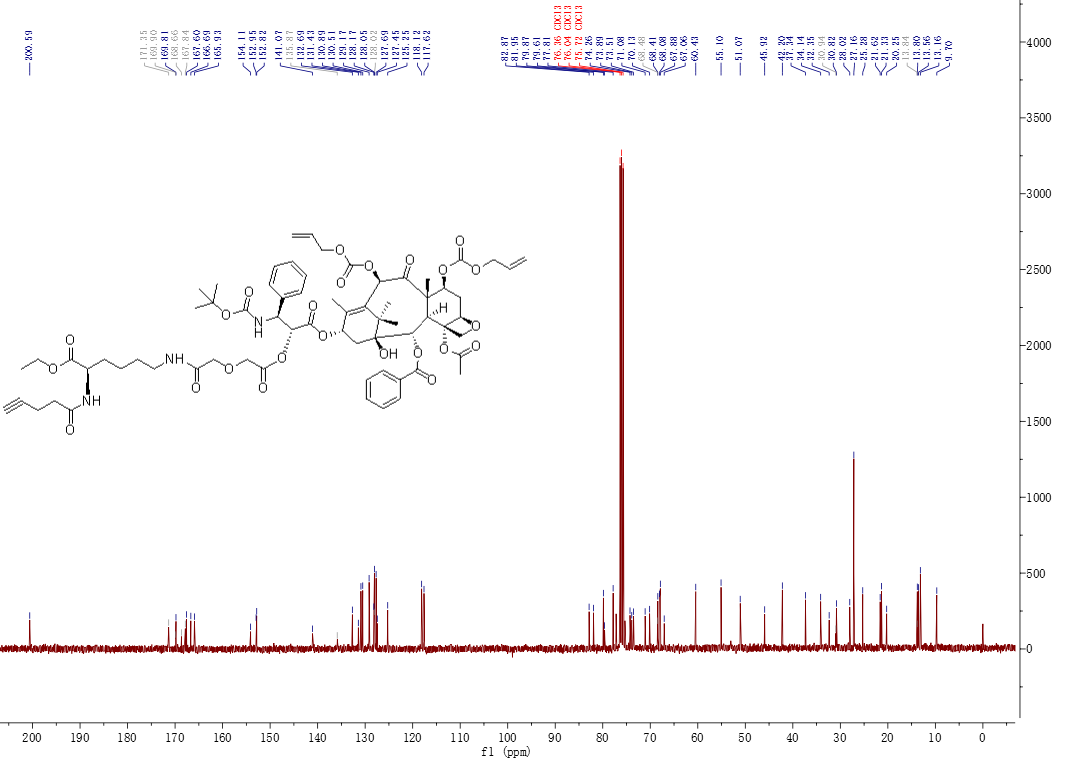

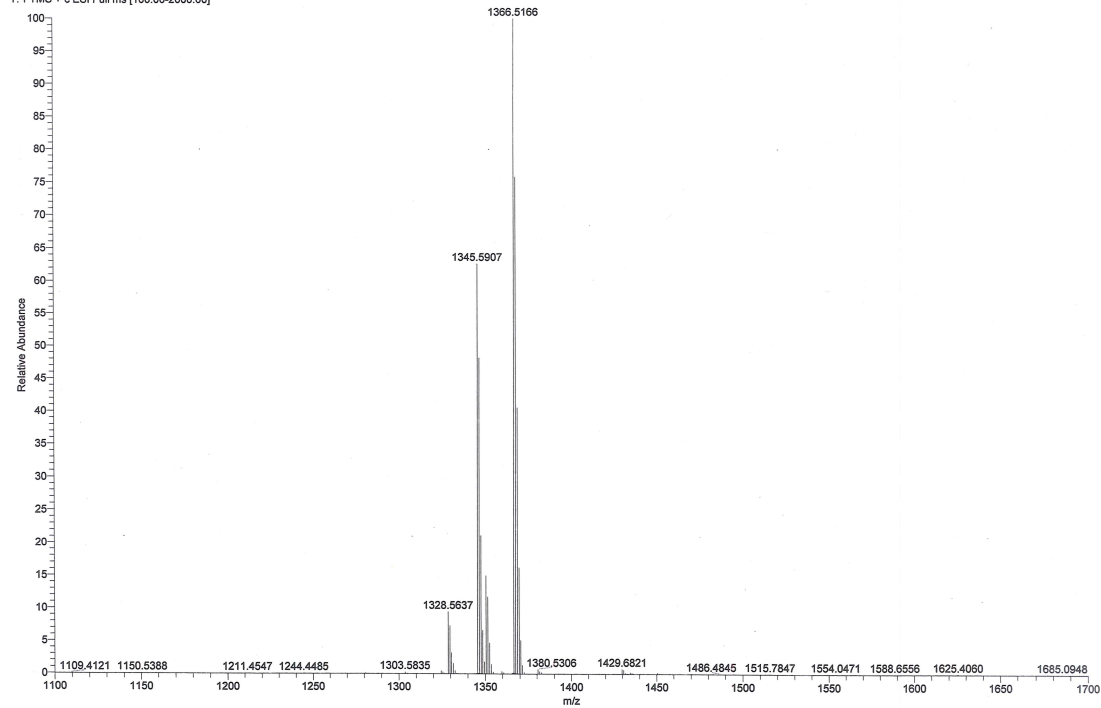


**3.17. ^1^H NMR,^13^C NMR and MS spectra of compound 17**


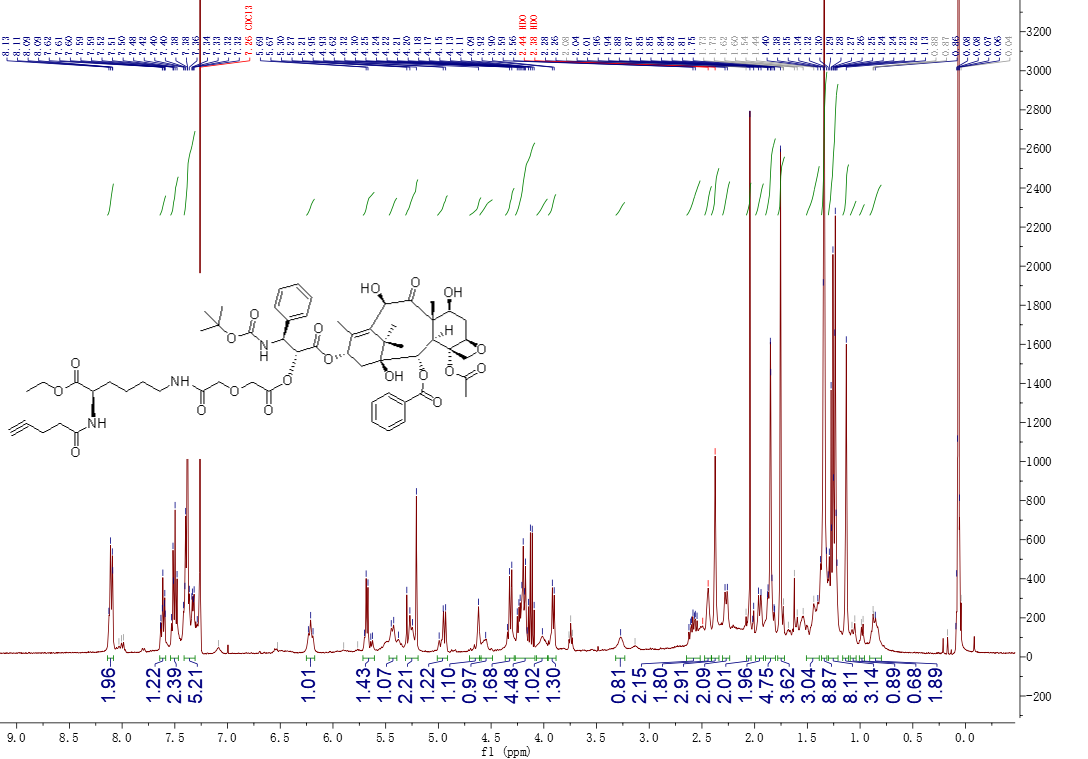


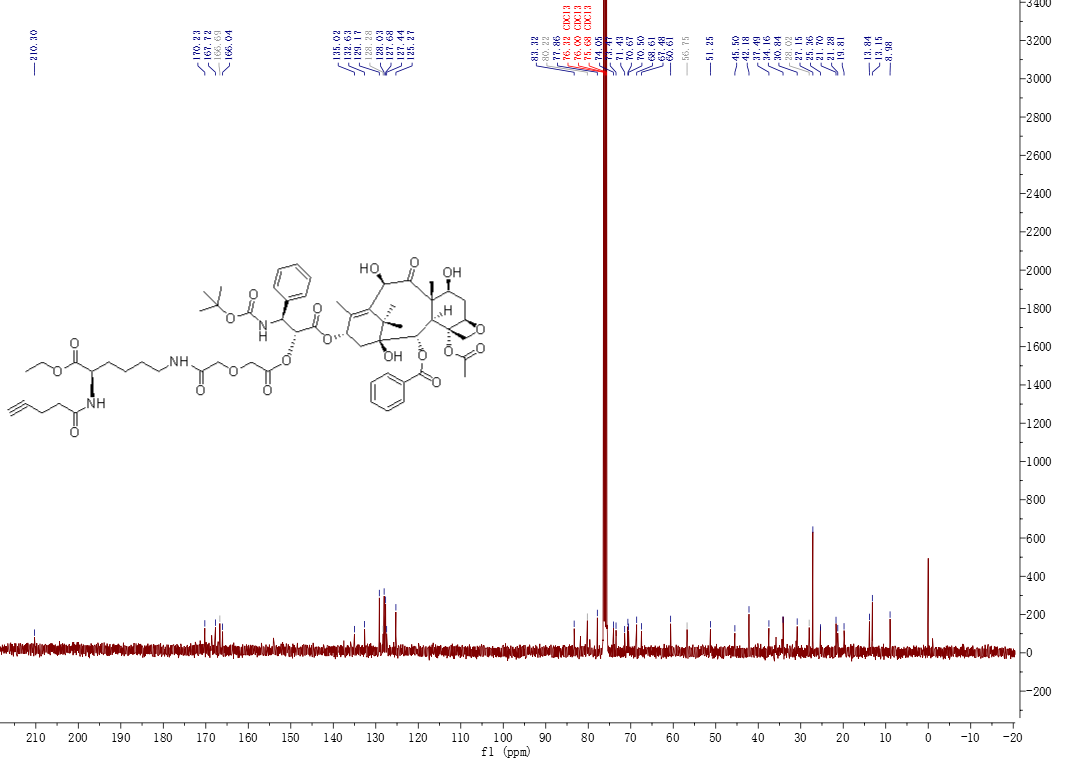


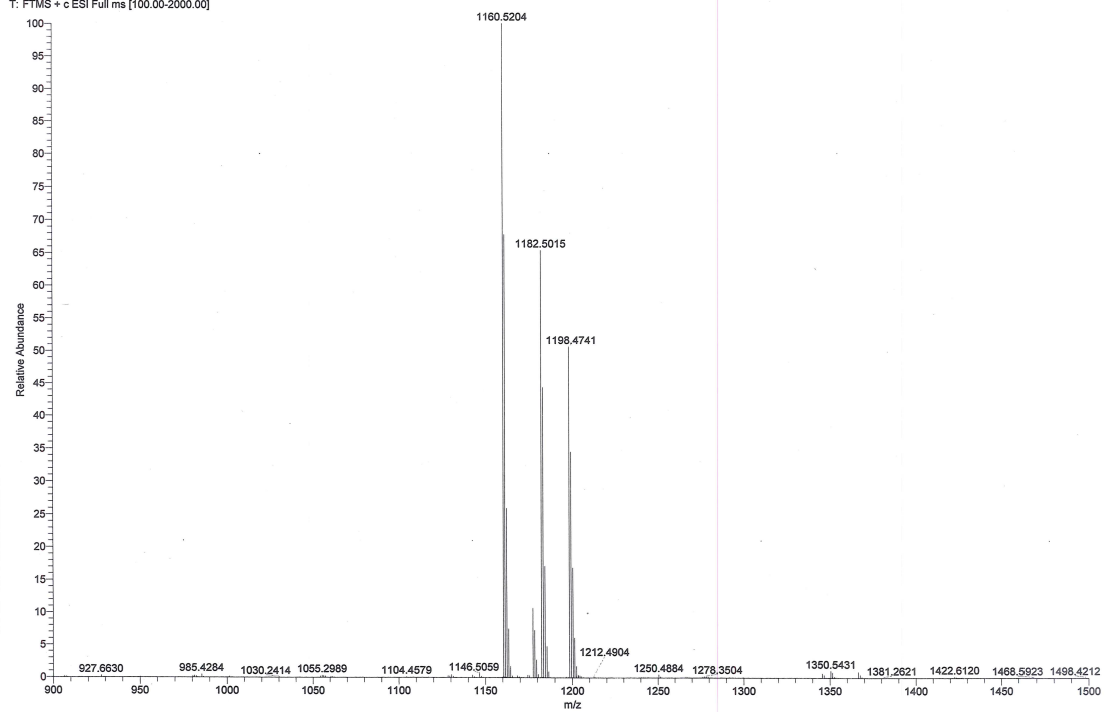


**3.18. ^1^H NMR spectrum of compound 18**


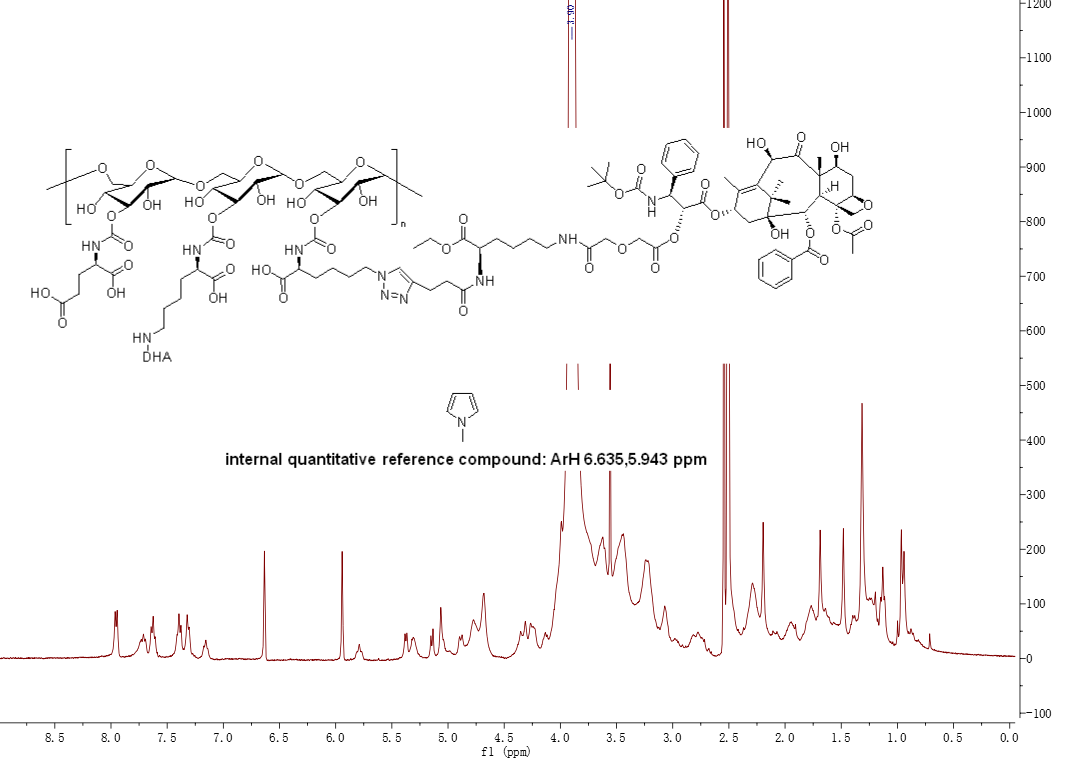

Supplement: Supplemental Material [file IDRD_A_2152133_SM2212.docx]
